# Facile Access to an Active γ‐NiOOH Electrocatalyst for Durable Water Oxidation Derived From an Intermetallic Nickel Germanide Precursor

**DOI:** 10.1002/anie.202014331

**Published:** 2021-02-02

**Authors:** Prashanth W. Menezes, Shenglai Yao, Rodrigo Beltrán‐Suito, J. Niklas Hausmann, Pramod V. Menezes, Matthias Driess

**Affiliations:** ^1^ Department of Chemistry: Metalorganics and Inorganic Materials Technische Universität Berlin Strasse des 17 Juni 135, Sekr. C2 10623 Berlin Germany; ^2^ Institut für Elektrochemie Universität Ulm Albert-Einstein-Allee 47 89081 Ulm Germany

**Keywords:** electroconversion, nickel germanide, oxygen evolution reaction, oxyhydroxide, renewable energy

## Abstract

Identifying novel classes of precatalysts for the oxygen evolution reaction (OER by water oxidation) with enhanced catalytic activity and stability is a key strategy to enable chemical energy conversion. The vast chemical space of intermetallic phases offers plenty of opportunities to discover OER electrocatalysts with improved performance. Herein we report intermetallic nickel germanide (NiGe) acting as a superior activity and durable Ni‐based electro(pre)catalyst for OER. It is produced from a molecular bis(germylene)‐Ni precursor. The ultra‐small NiGe nanocrystals deposited on both nickel foam and fluorinated tin oxide (FTO) electrodes showed lower overpotentials and a durability of over three weeks (505 h) in comparison to the state‐of‐the‐art Ni‐, Co‐, Fe‐, and benchmark NiFe‐based electrocatalysts under identical alkaline OER conditions. In contrast to other Ni‐based intermetallic precatalysts under alkaline OER conditions, an unexpected electroconversion of NiGe into γ‐Ni^III^OOH with intercalated OH^−^/CO_3_^2−^ transpired that served as a highly active structure as shown by various ex situ methods and quasi in situ Raman spectroscopy.

## Graphical abstract

A xanthene‐based bis(germylene*)*‐Ni complex is used as a molecular precursor for the synthesis of ultra‐small NiGe nanostructures, which can generate a γ‐NiOOH structure with intercalated species. This displays exceptional catalytic activity and long‐term durability for the oxygen evolution reaction (OER).

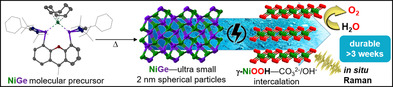

## Introduction

The electrocatalytic oxygen evolution reaction (OER) by water oxidation plays a decisive role in the development of clean renewable energy conversion and storage systems, as it provides electrons that can be used for critical reactions such as to reduce water to hydrogen, carbon dioxide to carbonaceous fuels, as well as metal ions to the corresponding metals in recharging of metal‐air batteries.^[1]^ However, the conversion efficiency of this crucial anodic half‐reaction is limited by the inherently slow reaction kinetics due to the demanding four‐proton coupled electron transfer pathway.^[2]^ In order to lower the kinetic barriers of OER, numerous active electrocatalysts have been investigated for the past decades, however, with limited success. Therefore, recent research is mainly focused on exploring alternative electrocatalysts for water oxidation that are efficient, economic, competitive, yet durable.^[3]^

In the last few years, nickel‐based catalysts have drawn enormous attention not only because of their promising activity and stability in alkaline electrolytes but also due to their function as anodes in large‐scale commercial alkaline electrolyzers.^[4]^ Over the last few years, the suitability of numerous nickel‐based oxidic and non‐oxidic materials has been widely explored for efficient alkaline OER also in the context of structural‐activity relationships.[^4b^, ^5^] The pronounced activity of such Ni‐based electrodes has been ascribed to the presence of nickel oxyhydroxide (NiOOH) species at the surface, which serve as the OER active site.^[6]^ For the non‐oxidic Ni‐based materials (chalcogenides, pnictides, or alloys), it has been shown that the transformation occurs prior to the OER either only at the surface of the electrocatalyst forming core–shell type structures or progressively throughout the bulk ensuring a defect‐rich and high surface area NiOOH.^[7]^ Lately, several strategies including heteroatom doping, morphology and size control, compositional tuning, defect creation as well as interfacial engineering have been successfully utilized to enhance the density of active sites as well as electronic conductivity of Ni‐based electrodes resulting in reasonable OER catalytic performances.^[8]^ Thus, discovering a suitable and novel Ni‐based (pre)catalyst to substantially improve the activity and durability of current OER catalysts remains a formidable challenge and is one of the intensive efforts of current research.^[9]^

Intermetallic nickel phases such as nickel germanides are an important class of metal semiconductors that are well‐known for their high thermal stability as well as their applications in the areas of microelectronics, photovoltaics, magnetism, and thermoelectrics.^[10]^ Among the existing NiGe_x_ phases, nickel monogermanide (NiGe) has been regarded as a highly promising material due to its low electric resistivity (metallic property), enhanced stability in a wide temperature range, low‐temperature synthetic approach, and facile chemical processing.^[11]^ Thus, it is the most favored contact material in complementary to metal‐oxide‐semiconductor devices.[^10a^, ^10d^, ^12^] Until now, however, the synthesis of NiGe nanomaterials is challenging and has been rarely reported.^[10b]^ Further, its application for electrocatalysis has not been perceived so far.

Considering NiGe is a stable intermetallic phase with high intrinsic conductivity with the active catalytic center, we aimed to address in this report the following five research questions: (i) Can we adopt a rational approach to synthesize well defined ultra‐small NiGe nanostructures with high surface area from a molecular precursor?, (ii) will the structure of this intermetallic phase remain stable or is it merely a precursor (precatalyst) in electrochemical alkaline OER?, (iii) what is the structural and functional role of Ge?, (iv) what type of transformation (active structure) can be expected and how does it influence the electrocatalytic OER activity and durability?, and (v) can we provide a guideline for access to other transition‐metal germanides towards OER based on our soft molecular approach?

In the last few years, significant progress has been made in developing various synthetic routes for the preparation of intermetallic compounds towards applications in electrocatalysis.[^4a^, ^13^] A straightforward strategy for the synthesis of nanostructured intermetallics is the low‐temperature molecular precursor approach.^[14]^ The latter enables a good control over the composition, structuring, morphology, and electronic properties of the materials.^[13b]^ Taking advantage of this approach, we have now designed a novel molecular germanium(II)‐nickel(0) precursor complex for the preparation of nanostructured intermetallic NiGe that displayed remarkable catalytic activity and durability in alkaline water oxidation.

**Scheme 1:**
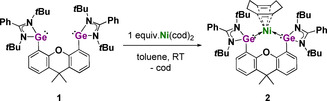
Synthesis of the molecular bis(germylene)‐Ni complex **2** starting from **1**.

Here, we report a premediated synthesis of the first xanthene‐based bis(germylene)‐Ni complex **2** (Scheme 1) and its utilization as a low‐temperature precursor to form monodisperse ultra‐small NiGe nanocrystals. The NiGe has been electrophoretically deposited on nickel foam (NF) and fluorine‐doped tin oxide (FTO) electrodes and applied for alkaline OER for the first time, resulting in substantially low overpotentials surpassing the state‐of‐the‐art Ni‐, Fe, Co, and benchmark NiFe, and noble‐metal‐based catalysts. Strikingly, the NiGe also exhibits superior long‐term stability over three weeks (505 h). A combination of extensive ex situ methods and quasi in situ Raman spectroscopy revealed that NiGe is merely a precatalyst for OER and, under applied potentials, transforms completely into OH^−^/CO_3_^2−^ intercalated γ‐NiOOH that serves as an active catalyst to facilitate O−O bond formation.

## Results and Discussion

### A molecular bis(germylene)Ni precursor to access nickel germanide nanoparticles

Germylenes represent heavy analogues of carbenes that possess a divalent Ge atom with a lone pair of electrons; they can serve as potential ligands towards transition‐metals.^[15]^ Especially, the N‐heterocyclic germylenes (NHGes), featuring a Ge^II^ atom with π‐donor interactions with the neighbor N atoms and/or from an intramolecular Lewis donor, are promising steering ligands akin to the N‐heterocyclic silylene (NHSi) and carbene (NHC) congeners.^[16]^ Taking advantage of the additional chelating effect of bis‐NHGes and bis‐NHSis, we have successfully developed several bis‐NHSis and bis‐NHGes supported transition‐metal complexes for homogeneous catalysis with remarkable reactivity.^[17]^ Given their thermal lability, we envisioned that a bis(NHGe)Ni^0^ complex could act as a suitable single‐source molecular precursor for a low‐temperature synthesis of intermetallic nickel germanides.

### Synthesis and characterization of a xanthene‐based bis(NHGe)Ni^0^ precursor

The xanthene‐based bis(NHGe) **1**, the starting material of molecular bis(NHGe)Ni complex **2**, was prepared in analogy to its silicon homologue (see for the synthesis of **1** Figure S1, Tables S1, and S2 in *Supporting Information*).^[18]^ Treatment of **1** with one molar equivalent of Ni(cod)_2_ (cod=1,5‐cyclooctadiene) in toluene at room temperature resulted in the formation of the bis(NHGe)Ni(cod) complex **2** in 87 % isolated yields as a dark‐brown solid (Scheme 1).

**Figure 1:**
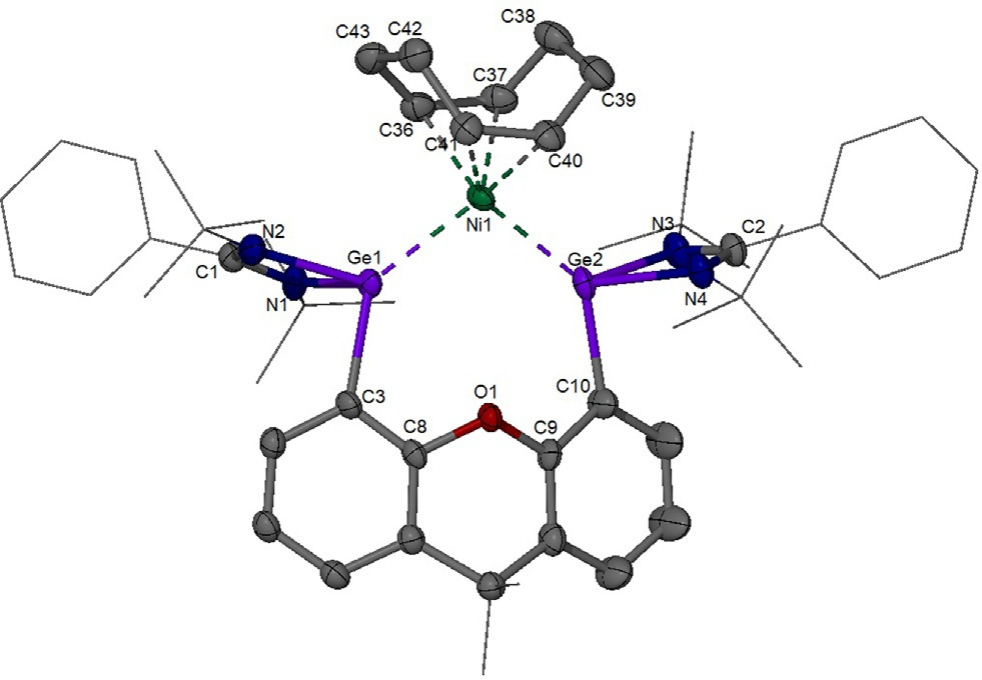
Molecular structure of **2**. The Ge_2_Ni moiety and one of the *tert*‐butyl groups are disordered over two orientations with an occupancy ratio of 0.736:0.27 and 0.54:0.46, respectively. Only the major ones are depicted. Thermal ellipsoids are set at 30 % probability. H atoms are omitted for clarity. CCDC number is given in the Supporting Information.

Compound **2** is diamagnetic and has been unambiguously characterized by ^1^H and ^13^C NMR spectroscopy, high‐resolution mass spectrometry (HRMS), elemental analysis, and IR‐spectroscopy (synthesis of complex **2** in *Supporting Information*). The molecular structure of **2** established by an single‐crystal X‐ray diffraction analysis is depicted in Figure 1. In stark contrast to its silicon homologue,^[18]^ in which the bis(silylene)‐Ni is coordinated by an isomer of cod (1,3‐cyclooctadiene) resulting from 1,5‐cyclooctadiene through proton migration, complex **2** features a 1,5‐cod ligand. Besides the coordination of both C=C bonds of cod, the nickel atom is coordinated by the two germanium(II) centers of the bis(germylene) **1** with a Ge‐Ni‐Ge angle of 101.6(2)°. The Ni1‐Ge1 [2.315(4) Å] and Ni1‐Ge2 [2.311(4) Å] distances are similar to the sum of theoretically predicted covalent radii for Ni‐Ge single bonds [2.31 Å].^[19]^ The crystal data, refinement parameters, and selected interatomic distances of **2** are given in Tables S3 and S4.

### Synthesis and structural characterization of nanostructured NiGe

**Figure 2:**
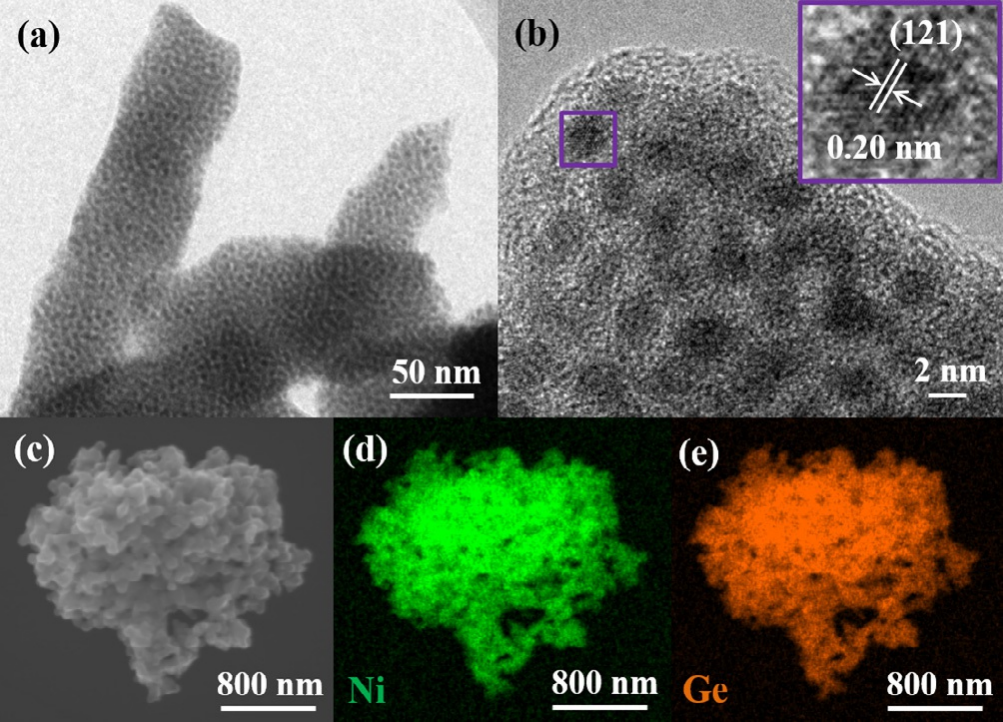
Structural investigation of intermetallic NiGe. a) TEM image displaying assembly of monodispersed ultra‐small nanostructures, b) HR‐TEM image exhibiting approximately 2 nm sized particles with a well‐resolved lattice spacing of 0.20 nm corresponding to the crystallographic (121) plane of NiGe (JCPDS 7‐297), c) SEM image and, d),e) EDX elemental mapping showing homogenously distributed Ni (green) and Ge (orange) in the structure of NiGe.

The molecular complex **2** was subjected to thermolysis under hot‐injection conditions at 250 °C in oleylamine to produce black powdered NiGe with ≈75 % yields (see *Supporting Information*). Note that a NiGe_2_ phase was expected from the precursor Ni:Ge ratio, however, NiGe_2_ is a metastable phase and does not exist in the Ni‐Ge phase diagram (Figure S2).^[20]^ Hence, a thermodynamically stable NiGe phase is formed. The phase‐purity, morphology, microstructure, composition, surface area, and the electronic state of NiGe were investigated by powder X‐ray diffraction (PXRD), scanning electron microscopy (SEM) and transmission electron microscopy (TEM), energy‐dispersive X‐ray spectroscopy (EDX), elemental mapping, inductively coupled plasma atomic emission spectroscopy (ICP‐AES), elemental analysis, Fourier‐transform infrared spectroscopy (FT‐IR), Brunauer‐Emmett‐Teller (BET), and X‐ray photoelectron spectroscopy (XPS). The detailed description of the as‐prepared NiGe is provided in Figure 2, Figure S3–S11, Table S5.

### Electrocatalytic OER activity

The OER catalytic performance of the prepared NiGe materials was evaluated in aqueous 1 M KOH solutions using a three‐electrode cell. Prior to the OER measurements, the NiGe was deposited on the high surface area, low‐cost, conductive, and 3D open porous nickel foam (NF) as well as on fluorinated tin oxide (FTO) by electrophoretic deposition (EPD) that directly served as working electrodes. Subsequently, the as‐deposited films were also characterized extensively which confirmed preservation of the chemical identity (stability) of NiGe on the electrode substrates (Figures S12–S19). To have a fair comparison of its catalytic OER activity, the literature reported highly active Ni‐based catalysts (NiOOH and Ni(OH)_2_) were also synthesized (Figure S20) and deposited through EPD on both NF and FTO under identical conditions with the same mass loading (≈1 and 0.4 mg cm^−2^ for NF and FTO, respectively).

**Figure 3:**
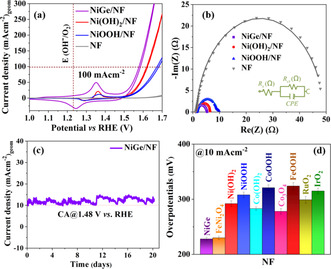
Catalytic OER performances of NiGe and the Ni‐based reference materials deposited on NF in 1 M aqueous KOH solution. a) Cyclic voltammetry curves (1 mV s^−1^), b) Nyquist plot constructed from EIS experiments at 1.51 V versus RHE and fitted to an equivalent circuit (shown in the inset), c) long‐term stability curves of NiGe over 21 days, and d) bar diagram (with error bars) showing the overpotential (10 mA cm^−2^) of NiGe with various state‐of‐the‐art Co‐, Fe‐, Ni, and NiFe, as well as noble‐metal‐based catalysts measured in identical condition with the same mass loading.

As shown in Figure 3 a, the cyclic voltammetry (CV) curves revealed that the NiGe/NF electrode displayed substantially enhanced OER activity compared to that of the as‐deposited Ni(OH)_2_/NF and NiOOH/NF while the bare NF (subjected to the same EPD protocol) exhibited a limited activity. Remarkably, the overpotential of the NiGe/NF electrode was only 228±3 mV at a current density of 10 mA cm^−2^, which is lower than those of Ni(OH)_2_/NF (292±5 mV), NiOOH/NF (308±5 mV) and bare NF (485±9 mV). Prior to the onset of OER, all measured Ni materials showed distinct redox peaks between 1.1–1.5 V vs. reversible hydrogen electrode (RHE) associated with the oxidation of Ni^II^ → Ni^III^ [Ni(OH)_2_ + OH^−^ → NiOOH + H_2_O + e^−^] that has widely been regarded as the catalytically active site for OER.[^4a^, ^21^] The broad redox feature of NiGe/NF further suggested the oxidation of Ni occurs with the concomitant loss of Ge from the structure (see below). Moreover, the OER kinetics of investigated materials was examined by Tafel analysis.^[22]^ The Tafel plots were calculated by their corresponding LSV polarization curves and are shown in Figure S21. Impressively, NiGe/NF exhibits the lowest Tafel slope of 56±2 mV dec^−1^ compared to Ni(OH)_2_/NF (79±1 mV dec^−1^) and NiOOH/NF (83±1 mV dec^−1^) suggesting a more effective electron transfer, highly efficient reaction kinetics, and efficient catalytic OER activity of nanostructured NiGe/NF.

Electrochemical impedance spectroscopy (EIS) was conducted to offer insights into the electrode kinetics during the OER process.^[23]^ The charge‐transfer resistance (*R*_ct_) across the electrode/electrolyte interface was obtained from the Nyquist plot (Table S6). As shown in Figure 2 b, the *R*_ct_ of NiGe/NF was lower than those of the other three Ni‐electrodes, demonstrating its faster charge transport leading to an improved electrocatalytic OER activity. One of the most important criteria to evaluate the performance of the catalysts for practical applications in water splitting is their long‐term durability under strongly alkaline operating conditions.^[3a]^ Strikingly, the OER chronoamperometric measurements (OER CA) of NiGe/NF electrocatalyst displayed favorable operating stability, providing a stable 10 mA cm^−2^ current density at a potential of 1.48 V vs. RHE for a period of 24 h (Figure S22). Motivated by this, we further extended the durability measurements over three weeks that showed unceasing stability and sustainability of the NiGe/NF without any drop in the current density signifying its capability as an efficiently stable anode for alkaline water electrolysis (Figure 2 c). Besides, a Faradaic efficiency of 95±2 % for OER was calculated by quantifying the evolved O_2_ (gas) during electrolysis that matched well with the theoretically calculated values (Table S7). As the large‐scale alkaline electrolyzers are operated at elevated temperature,^[24]^ we conducted a chronopotentiometric measurement at 100 mA cm^−2^ at 65 °C for 20 h that exhibited a stable potential curve supporting its usability at higher current densities (Figure S23).

In order to assess the origin of the greatly enhanced water oxidation activity, the double‐layer capacitance (*C*_dl_) was determined, which is proportional to the electrochemically active surface area (ECSA).^[25]^ The characteristic CV curves of presented materials with different scan rates in a non‐faradaic region are shown in Figure S24. The corresponding capacitive current value as a function of the scan rate was used to calculate the *C*_dl_. A *C*_dl_ value of 1.36±0.04 mF cm^−2^ was attained for NiGe/NF while much lower values, 0.85±0.02, 0.79±0.01, and 0.64±0,01 mF cm^−2^ were obtained for Ni(OH)_2_/NF, NiOOH/NF and NF, respectively (Figure S25), suggesting that more catalytically active sites of NiGe/NF are available for the OER process. It is known from previous OER studies that non‐oxide phases can undergo severe corrosion in strongly alkaline conditions leading to a partial or complete loss of metal/metalloid.[^13c^, ^13d^, ^13e^, ^13f^, ^26^] To verify this, we measured the *C*_dl_ of NiGe/NF after 24 h of OER CA giving rise to a value of 2.82±0.04 mF cm^−2^, which is twice larger than the as‐deposited NiGe/NF (Figure S26). The increase in *C*_dl_ (or ECSA) is an indication of the rapid loss of Ge into the electrolyte with significant structural transformation. Furthermore, the currents of NiGe/NF, Ni(OH)_2_/NF, and NiOOH/NF catalysts were normalized by (i) geometric area, (ii) mass, (iii) *C*_dl_ (ECSA), and (iv) BET surface area and presented in Figure S27 that supported the derived conclusions (Table S8). Most importantly, it is well known that the comparison of overpotentials is only meaningful if the catalysts are tested in identical conditions with optimized parameters (mass loading, *iR* correction, geometric area, type of electrode substrate, set‐up design, etc.).^[27]^ Taking this into account, we synthesized, electrodeposited, and compared activities of several non‐noble metal‐based state‐of‐the‐art electrocatalysts with Co‐, Fe‐ and NiFeO_x_ as well as commercial IrO_2_ and RuO_2_ for the alkaline OER in identical conditions (Figures S28–S35). Remarkably, the obtained OER overpotentials of these catalysts were still substantially higher than the one of NiGe/NF (Figures 2 d and S36). In addition, the OER activity comparison of the presented catalysts with those of reported benchmark NiFe‐ or Ni‐based catalysts are listed in Tables S9 and S11.

After achieving encouraging results on NF, we further investigated the electrocatalytic OER performance of the same materials deposited on FTO, primarily to understand the inherent role of the electrode substrates influencing the catalytic activity. The LSV profiles of NiGe/FTO, Ni(OH)_2_/FTO, NiOOH/FTO, and FTO are shown in Figure S37. Similar to the high activity and low overpotential demonstrated on NF, the NiGe/FTO required only an overpotential of 322±2 mV to yield a current density of 10 mA cm^−2^, which is much smaller than those required for Ni(OH)_2_/FTO (380±6 mV) and NiOOH/FTO (444±6 mV). As observed in the case of NiGe/NF, the CV of NiGe/FTO also featured a redox pair evidencing that Ni^II^ is reversibly converted to Ni^III^ (NiOOH) which serves as the active site for OER (Figure S38).^[21a]^ The conducted EIS and ECSA measurements on NiGe/FTO, Ni(OH)_2_/FTO, and NiOOH/FTO exhibited accelerated charge transfer and higher density of active sites for NiGe/FTO compared to the other investigated reference catalysts (Figures S39, S40 and Table S10). The OER CA response of NiGe/FTO was examined at 1.58 V vs. RHE for 24 h, which displayed excellent stability of the electrocatalyst (Figure S41). Furthermore, the normalized current densities followed the same trend as that of deposited catalysts on NF (Figure S42). Finally, the superiority of NiGe/FTO was illustrated by comparing the overpotentials of the as‐deposited non‐noble and noble‐metal‐containing catalysts on FTO as well as literature reported Ni‐based catalysts (Figures S43–S45, Table S9).

### Ex situ post‐catalytic characterization

**Figure 4:**
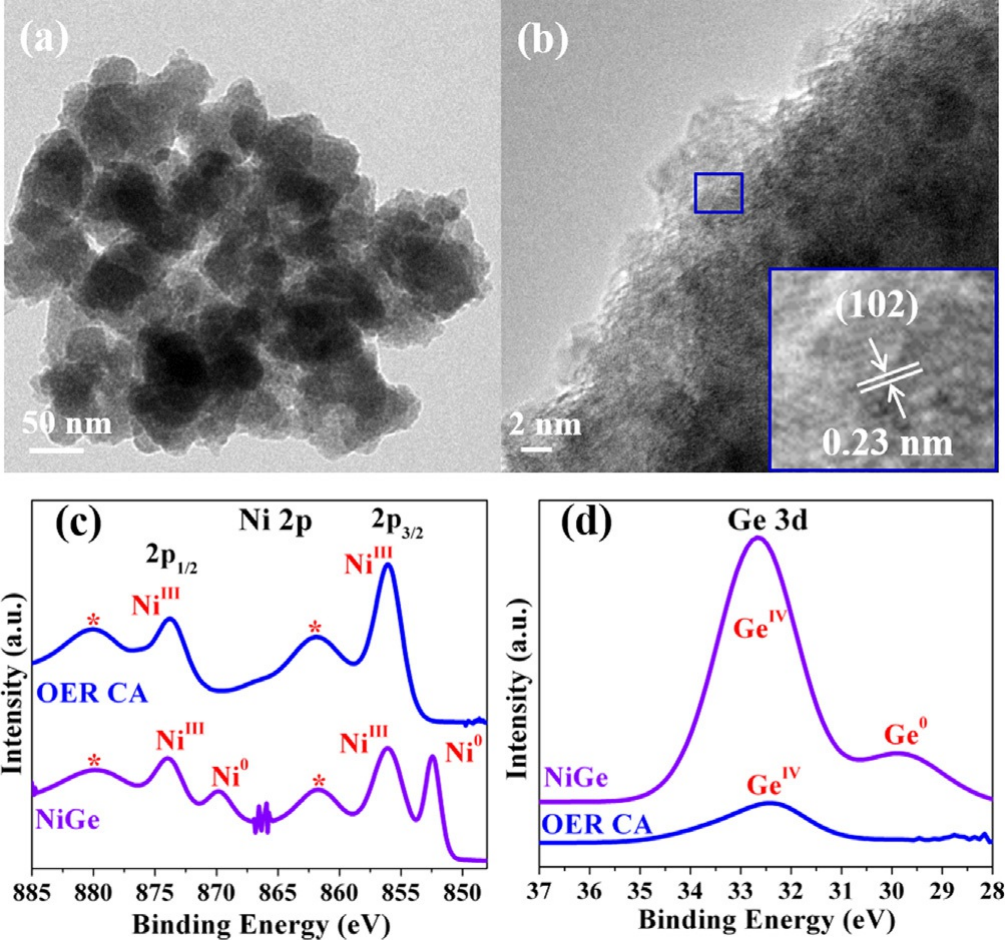
Post‐OER (CA 24 h) analysis of fully elecrooxidized NiGe. a) TEM image showing porous‐type morphology, b) HR‐TEM image with the lattice fringes (inset) of 0.23 nm corresponding to (102) plane of γ‐NiOOH (JCPDS 6‐75), c) Ni 2p XPS spectrum displaying complete oxidation of Ni^0^ (NiGe) to Ni^III^ (γ‐NiOOH), and d) Ge 3d XPS confirming the exclusion of Ge from the NiGe structure.

To gain an in‐depth insight into the bulk and surface structure and to uncover the nature of active species for determining a structure–activity relationship, the post‐OER (CA 24 h) NiGe/FTO electrode was extensively characterized through diffraction, microscopic, spectroscopic, and analytical methods. The PXRD pattern of NiGe/FTO after OER exhibited a broad diffraction pattern similar to as‐prepared material indicating the small particle size (Figure S46 and S47). Interestingly, the SEM images of NiGe after OER showed porous‐type morphology with an agglomeration of particles (Figure S48). The EDX mapping of the film exhibited an almost total loss of Ge (over 95 %) and concomitant incorporation of O into the structure that shows homogeneous dispersion of Ni, suggesting complete corrosion of the NiGe phase to an OER active NiO_x_H_y_ structure (Figure S49). The percentage of distribution of the elements derived by EDX mapping is also consistent with the result acquired from the ICP‐AES and XPS analysis (Figures S50 and S51, Table S5). As observed for SEM, the TEM images showed a rigorous transformation of initial NiGe into a hollow nanostructure (Figure 4 a and Figure S52a–c). A closer look at the nanostructures in the HR‐TEM images exhibited lattice fringes of 0.23 nm that can be assigned to (102) plane of γ‐NiOOH (JCPDS 6–75) (Figure 4 b, inset). More importantly, the SAED pattern produced weak diffraction rings at 0.34, 0.23, and 0.21 nm for (006), (102), and (105) crystallographic planes, respectively, corresponding to the γ‐NiOOH structure (Figure S52d). With this information in hand, we evaluated the FT‐IR spectra of OER CA that showed the appearance of new IR bands compared to the initial NiGe phase (Figure S53). Notably, the band at 570 cm^−1^ can directly be corroborated to Ni^III^‐O stretching vibrations of γ‐NiOOH and is a representative band for the literature reported phases of γ‐NiOOH.^[28]^ Surprisingly, a new band at 1382 cm^−1^ was observed too that can be attributed to carbon‐oxygen stretching vibrations of CO_3_^2−^.^[29]^ The carbonate anion originates from the KOH electrolyte that consumes CO_2_ from ambient air.^[30]^ Such intercalation of carbonate anions between interlayers of γ‐NiOOH has shown to be beneficial to enhance the OER activity.[^28c^, ^30^] Besides, IR bands responsible for surface hydroxylation were also evident.[^28b^, ^28c^]

The surface valence states of fully converted NiGe after OER was obtained by XPS analysis (Figure S54a). The Ni 2p_3/2_ and Ni 2p_1/2_ spectrum displayed sharp peaks at the binding energy of 856.0 and 873.7 eV (along with two typical satellite peaks) that can only be ascribed to Ni^III^ of the structure, while the peaks responsible for Ni^0^ were absent, confirming the complete electrooxidation of NiGe surface to γ‐NiOOH under alkaline OER (Figure 4 c).[^21b^, ^28a^, ^28c^, ^31^] Moreover, the binding values obtained here are typical of γ‐NiOOH phases reported in the literature.[^28a^, ^32^] The Ge 3d spectrum did not show any peak corresponding to Ge^0^, indicating the oxidation of the material (Figure 4 d). The deconvoluted peaks at 32.2 and 33.3 eV corresponding to Ge 3d_5/2_ and 3d_3/2_, which could be due to the Ge^IV^O_2_ generated from the adsorption at the film surface (Figure S54b).^[33]^ The O 1s spectrum was deconvoluted into two peaks, at ≈531.0 eV and 531.7 eV consistent with the formation of γ‐NiOOH (Figure S54c).^[34]^ As it has been shown that Fe from the electrolyte can be incorporated into the Ni‐based catalysts, we examined the XPS survey spectrum (Figure S55) to detect the presence of Fe on the surface of the films. The spectrum was inconclusive due to the overlapping Fe 2p and Sn 3p signals, and therefore, ICP‐AES was conducted that showed a minute amount of Fe (<0.4±0.1 %) after 24 h of CA.

To address the question on the dissolution rate of Ge into the electrolyte and extent of phase transformation in OER conditions, we also examined the NiGe/FTO film after initial CV (OER CV, 3 cycles) measurements (Figures S56–S60, Table S5) by SEM, EDX, elemental mapping, ICP‐AES, FT‐IR, and XPS analysis. Surprisingly, the OER CV characterization results matched exactly with that of OER CA demonstrating that the electroconversion of NiGe to γ‐NiOOH is an instantaneous process. Besides, OER CV and CA, the NiGe/NF electrodes were also characterized after three weeks (505 h) of durability tests. As anticipated, the obtained SEM, EDX, elemental mapping, and XPS mapping results were found to be similar to that of CV and CA experiments further confirming the porous nature of electrodes, the absence of Ge in the structure as well as the formation of Ni^III^ throughout the bulk (Figures S61–S64). Thus, from the ex situ characterizations at various time intervals of OER, it was apparent that the NiGe suffers spontaneous restructuring under an applied bias finally forming a γ‐Ni^III^OOH as the catalytically active structure. According to the Pourbaix diagrams,^[35]^ the structural conversion of NiGe to γ‐NiOOH under alkaline OER can be explained via oxidative leaching of Ge in the form of deprotonated germanic acid (GeO_3_^2−^), and subsequent oxidation of nickel [from Ni^δ+^ to Ni^III^, Eq. 1], which should proceed through the following equation:

(1) $$\mathrm{NiGe}\;+\;9\;\mathrm{OH}{}^{-}\;\to \;\mathrm{Ni}{}^{\mathrm{III}}\mathrm{OOH}\;+\;\left[\mathrm{Ge}{}^{\mathrm{IV}}O{}_{3}\right]{}^{2-}\;+\;7\;e{}^{-}\;+\;4\;H{}_{2}O$$

 

### Quasi in situ and ex situ Raman spectroscopy

**Figure 5:**
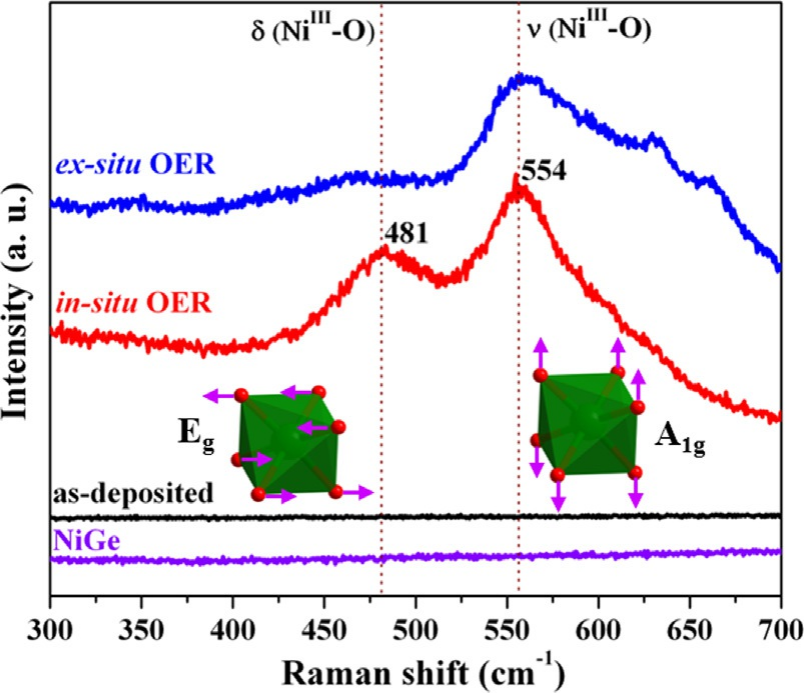
Quasi in situ and ex situ Raman spectroscopy of NiGe (OER CA, 24 h) on FTO collected at a potential of 1.58 V versus RHE in alkaline electrolyte. The stretching A_1g_ and bending E_g_ vibrational modes of γ‐NiOOH is represented by green octahedra. For comparison, the Raman spectra of the as‐prepared and as‐deposited (inactive) NiGe are also shown.

To uncover the active structure under quasi in situ conditions, we performed (resonance) Raman spectroscopy of NiGe, which is a very sensitive probe to the surface structure of the catalysts. The as‐prepared NiGe powder and the as‐deposited NiGe film did not show any distinguishable features indicating that NiGe does not possess any Raman allowed bands. We then performed quasi in situ Raman measurements on the NiGe film treated for 24 h of OER CA where Raman bands at 481 and 554 cm^−1^ were observed.[^4f^, ^5c^, ^36^] By comparing with the reported γ‐NiOOH structure, the first band at 481 cm^−1^ is clearly associated with the depolarized E_g_ mode (bending) whereas the band at 554 cm^−1^ can be assigned to the polarized A_1g_ mode (stretching).[^28c^, ^34b^, ^37^] From Figure 5, one can note that for the E_g_ mode the oxygen atoms vibrate along the plane while in the A_1g_ mode they vibrate perpendicular to the plane.^[38]^ In addition to this, a broad band between 850 cm^−1^ and 1200 cm^−1^ also appeared that has been previously attributed to ν(O‐O) of an active oxygen species (NiOO^−^) in oxyhydroxide structure (Figure S65).[^4f^, ^36^, ^37c^, ^38^] Very similar features were also observed for the ex situ Raman spectrum of NiGe film after OER CA with additional small bands at 630–660 cm^−1^ that can be attributed to the presence of oxygen vacancies.^[39]^

### Insights into the active structure

**Figure 6:**
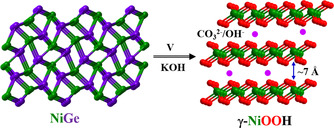
Crystal structure of NiGe and electrochemically generated γ‐NiOOH. Under applied potential (V) in an alkaline KOH electrolyte, a complete dissolution of Ge was observed forming γ‐NiOOH. From the large interplanar spacing, it can be seen that the ionic intercalation of OH^−^/CO_3_^2−^ is favored for the OER process that is reflected in higher ECSA values.

Nickel‐(oxy)hydroxides, in particularly, α‐Ni(OH)_2_, β‐Ni(OH)_2_, β‐NiOOH, and γ‐NiOOH have widely been utilized as cathodes in primary and secondary alkaline batteries as well as supercapacitors.^[40]^ The phase transformation mechanism of these materials during the electrochemical redox process or aging was described by Bode's diagram,^[41]^ according to which the γ‐NiOOH can be achieved by the oxidation of α‐Ni(OH)_2_ or overcharging of β‐NiOOH (Figure S66).[^28b^, ^32^, ^42^] The α to γ transformation involves more than one electron transfer, and the maximal oxidation state of Ni, in this case, is limited to +3.6.[^42^, ^43^] Most importantly, the crystal structure of γ‐NiOOH comprises of a large interlayer spacing of ≈7 Å and contains intercalated species such as water or ions that are absorbed between the layers.^[28b]^ Such large interlayer spacing in γ‐NiOOH has shown to favor the ionic intercalation of OH^−^ or CO_3_^2−^ (from the dissolved electrolyte) anions to expose a large number of active sites for the evolution of oxygen (Figure 6).[^28c^, ^30^] Our results are also consistent with the previous study on the role of interlayer anions in NiFe‐ or Ni‐based layered double hydroxides (LDH) where the highest catalytic OER activity can be achieved when hydroxide/carbonates species are present in the interlayers of LDH.[^28c^, ^30^] Therefore, the enhanced performance of γ‐NiOOH derived in situ from NiGe, compared to the as‐prepared and other literature known NiOOH electrocatalysts,^[4b]^ can be attributed to the structural flexibility of Ni sites through the generation of (defected) structure with ionic intercalation of OH^−^/CO_3_^2−^ between the large interplanar spacing of γ‐NiOOH, higher ECSA values, better electronic conductivity, superior OER kinetics, and an accelerated charge transfer resistance to facilitate OER.

## Conclusion

We have successfully addressed the research questions (i)–(v) as mentioned above in the introduction. With respect to question (i), a rational protocol to synthesize well‐defined ultra‐small NiGe nanoparticles could be achieved, starting from a new Ni‐Ge molecular precursor. To answer the questions (ii)–(v), the as‐prepared NiGe was electrophoretically deposited on NF and FTO and investigated for the alkaline OER for the first time. The catalytic activity and stability of the NiGe was found to be strikingly higher than that of benchmarked Ni‐, Fe‐, Co‐, and NiFe‐based catalysts in identical conditions. Under electrochemical conditions, a vigorous electroconversion of NiGe occurred indicating that the NiGe is indeed a precatalyst for OER. The dissolution of Ge further confirmed the insignificant structural or functional role of Ge for OER, however, it is essential to direct the structure to form the most active catalyst. From the combination of advanced ex situ and quasi in situ Raman spectroscopy, it is demonstrated that the transformation of NiGe is instantaneous, forming a highly‐active γ‐NiOOH phase that acts as the most active structure to facilitate O_2_ evolution. It could be expected that the large interplanar spacing of γ‐NiOOH provides the ionic intercalation of OH^−^/CO_3_^2−^, which is favored for the OER process as reflected in higher ECSA values. We believe that the insights offered in this study can easily be generalized to the other transition metal germanides that are also expected to follow the same transformation in alkaline media as that of NiGe. The presented molecular approach might be referenced to investigate and explore a large number of unexplored class of solid‐state intermetallic materials for water splitting that otherwise are impossible to attain in their nano‐form.

## Conflict of interest

The authors declare no conflict of interest.

## Supporting information

As a service to our authors and readers, this journal provides supporting information supplied by the authors. Such materials are peer reviewed and may be re‐organized for online delivery, but are not copy‐edited or typeset. Technical support issues arising from supporting information (other than missing files) should be addressed to the authors.

Supplementary
