# Supplementary material for: Facile Access to an Active γ‐NiOOH Electrocatalyst for Durable Water Oxidation Derived From an Intermetallic Nickel Germanide Precursor
Source: Angew Chem Int Ed Engl. 2021 Feb 2;60(9):4640–7. doi: 10.1002/anie.202014331 (PMC7986911; doi:10.1002/anie.202014331)
Supplement: Supplementary file 1 — Supplementary [file ANIE-60-4640-s001.pdf]

Angewandte Chemie
Eine Zeitschrift der Gesellschaft Deutscher Chemiker

Supporting Information

Facile Access to an Active γ-NiOOH Electrocatalyst for Durable Water Oxidation Derived From an Intermetallic Nickel Germanide Precursor

Prashanth W. Menezes[+], Shenglai Yao[+], Rodrigo Beltrán-Suito[+], J. Niklas Hausmann, Pramod V. Menezes, and Matthias Driess**

anie_202014331_sm_miscellaneous_information.pdf

Supporting Information

Table of Contents

Contents	Page number
1. Materials	2
2. Instrumentations	2-4
3. Synthesis of molecular complexes	4-5
4. Synthesis of the catalysts	6-7
5. Electrophoretic deposition on NF and FTO	7
6. Electrochemical measurements	7-8
7. Crystal structure determination of complexes	9-12
8. Ni-Ge phase diagram	13
9. Characterization of as-prepared NiGe	14-20
10. Characterization of as-deposited NiGe	21-26
11. Characterization of Ni-references	27
12. Electrochemical OER activity on NF	28-35
13. Characterization of non-noble metal-based materials	36-38
14. Electrochemical OER activity of NF	38
15. Characterization of noble metal-based materials	39
16. Electrochemical OER activity on NF	40
17. Electrochemical OER activity on FTO	41-47
18. *Ex-situ* post-characterization after OER CA (24 h)	48-55
19. *Ex-situ* post-characterization after OER CV	56-59
20. *Ex-situ* post-characterization after OER CA (21 days)	60-63
21. *Quasi in-situ* Raman spectroscopy	64
22. Bode diagram	65
23. Comparison of OER activity of NiGe with Ni-based catalysts	66
24. References	67-70

Chemicals and Materials

The starting material 4,5-dibromo-9,9-dimethylxanthene[1] and chlorogermylene $PhC(tBuN)_2GeCl$[2] were prepared according to literature procedures. Bis(cyclooctadiene)nickel(0) and sec-butyllithium solution (1.4 M) in cyclohexane were purchased from Sigma-Aldrich. Solvents were dried by standard methods and freshly distilled prior to use.

1 M aqueous potassium hydroxide (KOH; Fe<0.05 ppm determined by ICP-AES), oleylamine ($CH_3(CH_2)_7CH{=}CH(CH_2)_7CH_2NH_2$, b.p. ~350°C), and other reagents used in the synthetic procedures were obtained from Sigma Aldrich. The commercial ruthenium oxide (RuO_2; 99%), iridium oxide (IrO_2; 99%), and cobalt oxide (Co_3O_4) were purchased from Alfa Aesar. The electrode substrate nickel foam (NF) and fluorine-doped tin oxide (FTO, resistivity 8−12 Ω/sq) were obtained from Recemat BV and Sigma Aldrich, respectively.

Instrumentations

All molecular chemistry experiments were carried out under dry oxygen-free nitrogen using standard Schlenk techniques or an MBraun glove box fitted with a gas purification and recirculation unit.

The nuclear magnetic resonance **(**NMR**)** spectra were recorded with Bruker spectrometers ARX200 and AV400 referenced to residual solvent signals as internal standards. Abbreviations: s = singlet; d = doublet; t = triplet; sept = septet; m = multiplet; br = broad.

Elemental analyses were performed by the analytical labor service in the Institute of Chemistry, Technical University of Berlin, Germany. The High-resolution ESI-MS were measured on a Thermo Scientific LTQ orbitrap XL.

Powder X-ray diffraction (PXRD) patterns were measured on a Bruker AXS D8 advanced automatic diffractometer equipped with a position-sensitive detector (PSD) and curved germanium (111) primary monochromator using Cu Kα radiation (λ = 1.5418 Å). The PXRD of the FTO deposited electrodes were measured using Bragg-Brentano geometry under air, using a Rigaku *SmartLab 3 kW* diffractometer (Rigaku Corporation, Japan) with Cu-*Kα* (λ = 1.5418 Å radiation. Data acquisition was carried out using the *SmartLab Guidance* software package (Rigaku Corporation, Japan; Version 2.1.0.0.

The inductively coupled plasma atomic emission spectroscopy (ICP-AES) was carried out on a Thermo Jarrell Ash Trace Scan analyzer. The presented materials were digested in aqua regia $HCl:HNO_3$ 3:1 v/v (nitric acid, SUPRA-Qualität ROTIPURAN® Supra 69% and hydrochloric acid, SUPRA-Qualität ROTIPURAN® Supra 30%) and the average of three reproducible independent experiments were presented. The digestion volume (2.5 mL) was diluted with Milli-Q water up to 15 mL. For the post-OER investigations, the electrolyte solutions were analyzed as obtained. Calibration curves were prepared for both nickel and germanium with concentrations between 1 mgL^{-1} and 100 mgL^{-1} from standard solutions (1000 mgL^{-1} single-element ICP-Standard Solution ROTI®STAR).

Fourier-transform infrared spectroscopy (FT-IR) was examined using a BIORAD FTS 6000 FT-IR spectrometer under attenuated total reflection (ATR) conditions. The data were recorded in the range of 500–4000 cm^{-1} with an average of 32 scans at 4 cm^{-1} resolution. For

the molecular complexes, the IR spectra were measured with a Nicolet iS5 FT-IR-Spectrometer from the company of Thermo Scientific.

To gather information on the morphology, and the surface structures, the scanning electron microscopy (SEM) was carried out on an LEO DSM 982 microscope integrated with EDX (EDAX, Apollo XPP). Data handling and analyses were attained with the software package EDAX.

The microstructure investigations of the materials were achieved by transmission electron microscopy (TEM), which was explored on an FEI Tecnai G2 20 S-TWIN transmission electron microscope (FEI Company, Eindhoven, Netherlands) and JEOL 2100 electron microscope equipped with a LaB_6 source at 200 kV acceleration voltage. For the analysis of the films after electrocatalysis, the films were scraped from the electrode substrate and transferred onto a carbon-coated copper grid. EDX analyses were achieved with an EDAX r-TEM SUTW detector (Si (Li) detector), and the images were recorded with a GATAN MS794 P CCD camera. The SEM and TEM experiments were conducted partially at the Zentrum für Elektronenmikroskopie (ZELMI) of the TU Berlin.

The Brunauer–Emmett–Teller (BET) surface area surface areas of the as-prepared material were determined on a Quantachrome Autosorb-1 apparatus. Nitrogen adsorption/desorption isotherms were measured at -196˚C after degassing the sample at 120˚C overnight. The BET surface areas (S_{BET}) were calculated from the adsorption data in a relative pressure ranging from 0.01 to 0.1.

Gas chromatography was used to calculate the Faradaic efficiency (FE) of oxygen evolution reaction (OER) that was performed in a closed (gas-tight) electrochemical cell. An Agilent 7890A gas chromatograph (GC) was used to determine the oxygen content in the headspace of the electrochemical cell. The GC was furnished with a carboxen-1000 column and a thermal conductivity detector (TCD). The carrier gas was argon (Ar).

X-ray photoelectron spectroscopy (XPS)

The XPS measurements were carried out on a Kratos Axis Ultra X-ray photoelectron spectrometer (Kratos Analytical Ltd., Manchester, U.K.) using an Al Kα monochromatic radiation source (1486.7 eV) with 90° takeoff angle (normal to analyzer). The vacuum pressure in the analyzing chamber was kept at 2×10^{-9} Torr. The XPS spectra were collected for C 1s, O 1s, Ni 2p, and Ge 3d levels with pass energy 20 eV and step 0.1 eV. The binding energies were calibrated relative to the C 1s peak energy position as 285.0 eV. Data analyses were carried out using Casa XPS (Casa Software Ltd.) and the Vision data processing program (Kratos Analytical Ltd.).

Quasi *In-situ* Raman spectroscopy

Raman spectra were recorded using the 458 nm emission of an Argon ion laser (Innova 70, Coherent) for excitation and a confocal Raman spectrometer (Lab Ram HR- 800 Jobin Yvon) equipped with a liquid-nitrogen cooled charge-coupled device (CCD) camera for data acquisition. The typical laser power at the sample ranged between 2-3 mW. The powdered NiGe was electrophoretically deposited on FTO and placed in a three-electrode cell and the chronoamperometry measurements were conducted by applying the desired potential for 24 in

1 M KOH electrolyte ((in analogy to the electrochemical measurements). For the ex-situ measurements, the measured films were taken out and stored under air and for the quasi *in-situ* experiments, the films were freeze-quenched after at an applied potential for 24 h using liquid N_2 under vigorous Ar gas flow and stored in liquid N_2. Raman measurements were conducted at 80 K using a Linkam Cryostage THMS600 cryostat and the films were probed at least three different parts that were found to be consistent with the attained peak positions.

Synthesis of the molecular precursor

Synthesis of complex 1

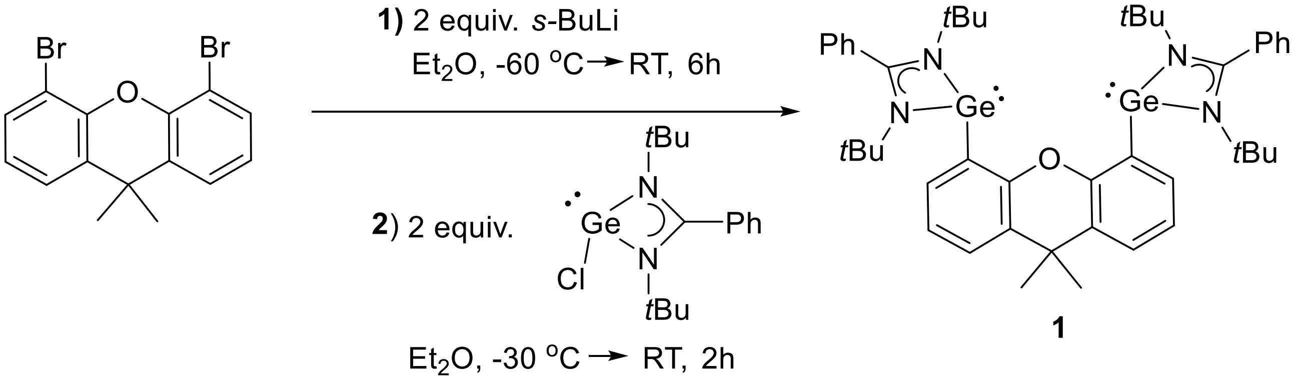

A *sec*-butyllithium (1.4 M in cyclohexene) solution (5.39 mL, 7.55 mmol) was added dropwise to a solution of 4,5-dibromo-9,9-dimethylxanthene (1.39 g, 3.78 mmol) in 100 mL Et_2O with stirring at -60 °C. The mixture was allowed to warm up to room temperature and stirred further for 6 h. Then the reaction mixture was cooled to -30 °C and chlorogermylene [PhC(N*t*Bu)$_2$]GeCl (2.56 g, 7.55 mmol) was added in portion into the mixture. The cooling bath was removed, and the reaction mixture was stirred at room temperature for 2 h. Volatiles were removed in vacuum and the residue was extracted with 100 mL n-hexane and 30 mL Et_2O. The combined filtrate was concentrated to 30 mL and compound **1** crystallized at -30 °C in 18h as yellow crystals (5.05 g, 82% isolated yield). Melting point: 116˚C (decomposed); ^{1}H NMR (400 MHz, C_6D_6, 298K): δ =1.12 (s, 36 H, NC(C*H*$_3$)$_3$), 1.65 (s, 6 H, C(C*H*$_3$)$_2$), 6.93–7.02 (m, 6 H, Ar(Ph)-*H*), 7.18–7.21 (m, 2 H, Ar(Ph)-*H*), 7.29–7.32 (m, 2 H, Ar(Ph)-*H*), 7.36 (dd, J_{HH} = 7.6 Hz, J_{HH} = 6.8 Hz, 2 H, Ar(Xant)-*H*), 7.41 (dd, J_{HH} = 7.6 Hz, J_{HH} = 1.8 Hz, 2 H, Ar(Xant)-*H*), 8.08 ppm (dd, J_{HH} = 6.8 Hz, J_{HH} = 1.8 Hz, 2 H, Ar(Xant)-*H*). ^{13}C{^{1}H} NMR (100.61 MHz, C_6D_6, 298K): δ = 31.7 (s, NC(*C*H$_3$)$_3$), 31.9 (s, C(*C*H$_3$)$_2$), 34.4 (s, *C*(CH$_3$)$_2$), 52.8 (s, N*C*(CH$_3$)$_3$), 122.6, 124.5, 127.8, 128.3, 129.1, 129.9, 130.0, 130.3, 134.5, 136.3, 139.6, 161.8 (s, Ar-*C*), 168.6 ppm (s, N*C*N). HR-APCI-MS (m/z): calcd for $[M+H]^+$ ($C_{45}H_{59}N_4OGe_2$): 817.31159; found: 817.31161. Elemental analysis (%) calcd for $C_{45}H_{58}N_4OGe_2$: C 66.22, H 7.16, N 6.86; found: C 66.08, H 7.22, N 6.59. IR (cm^{-1}): 3055 (w), 2964 (w), 2926 (w), 2900 (w), 2867 (w), 1469 (w), 1443 (m), 1428 (s), 1384 (s), 1359 (m), 1254 (w), 1200 (s), 1186 (m), 1158 (w), 1102 (w), 1089 (w), 1063 (m), 1019 (w), 925 (w), 871 (w), 793 (w), 781 (m), 757(w), 751(w), 743(w), 737(m), 708 (s).

Synthesis of Complex 2

To a Schlenk flask charged with **1** (0.63 g, 0.77 mmol) and Ni(cod)$_2$ (0.21 g, 0.77 mmol) was added 30 mL toluene at room temperature with stirring. An immediate color change from yellow to red was observed. After the mixture stirring at room temperature overnight, volatiles was removed in vacuum. The residue was extracted with n-hexane and the solution was concentrated to ca. 5 mL, from which compound **2** crystallized in 5 days at -30 ^{o}C as red crystals (0.66 g, 87% isolated yield). Melting point: 84 ˚C (decomposed); ^{1}H NMR (200 MHz, C_6D_6, 298K): δ = 1.13 (s, 36 H, NC(C*H*$_3$)$_3$), 1.61 (s, 6 H, C(C*H*$_3$)$_2$), 2.59 -2.74 (m, 4 H, C*H*$_2$), 2.92-2.31 (m, 4 H, C*H*$_2$), 4.95-5.06 (m, 4 H, =C*H*), 7.00-7.06 (m, 6 H, Ar(Ph)-*H*)l, 7.31–7.40 (m, 4 H, Ar(Xant)-*H*), 7.49–7.62 (m, 4 H, Ar(Ph)-*H*), 8.07–8.15 ppm (m, 2 H, Ar(Xant)-*H*). ^{13}C{^{1}H} NMR (50 MHz, C_6D_6, 298K): δ = 28.0 (s, C(*C*H$_3$)$_2$), 31.6 (s, NC(*C*H$_3$)$_3$), 32.0 (s, cod-*C*H$_2$), 37.0 (s, *C*(CH$_3$)$_2$), 53.4 (s, N*C*(CH$_3$)$_3$), 78.4 (s, cod-*C*H), 122.7, 125.7, 127.3, 127.4, 128.6, 128.7, 129.8, 130.2, 130.9, 136.8, 144.5, 157.1 (s, Ar-*C*), 165.2 ppm (s, N*C*N). HR-APCI-MS (m/z): calcd for $[M+H]^+$ ($C_{53}H_{71}N_4OGe_2Ni$): 983.34084; found: 983.33479. Elemental analysis (%) calcd for $C_{53}H_{70}N_4OGe_2Ni$: C 64.76, H 7.18, N 5.70; found: C 64.21, H 7.02, N 5.43. IR (cm^{-1}): 3053 (w), 2966 (w), 2925 (w), 2862 (w), 2807 (w), 1471 (w), 1432 (s), 1392 (s), 1359 (m), 1250 (w), 1202 (s), 1105 (w), 1063 (m), 1019 (w), 924 (w), 876 (w), 816(w), 789(m), 761(w), 737(m), 705 (s), 675 (m).

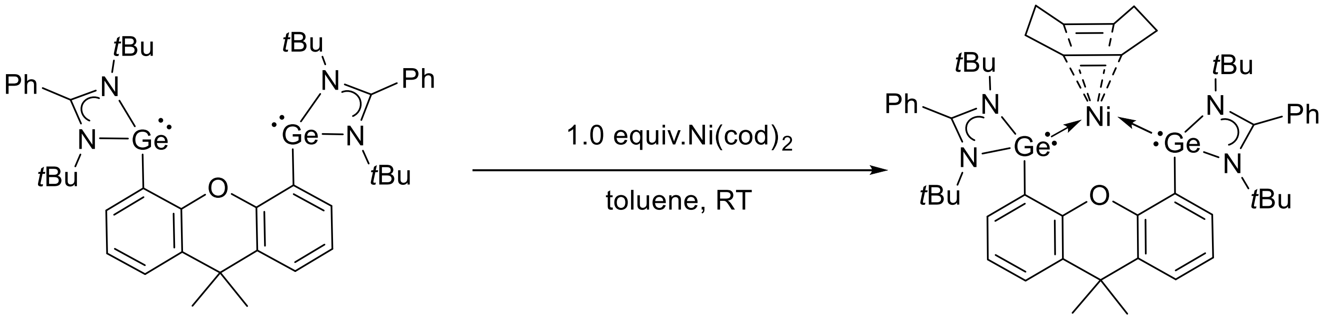

1 **2**

Single-Crystal X-ray Structure Determination

The crystal was mounted on a glass capillary in per-fluorinated oil and measured in a cold N_2 flow. The data of **1-2** were collected on an Oxford Diffraction Supernova, Single source at the offset, Atlas at 150 K (Cu- Kα-radiation, λ = 1.5418 Å). The structure was solved by direct method and refined on F^2 with the SHELX-97 software package.[3] The positions of the H atoms were calculated and considered isotropically according to a riding model. In the molecular structure of compound 2, n-hexane as solvent molecules are strongly disordered and treated using the SQUEEZE routine in PLATON. CCDC 2016767 (compound **1**) and CCDC 2016768 (compound **2**) contain the supplementary crystallographic data for this paper. These data can be obtained free of charge by contacting The Cambridge Crystallographic Data Centre, 12, Union Road, Cambridge CB2 1EZ, UK; Fax: +44 1223 336033.

Synthesis of the catalysts

Synthesis of intermetallic NiGe

To a three-necked round bottom Schlenk flask fitted with a temperature sensor and a condenser, 25 mL oleylamine (Sigma-Aldrich) was added. The solvent was previously degassed by a 3-cycle freeze-pump method and stored with molecular sieves (3 Å). The complete set up was degassed using a vacuum pump followed by nitrogen refill (three times) and then the flask was heated to 250 °C. First of all, the $C_{47}H_{64}Ge_2N_4NiO$ precursor (983.1 g/mol; 150 mg) was dissolved in 5 mL of dry oleylamine in a separate flask. This solution was then transferred to the three-necked flask at 250 °C by injection under inert conditions. The reaction temperature was maintained at 250 °C for 1 h and then the mixture was allowed to cool down naturally to room temperature. The reaction mixture was transferred into a centrifuge tube and centrifuged by the addition of 20 mL ethanol at 9000 rpm to produce a black solid product. Finally, the mixture was placed in an ultrasonic bath for 15 min to remove any excess ligand and oleylamine. Sonication and centrifugation cycles were repeated 4 additional times. The solid product was washed with acetone (1×50 mL) and dried overnight at 60 °C and used further for characterizations. The attained yield was 75 % (with <2% carbon).

Synthesis of $Ni(OH)_2$[4]

291 mg of $Ni(NO_3)_2.6H_2O$ was dissolved in 10 mL of deionized water and to this solution, 15 mL of NaOH (0.1 M) was added dropwise and stirred continuously for 30min. A bright green solid product was separated, which was then washed and centrifuged thrice with deionized water (3×50 mL), once with acetone (1×50 mL), before drying at 60 °C overnight in air.

Synthesis of NiOOH[5]

The as-prepared $Ni(OH)_2$ was dispersed in 30 mL 4 M KOH solution which was then heated slowly up to 45 °C. An excess of $K_2S_2O_8$ was then added and maintained at the same temperature for 18 h until the complete change of color to black was obtained. The black solid was washed and centrifuged 3 times with deionized water three times water (3×50 mL), once with acetone (1×50 mL), then dried at 120 °C overnight in air.

Synthesis of FeOOH[5]

500 mg of $FeSO_4.7H_2O$ (Merck Millipore) (1.80 mmol) was dissolved on 10 mL of deionized water. 4 mL of 1 M NaOH (4 mmol) was added dropwise and stirred for 30 min at room temperature. The dark brown suspension was then heated gently up to 45 °C and 2 mL of 30% H_2O_2 solution was then added dropwise. The resulted orange suspension was allowed to stir at the same temperature for another 18 h. The final brown precipitate was centrifuged out and washed with deionized water three times, then dried at 60 °C overnight in air

Synthesis of $FeNi_2O_4$[6]

1.988 g $FeCl_2{\cdot}4H_2O$ and 4.977 g $Ni(OAc)_2.4H_2O$ were dissolved in a minimum amount of ethanol and stirred for 30 minutes. The solvent was then evaporated at room temperature to obtain the dried powder that was calcinated overnight at 600 °C in oxygen to yield $FeNi_2O_4$.

Synthesis of $Co(OH)_2$[5]

50 mL of 0.1 mol L^{-1} NaOH solution was added dropwise into 80 mL of 0.05 mol L^{-1} $Co(NO_3)_3$ solution. The solution was stirred and maintained at 45 °C for two hours which forms a pink precipitate, which was then washed with deionized water (3×50 mL) and dried in air at 60 ºC.

Synthesis of CoOOH[5]

The as-synthesized $Co(OH)_2$ was dispersed in 30 mL 4 M KOH solution which was then slowly heated up to 45 ºC. To this, 2 mL of 30% H_2O_2 solution was then added dropwise and was kept at the same temperature for 18 h. The final brown precipitate was filtered and washed with deionized water three times (3×50 mL), then dried at 60 °C overnight in air.

Electrophoretic deposition (EPD) on substrates

The investigated materials were deposited on both, NF and FTO, electrophoretically, by applying a potential difference of 10 V in a mixture of iodine and acetone on a 1 × 1 cm^2 area. The detailed mechanism involving electrophoretic deposition has been described elsewhere.[7] The electric charge on the catalyst in acetone is insufficient for EPD as very small amounts of free ions exist in acetone, and therefore, large potentials are required for EPD.[7] When iodine is used as the dispersant, it can react with acetone through the keto-enol tautomerism to produce protons as per the following equation.

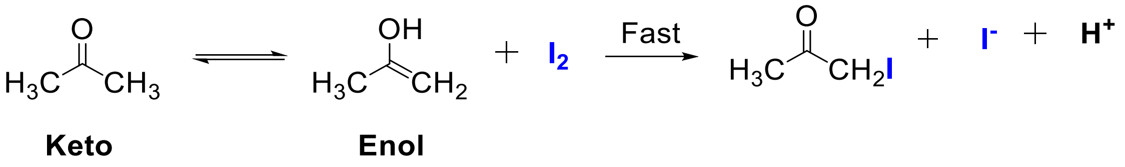

Hence, the released protons are adsorbed on the surface of the suspended particles by making them positively charged. The applied electric field induces the positively charged particles to migrate towards and deposit on the cathode.[8] For the typical deposition protocol, 30 mg of the catalyst powder was suspended in 10 ml acetone, and 3 mg of iodine was then added. This solution was agitated in an ultrasonic bath for 30 min. Before EPD, the empty electrodes were weighed using an analytical balance, and the weights were noted. The EPD was conducted at various potentials different time intervals, and the thin uniform films were only achieved by applying a potential at -10 V between 60-120 s with stirring the solution continuously at room temperature. After each EPD, the increase in weight of the electrodes was monitored carefully. Depending upon the catalyst, the loading was optimized with EPD time and loading on each NF and FTO was ~1 $mgcm^{-2}$ and ~0.4 $mgcm^{-2}$, respectively. The mass loading was reproducible within the margins of an experimental error (±0.08 mg).

Electrochemical measurements

A typical electrocatalytic run was carried out in a standard three-electrode (working, counter, and reference) electrochemical cell in 1 M aqueous KOH with a potentiostat (SP-200, BioLogic Science Instruments) controlled by the EC-Lab v10.20 software package. The electrodes (NF/FTO) with deposited catalysts served as the working electrodes, Pt wire (0.5

mm diameter × 230 mm length; A-002234, BioLogic) as a counter and Hg/HgO as the reference electrode (CH Instruments, Inc.). Cyclic voltammetry (CV) and linear sweep voltammetry (LSV) were carried out with an applied *iR* compensation of 85%. The potentials presented in this work were referenced to the reversible hydrogen electrode (RHE) through calibration and in 1 M aqueous KOH, E(RHE) = E(Hg/HgO) + 0.098 V + (0.059 × pH) V where the used pH value was 13.8. The chronoamperometric (CA) measurements were performed in 1 M aqueous KOH at selected constant potentials with respect to RHE. Under CA conditions, the films were mechanically stable and have superior adhesion to the substrate. The OER CP experiments at elevated temperatures were carried out in a temperature controlled closed cell. Approximately 6 cm of the 12 cm long Hg/HgO reference electrode was inside the heated electrolyte. The rest of it remained outside at room temperature. The temperature in the reference electrode (T_{ref}) was checked regularly and after 1 h, it remained stable at 44 °C. To adjust the reference electrode potential ($E_{Hg/HgO}$), the following equation was used which originates from a linear regression of the data points at 298.15 and 313.15 K:[9]

$$E_{Hg/HgO} = 99.0 \text{ mV} - 1.16 \times (T_{ref} - 298.15 \text{ K}) \text{ mV K}^{-1}$$

The Tafel slope was calculated according to Tafel equation $\eta = b\log j + a$, where η is overpotential (V), j is the current density (mAcm^{-2}), and b is the Tafel slope (mVdec^{-1}).[10]

The electrochemically active surface area (ECSA) of the catalyst films was obtained by determining their double layer capacitances (C_{dl}) from the CV (cycled between 0.86 and 0.96 V vs. RHE) at a potential range, where no apparent faradaic process occurred.[11] The anodic charging currents measured at 0.91 V vs. RHE were plotted as a function of the scan rate and from the slope, and the double layer capacitance C_{dl} was attained. The ECSA of the catalysts were then calculated using the equation

$$\text{ECSA} = C_{dl} / C_s$$

Where C_s can be defined as the specific capacitance of the material per unit area under identical electrolyte conditions. As C_{dl} is expected to be linearly proportional to the effective active surface area, we have normalized the current values to their C_{dl}.[12]

The electrochemical impedance spectroscopy (EIS) of the catalysts films was recorded at 1.5 V (NF) and 1.55 V (FTO) vs. RHE to achieve the Nyquist plots. The amplitude of the sinusoidal wave was examined in a frequency range of 100 kHz to 1 mHz. All impedance spectra were fitted using an equivalent RC circuit model. In the equivalent circuit, R_s represents the uncompensated solution resistance, R_{ct} is the charge transfer resistance and CPE is the constant phase element. The charge transfer resistance (R_{ct}) was then obtained from the diameter of the semicircle in the Nyquist plots.[11,13] All electrocatalytic measurements were performed at least thrice to obtain reliable data.

Table S1. Crystal data and structure refinement for **1**

Crystal Data and Refinement for 1	
**Empirical formula**	$C_{51} H_{72} Ge_2 N_4 O$
**Formula weight**	902.30
**Temperature**	150(2) K
**Wavelength**	1.54184 Å
**Crystal system**	Monoclinic
**Space group**	$P2_1/n$
**Unit cell dimensions**	a = 13.89110(10) Å, α = 90°
	b = 25.5240(2) Å, β = 99.1020(10)°.
	c = 14.01230(10) Å, γ = 90°.
**Volume**	4905.59(6) Å^3
Z	4
**Density (calculated)**	1.222 Mg/m^3
**Absorption coefficient**	1.795 mm^{-1}
**F(000)**	1912
**Crystal size**	0.590 x 0.450 x 0.340 mm^3
**Theta range for data collection**	3.463 to 67.497°
**Index ranges**	-16<=*h*<=16, -30<=*k*<=21, -16<=*l*<=16
**Reflections collected**	33785
**Independent reflections**	8843 [R(int) = 0.0303]
**Completeness to theta = 67.497°**	99.9 %
**Absorption correction**	Semi-empirical from equivalents
**Max. and min. transmission**	1.00000 and 0.51259
**Refinement method**	Full-matrix least-squares on F^2
**Data / restraints / parameters**	8843 / 0 / 539
**Goodness-of-fit on F^2**	1.027
**Final R indices [I>2sigma(I)]**	R1 = 0.0389, wR2 = 0.1006
**R indices (all data)**	R1 = 0.0418, wR2 = 0.1034
**Extinction coefficient**	N/A
**Largest diff. peak and hole**	0.894 and -0.989 e.Å^{-3}

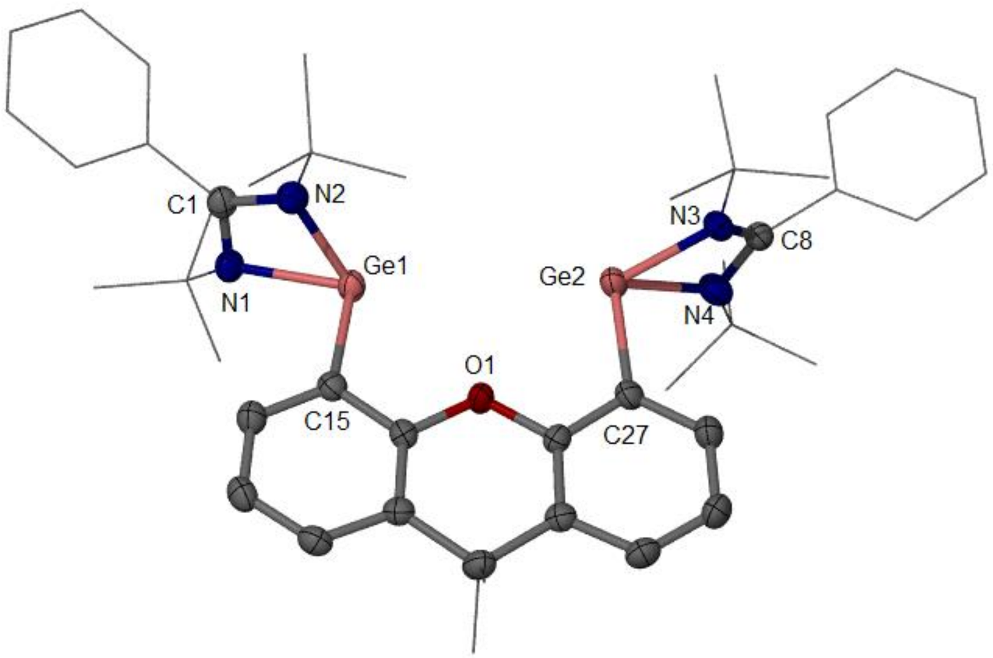

Figure S1. Molecular structure of compound **1**. Thermal ellipsoids are drawn at a 30% probability level. H atoms are omitted for clarity.

Table S2. Selected interatomic distances [Å] and angles [°] for compound **1**.

Bond distances	[Å]	Bond angels	[°]
Ge(1)-N(2)	2.018(2)	N(2)-Ge(1)-N(1)	64.69(7)
Ge(1)-N(1)	2.026(2)	C(1)-N(1)-Ge(1)	92.61(13)
Ge(1)-C(15	2.030(2)	N(2)-C(1)-N(1)	108.73(19)
N(1)-C(1)	1.336(3)	N(4)-Ge(2)-N(3)	64.64(8)
C(1)-N(2)	1.327(3)	N(4)-Ge(2)-C(27)	97.26(8)
Ge(2)-N(4**)**	2.006(2)	N(3)-Ge(2)-C(27)	96.27(8)
Ge(2)-N(3**)**	2.013(2)	C(1)-N(2)-Ge(1)	93.28(14)
Ge(2)-C(27)	2.021(2)	C(8)-N(3)-Ge(2)	92.59(13)
N(3)-C(8)	1.328(3)	C(8)-N(4)-Ge(2)	92.83(14)
N(4)-C(8)	1.331(3)	N(3)-C(8)-N(4)	107.87(19)

Table S3. Crystal data and structure refinement for **2.**

Crystal Data and Refinement for 2	
**Identification code**	D:~2_sq
**Empirical formula**	$C_{53} H_{70} Ge_2 N_4 Ni O$
**Formula weight**	983.02
**Temperature**	150(2) K
**Wavelength**	1.54184 Å
**Crystal system**	Trigonal
**Space group**	*R*-3
**Unit cell dimensions**	*a* = 27.0416(11) Å, α = 90°.
	b = 27.0416(11) Å, β = 90°.
	c = 46.584(2) Å, γ = 120°.
**Volume**	29501(3) $Å^3$
Z	18
**Density (calculated)**	0.996 Mg/m^3
**Absorption coefficient**	1.644 mm^{-1}
**F(000)**	9288
**Crystal size**	0.380 x 0.240 x 0.210 mm^3
**Theta range for data collection**	2.676 to 67.493°
**Index ranges**	-32<=*h*<=32, -32<=*k*<=32, -55<=*l*<=55
**Reflections collected**	66074
**Independent reflections**	11805 [R(int) = 0.0729]
**Completeness to theta = 67.497°**	99.8 %
**Absorption correction**	Semi-empirical from equivalents
**Max. and min. transmission**	1.00000 and 0.20568
**Refinement method**	Full-matrix least-squares on F^2
**Data / restraints / parameters**	11805 / 60 / 608
**Goodness-of-fit on F^2**	1.045
**Final R indices [I>2sigma(I)]**	R1 = 0.0795, wR2 = 0.1842
**R indices (all data)**	R1 = 0.0919, wR2 = 0.1979
**Extinction coefficient**	N/A
**Largest diff. peak and hole**	1.159 and -0.934 e.$Å^{-3}$

Table S4. Selected interatomic distances [Å] and angles [°] for compound **2.**

Bond distances	[Å]	Bond angels	[°]
**Ge(1)-N(1)**	2.011(6)	**N(1)-Ge(1)-N(2)**	65.0(2)
**Ge(1)-N(2)**	2.051(5)	**N(1)-Ge(1)-C(3)**	92.0(2)
**Ge(1)-C(3)**	2.058(6)	**N(2)-Ge(1)-C(3)**	94.2(2)
**Ge(1)-Ni(1)**	2.315(4)	**N(1)-Ge(1)-Ni(1)**	120.1(2)
**Ge(1)-C(1)**	2.466(6)	**N(2)-Ge(1)-Ni(1)**	125.0(2)
**Ni(1)-C(40)**	2.063(7)	**C(3)-Ge(1)-Ni(1)**	136.3(2)
**Ni(1)-C(36)**	2.070(7)	**N(1)-Ge(1)-C(1)**	32.24(18)
**Ni(1)-C(37)**	2.080(7)	**N(2)-Ge(1)-C(1)**	32.78(19)
**Ni(1)-C(41)**	2.081(7)	**C(3)-Ge(1)-C(1)**	94.3(2)
**Ni(1)-Ge(2)**	2.311(4)	**Ni(1)-Ge(1)-C(1)**	128.9(2)
**N(1)-C(1)**	1.318(7)	**Ge(2)-Ni(1)-Ge(1)**	101.6(2)
**C(1)-N(2)**	1.335(7)	**C(1)-N(1)-Ge(1)**	93.3(4)
**Ge(2)-C(10)**	2.008(6)	**N(1)-C(1)-N(2)**	110.7(5)
**Ge(2)-N(3)**	2.049(4)	**N(1)-C(1)-Ge(1)**	54.5(3)
**Ge(2)-N(4)**	2.118(6)	**N(2)-C(1)-Ge(1)**	56.2(3)
**C(2)-N(3)**	1.330(7)	**C(10)-Ge(2)-N(3)**	94.9(2)
**C(2)-N(4)**	1.332(7)	**C(10)-Ge(2)-N(4)**	90.1(3)
**C(36)-C(37)**	1.372(9)	**N(3)-Ge(2)-N(4)**	63.23(19)
**C(40)-C(41)**	1.401(8)	**C(10)-Ge(2)-Ni(1)**	138.0(2)
		**N(3)-Ge(2)-Ni(1)**	124.50(19)
		**N(4)-Ge(2)-Ni(1)**	118.8(2)
		**C(1)-N(2)-Ge(1)**	91.0(3)
		**N(3)-C(2)-N(4)**	110.3(4)

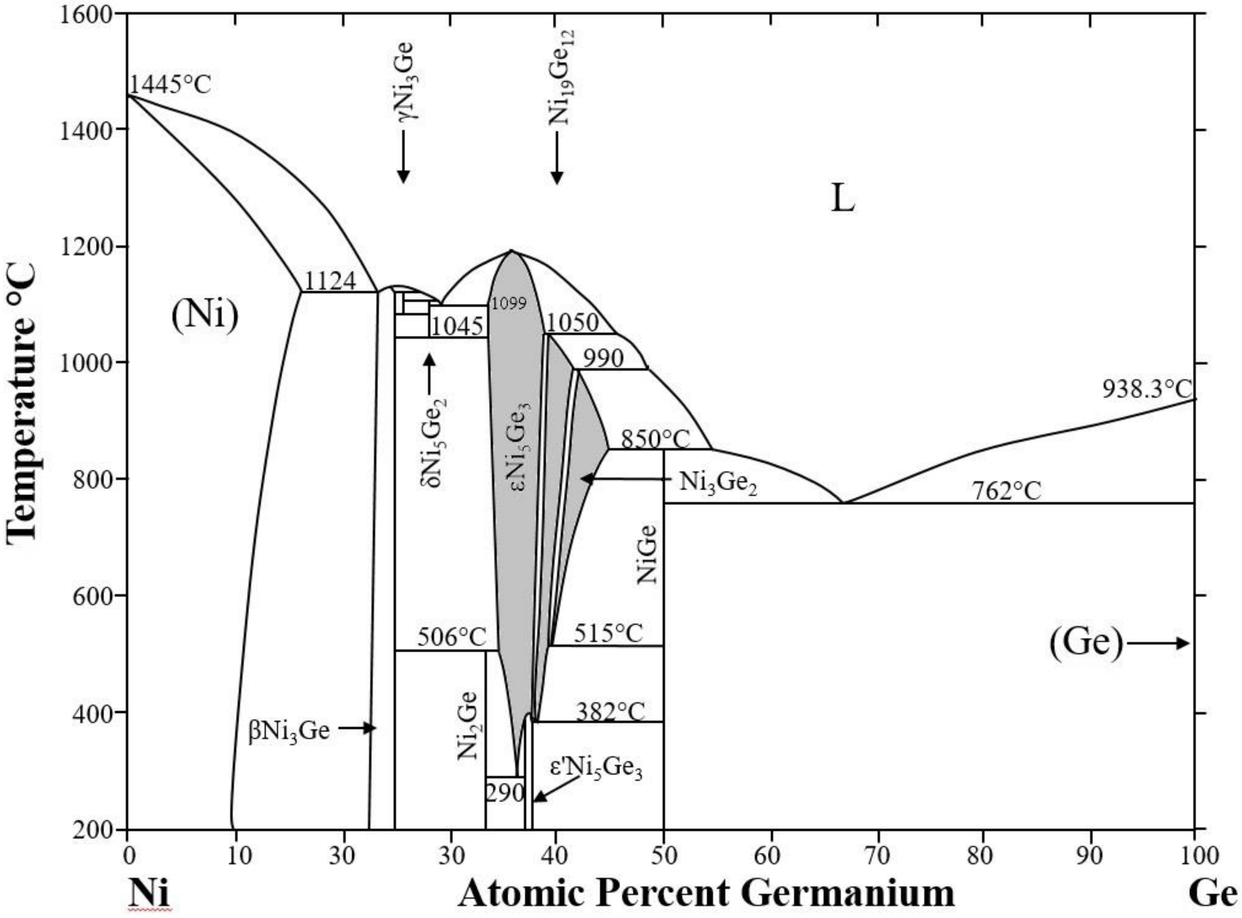

Figure S2. Equilibrium phase diagram of Ni-Ge binary system.[14]

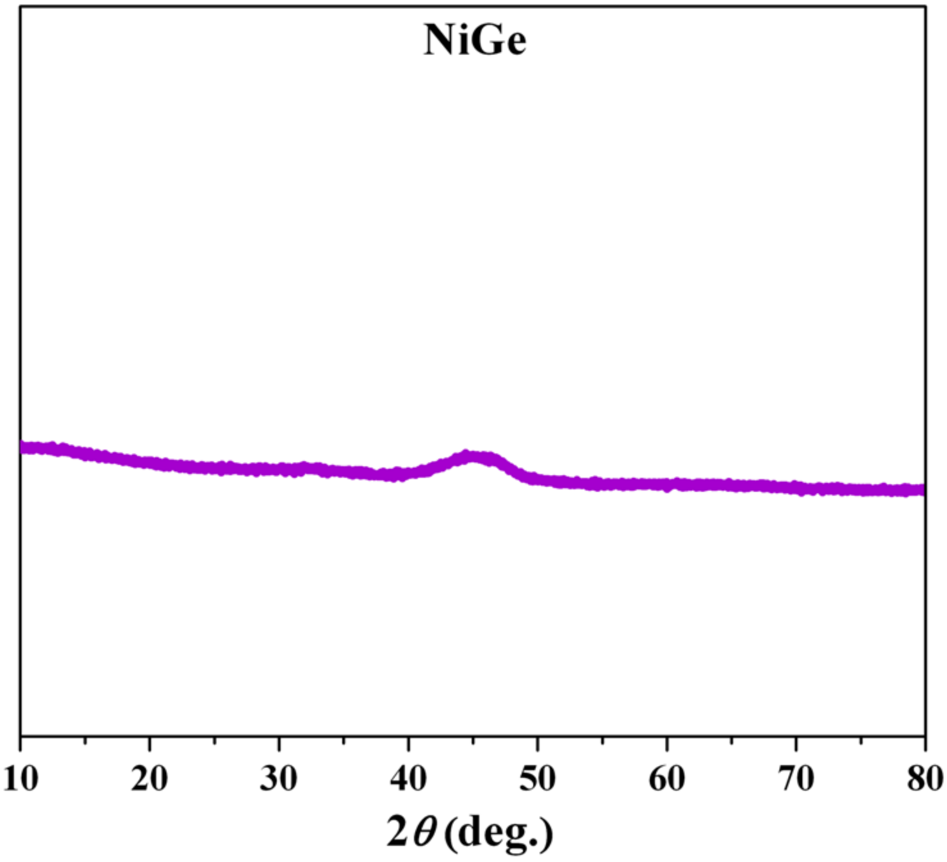

Figure S3. The PXRD pattern of (a) as-synthesized NiGe (JCPDS 74-247) prepared by hot-injection of the molecular precursor. The obtained NiGe material produces very small crystallites resulting in weak diffraction patterns (amorphous-like nature). The broad peak at 2 theta value of 45° can be indexed to the most intense (121) plane of NiGe. The phase confirmation and elemental composition of NiGe were further identified by HRTEM, SAED, EDX, ICP-AES, and XPS studies (see Table S5). The elemental analysis showed that the carbon residue is below 2%.

Table S5. The determination of nickel and germanium ratio in NiGe powder, as deposited on FTO and after OER CV and OER CA. The ratios were obtained by ICP-AES, EDX, and XPS analysis. Each experiment was conducted thrice independently, and the average value is presented.

	Ni:Ge (Theo.)	Ni:Ge (EDX)	Ni:Ge (ICP-AES)	Ni:Ge (XPS)
NiGe Powder	1:1	1:1.04±0.07	1:1.01±0.02	~1:1
NiGe deposited	1:1	1:1.03±0.05	-	~1:1
NiGe OER CV	-	1:0.10±0.04	1.0.08±0.03	~1:0.1
NiGe OER CA	-	1:0.08±0.05	1:0.05±0.02	~1:0.09

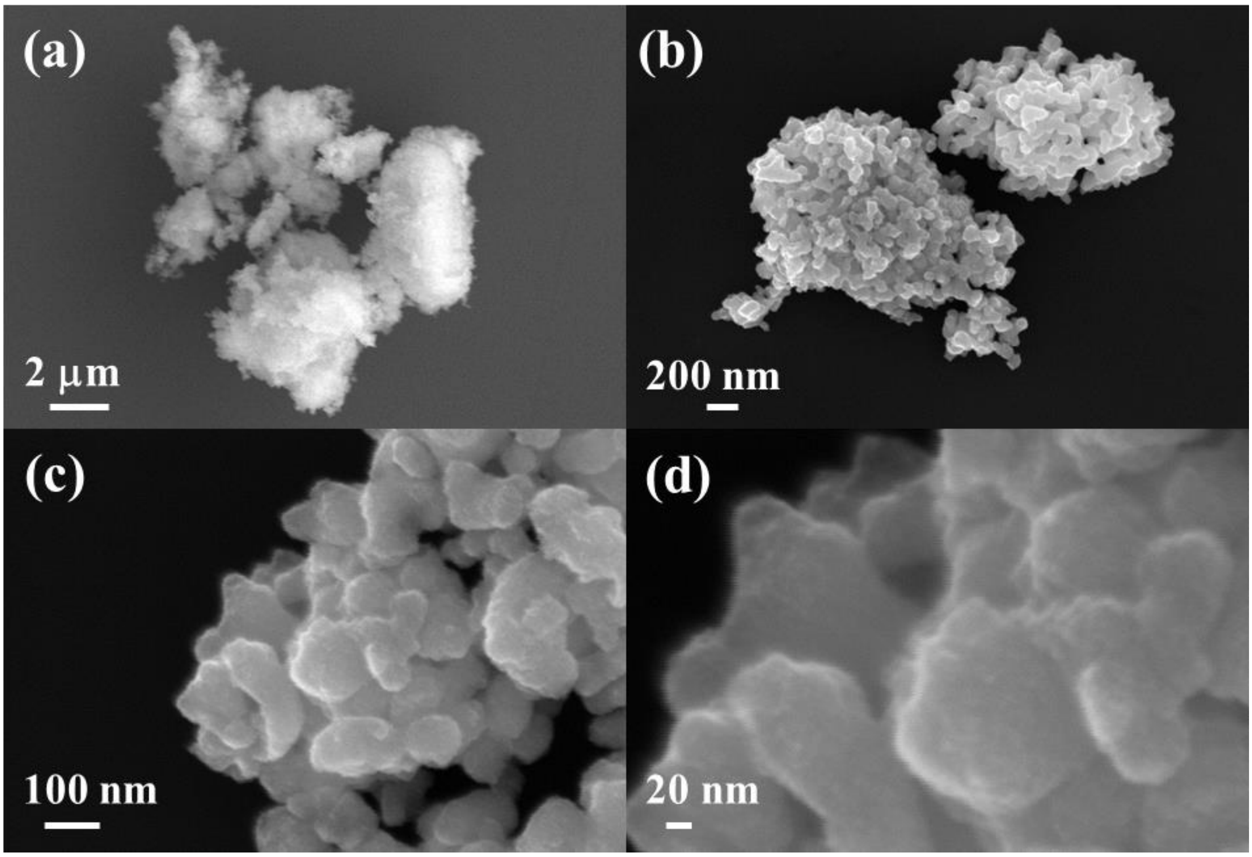

Figure S4. SEM images (a-d) of as-synthesized NiGe powder at different magnifications. The morphology of NiGe showed agglomeration of small nanoparticles into a larger cluster.

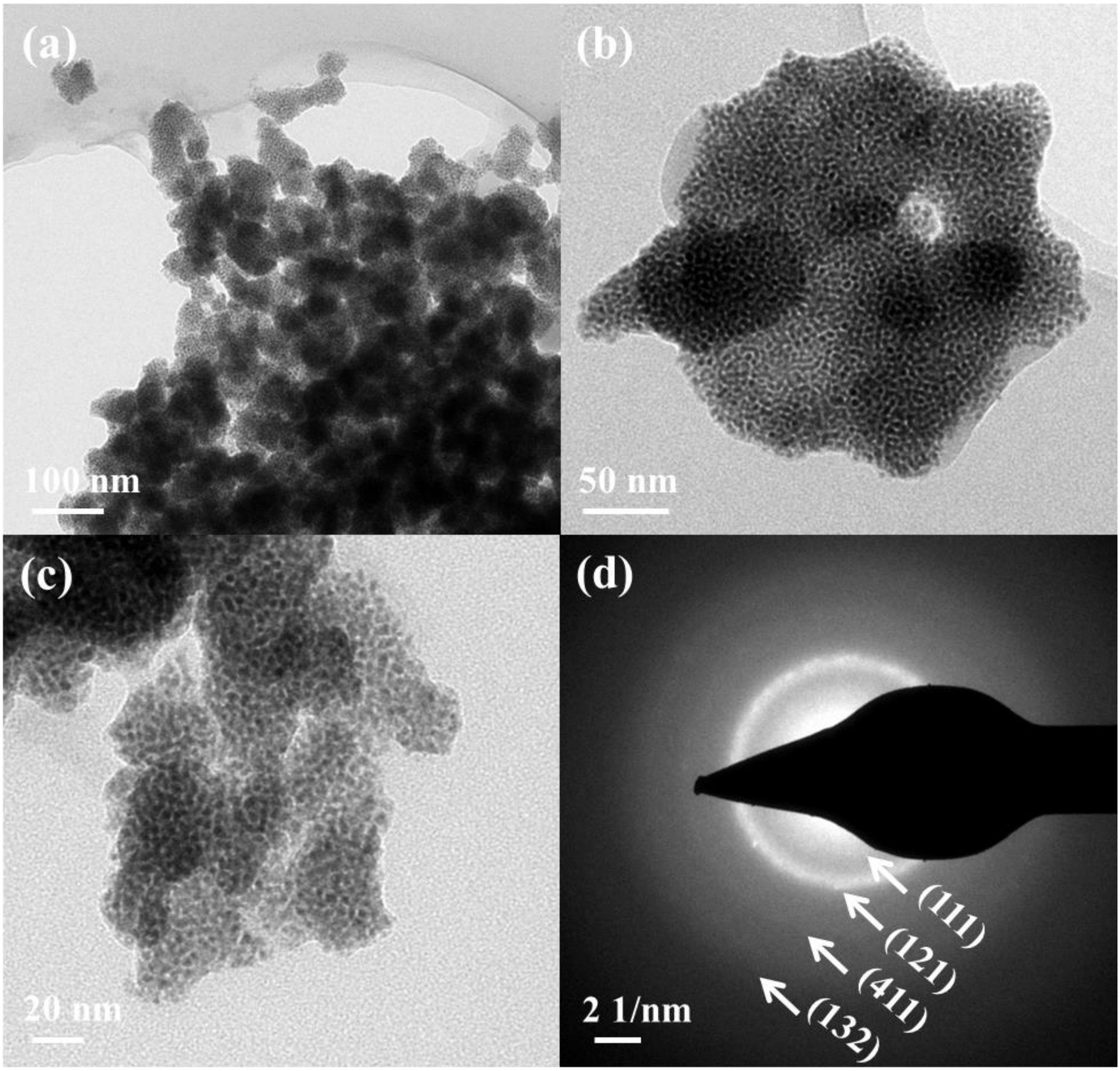

Figure S5. The TEM (a, b) and High-resolution TEM (HR-TEM) images (c) of as-synthesized NiGe powder revealing the presence of ultra-small nanoparticles of ~2 nm that are assembled together forming larger clusters. The selected area diffraction (SAED) pattern (d) displayed weak diffractions rings corresponding to the crystallographic planes (111), (121) (411) and (132) at d = 0.26, 0.20, 0.13 and 0.12 nm which are also consistent with the PXRD (JCPDS 7-297) pattern confirming the phase purity of the product.[15]

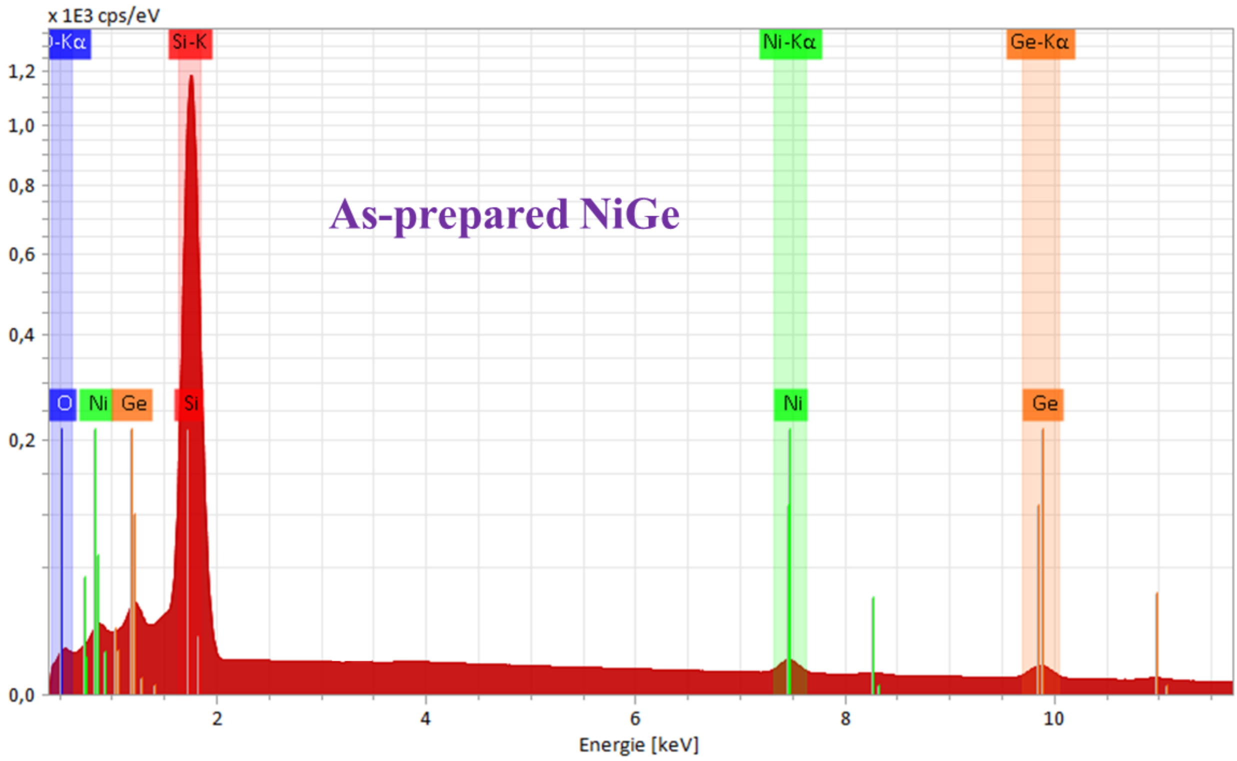

Figure S6. The EDX mapping spectrum of as-prepared NiGe powder. The obtained oxygen content was less than 1%.

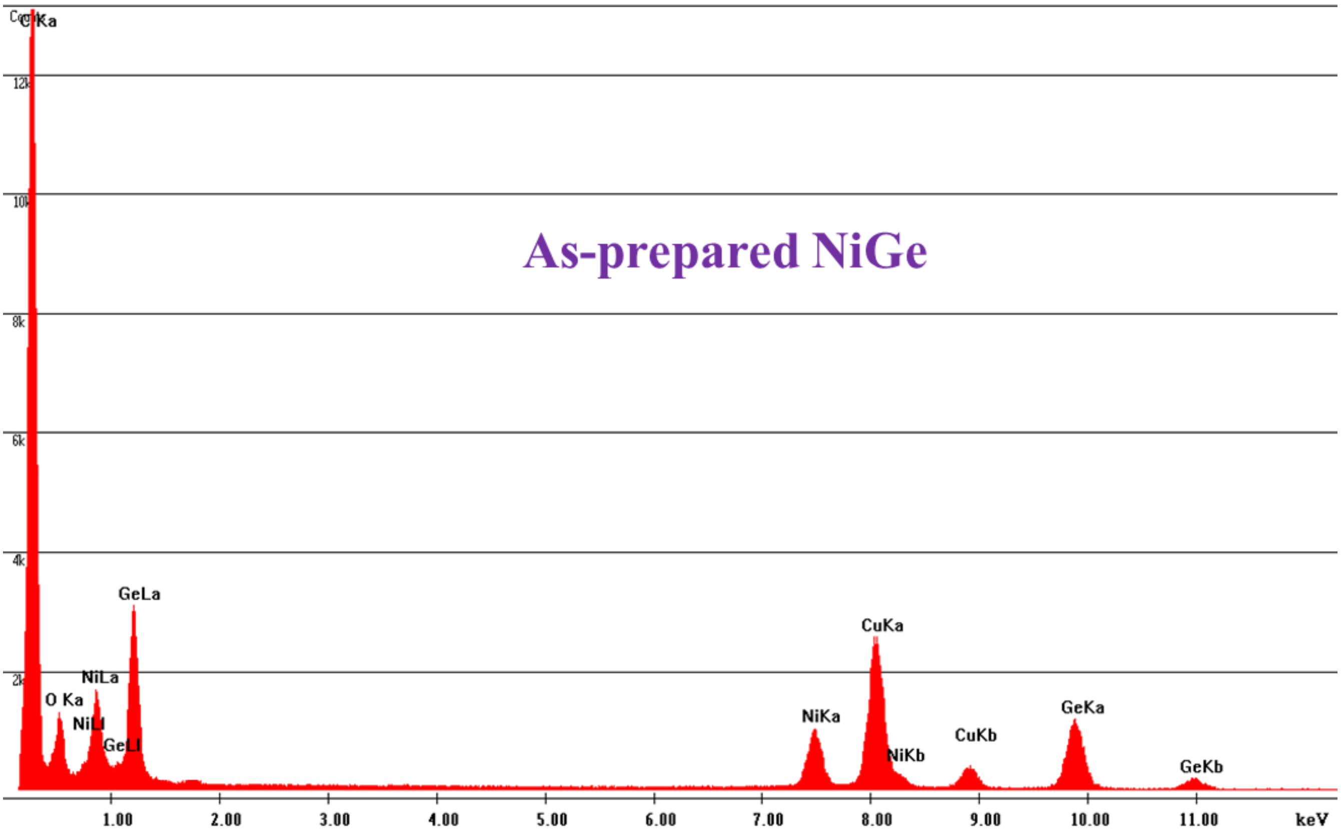

Figure S7. EDX analysis of as-synthesized NiGe powder confirming the presence of Ni and Ge. The peaks for copper and carbon can be unambiguously correlated to the TEM grid (carbon film on 300 mesh Cu-grid).

[100]

[010]

[001]

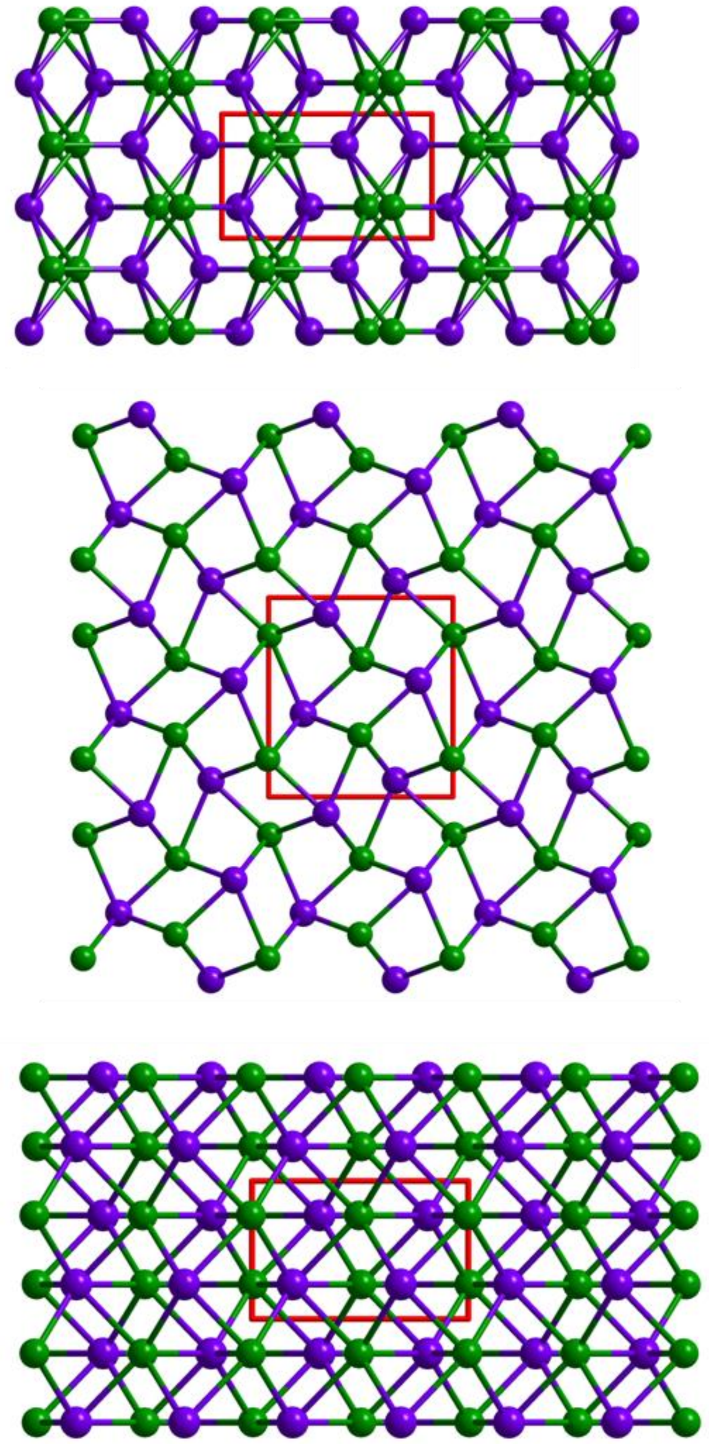

Figure S8. The crystal structure of NiGe (Ni: green; Ge: purple) on crystallographic [100], [010] and [001] direction.[15-16] The NiGe crystallizes in the orthorhombic system (isotypic to MnP) with the space group *Pnam* (No. 62) with the lattice parameters a = 5.811 Å, b = 5.381 Å, c = 3.438 Å, and Z= 4 V = 107.19 Å^3. Both the Ni and the Ge atoms occupy the 4c (x, 1/4, z) crystallographic positions. The unit cell of NiGe consists of four Ni and four Ge atoms. The Ni atoms have six first Ge neighbors forming a distorted octahedron, and the Ge atoms have six first neighbors (Ni atoms) forming a distorted trigonal prism.[15-16]

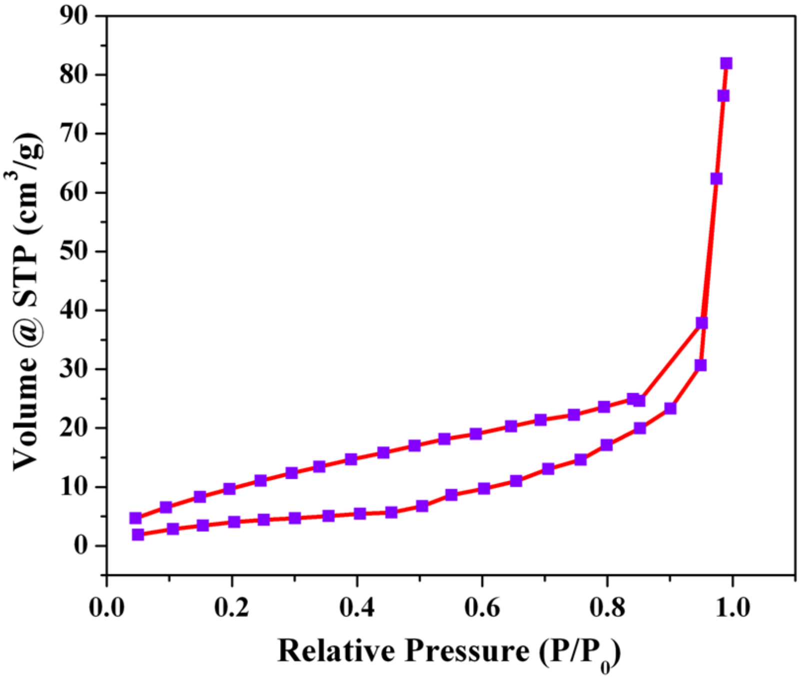

Figure S9. The N_2 adsorption-desorption isotherm of as-synthesized NiGe powder. The BET value of NiGe was achieved to be 17.3±0.9 m^2g^{-1}

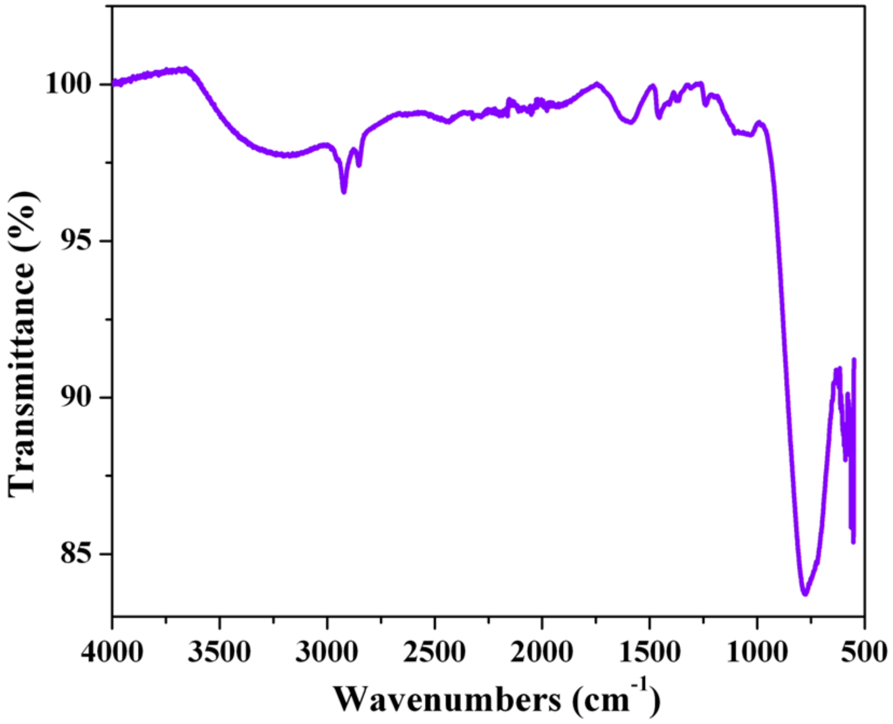

Figure S10. The FT-IR spectra of as-synthesized NiGe powder. The band between 500-1000 cm^{-1} could be ascribed to vibrations for Ni–Ge while a broad band between 3200-3600 cm^{-1} is due to hydroxylation of the intermetallics.[17]

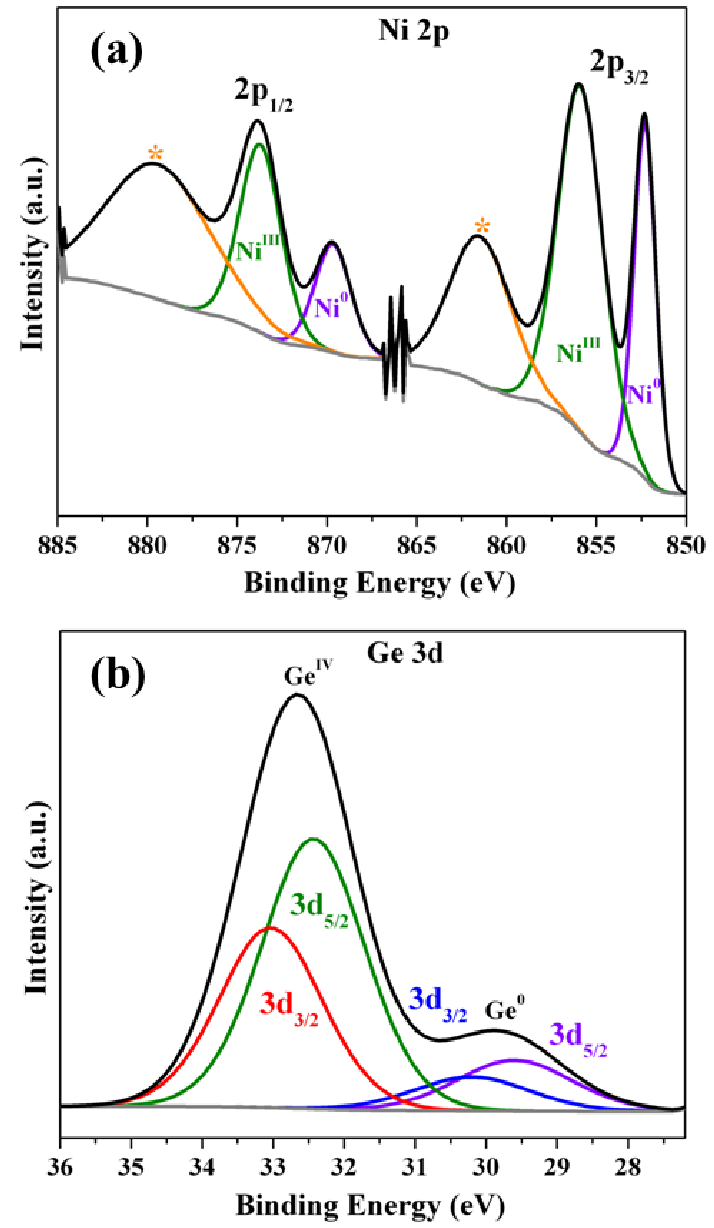

Figure S11. The high-resolution deconvoluted (a) Ni 2p and (b) Ge 3d XPS spectra of as-synthesized NiGe powder. Both Ni $2p_{3/2}$ and Ni $2p_{1/2}$ spectrum exhibited sharp peaks at the binding energy of 852.4 and 869.7 eV corresponding to Ni^0 of NiGe[18] whereas the other two peaks at 856 and 873.8 eV are attributed to the presence of Ni^{III} in the structure, which is due to the surface passivation of the intermetallic compound and is in good agreement with the literature reported intermetallic materials.[19] In addition, two satellite peaks, due to multi-electron excitation were also observed within the deconvoluted regions of Ni $2p_{3/2}$ and Ni $2p_{1/2}$ at 861.6 and 879.7 eV, respectively, that are characteristics of materials containing Ni.[19c] In the case of Ge 3d, the binding energy of 29.5 and 30.2 eV was attained for Ge $2p_{5/2}$ and $2p_{3/2}$, respectively, which is very similar to the binding energy of elemental Ge confirming the oxidation of Ge^0 in the NiGe material.[18] In addition to the Ge^0, major peaks at 32.4 and 33.1 eV corresponding to Ge $2p_{5/2}$ and $2p_{3/2}$ were obtained and are comparable to oxidized Ge^{IV} species from the surface passivation.[20] The surface atomic Ni:Ge composition of as-prepared material was ~1:1.

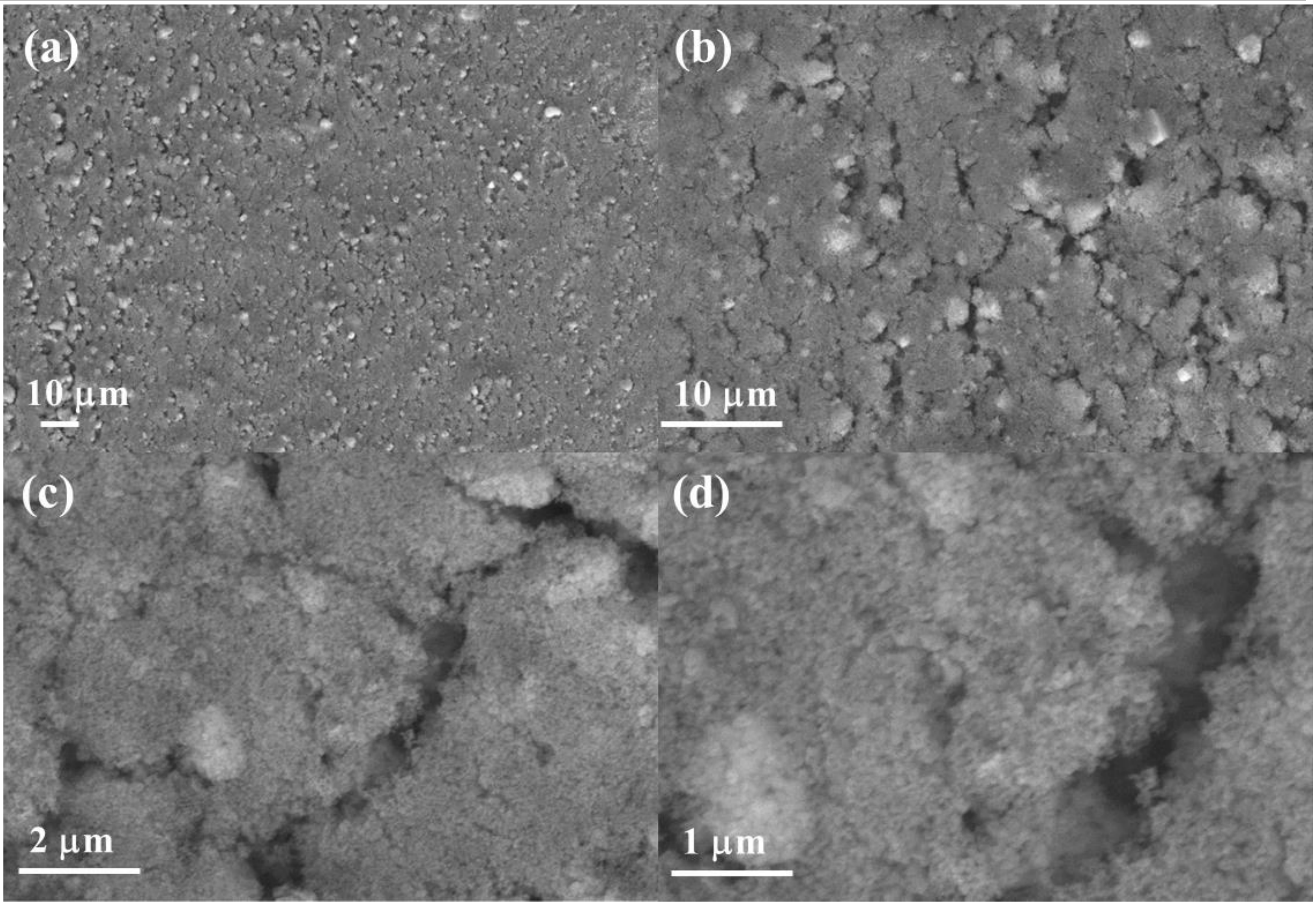

Figure S12. The SEM image (a-d) of the electrophoretically deposited NiGe film on FTO substrate (NiGe/FTO) at different magnifications showing NiGe nanoparticles forming and homogenous film over the FTO substrate electrode.

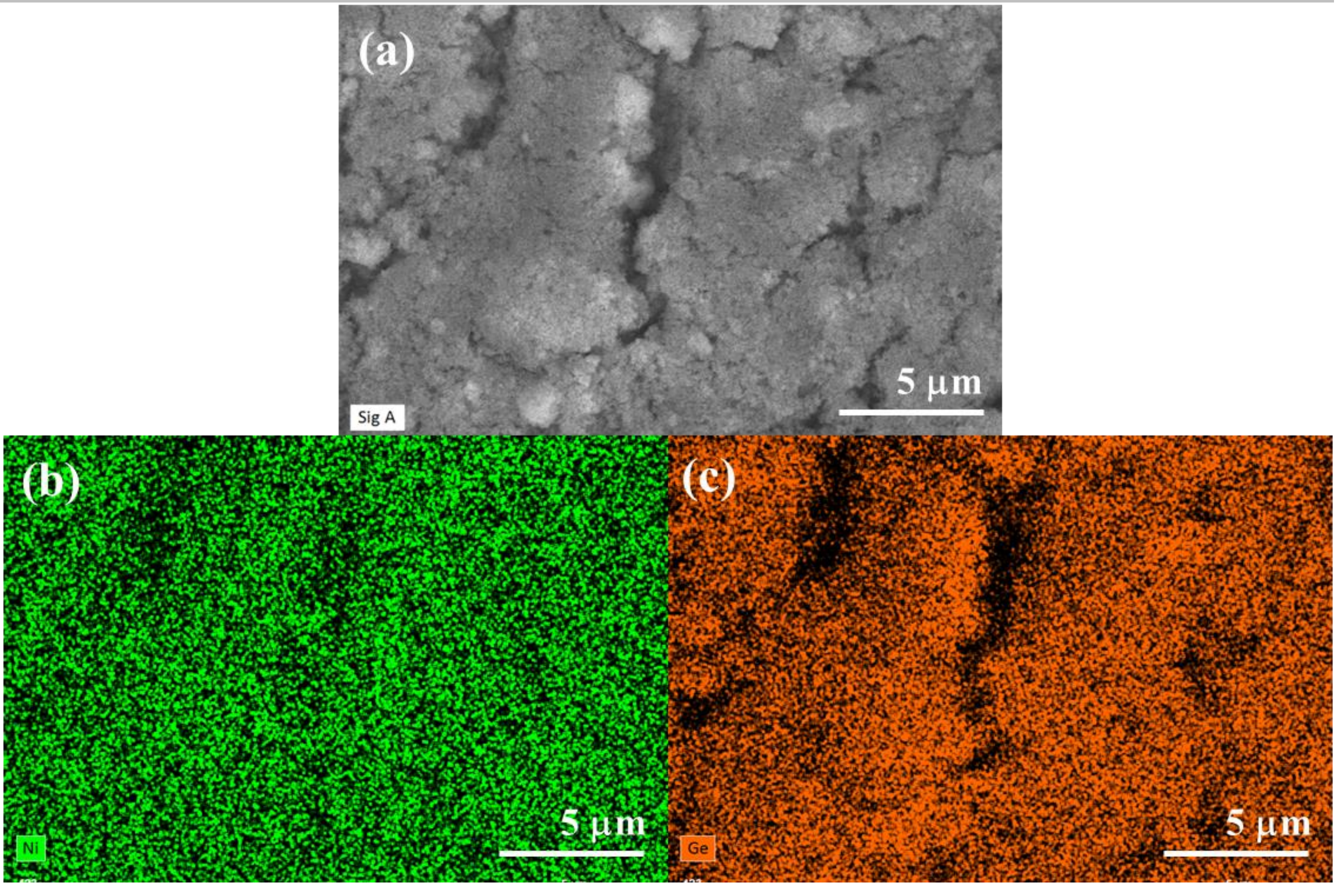

Figure S13. The SEM image (a) and the EDX mapping (b-c), which was carried out on the electrophoretically deposited NiGe/FTO film showing a homogenous distribution of nickel and germanium within the structure without any oxygen content (<1%).

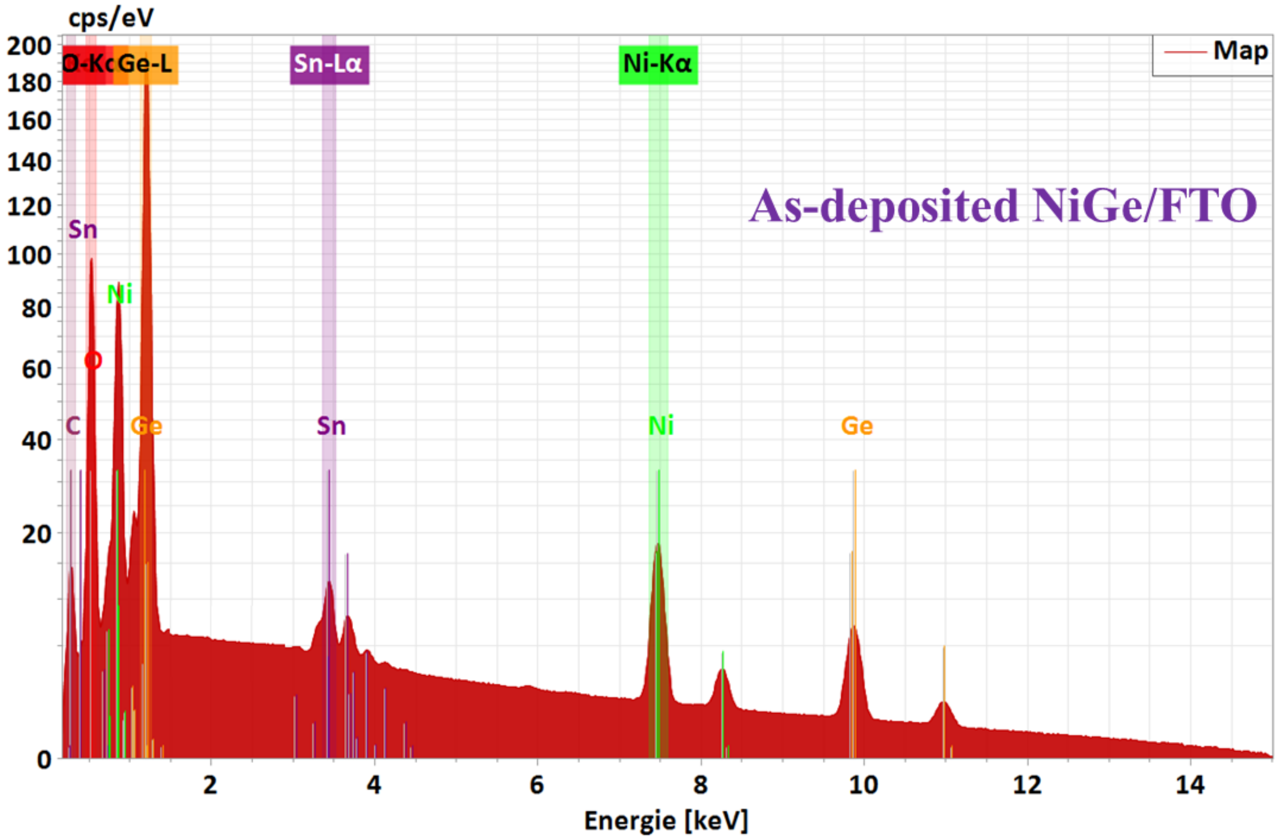

Figure S14. The EDX mapping spectrum of as-deposited NiGe/FTO film. Sn signals arise from the FTO glass substrate electrode.

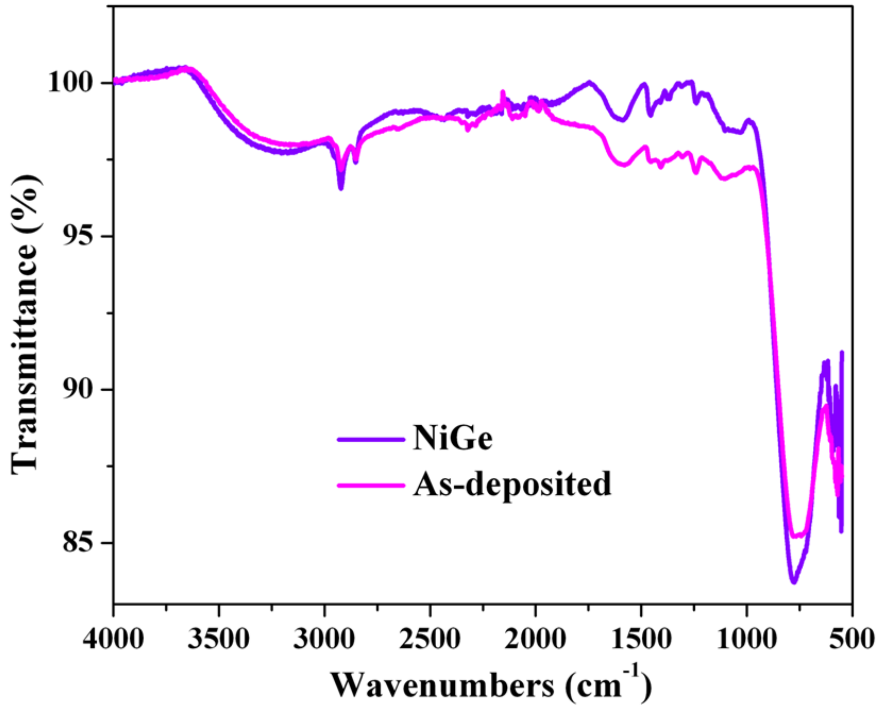

Figure S15. The FT-IR spectra of as-prepared powder and as-deposited NiGe/FTO film. No much difference in the spectra was attained confirming the retention in the chemical stability of the NiGe film after electrodeposition.

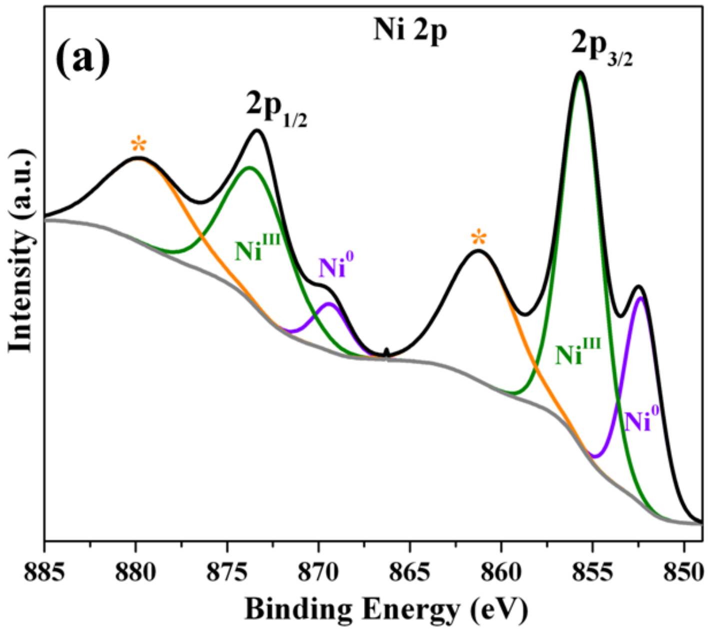

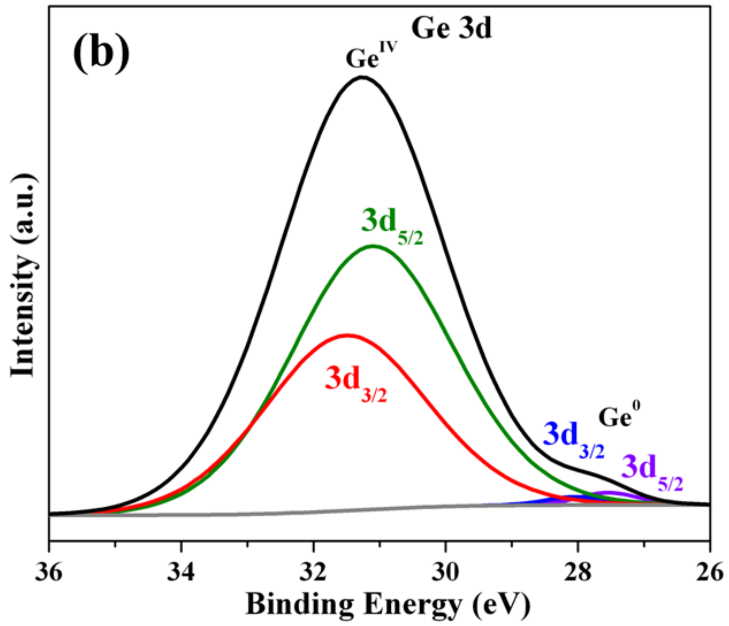

Figure S16. The high-resolution deconvoluted (a) Ni 2p and (b) Ge 3d XPS spectra of as-deposited NiGe/FTO. Similar XPS spectra of Ni 2p and Ge 3d further confirmed retention of the structure after electrophoretic deposition (Figure S11). The details of the deconvolution peaks are given in Figure S11.

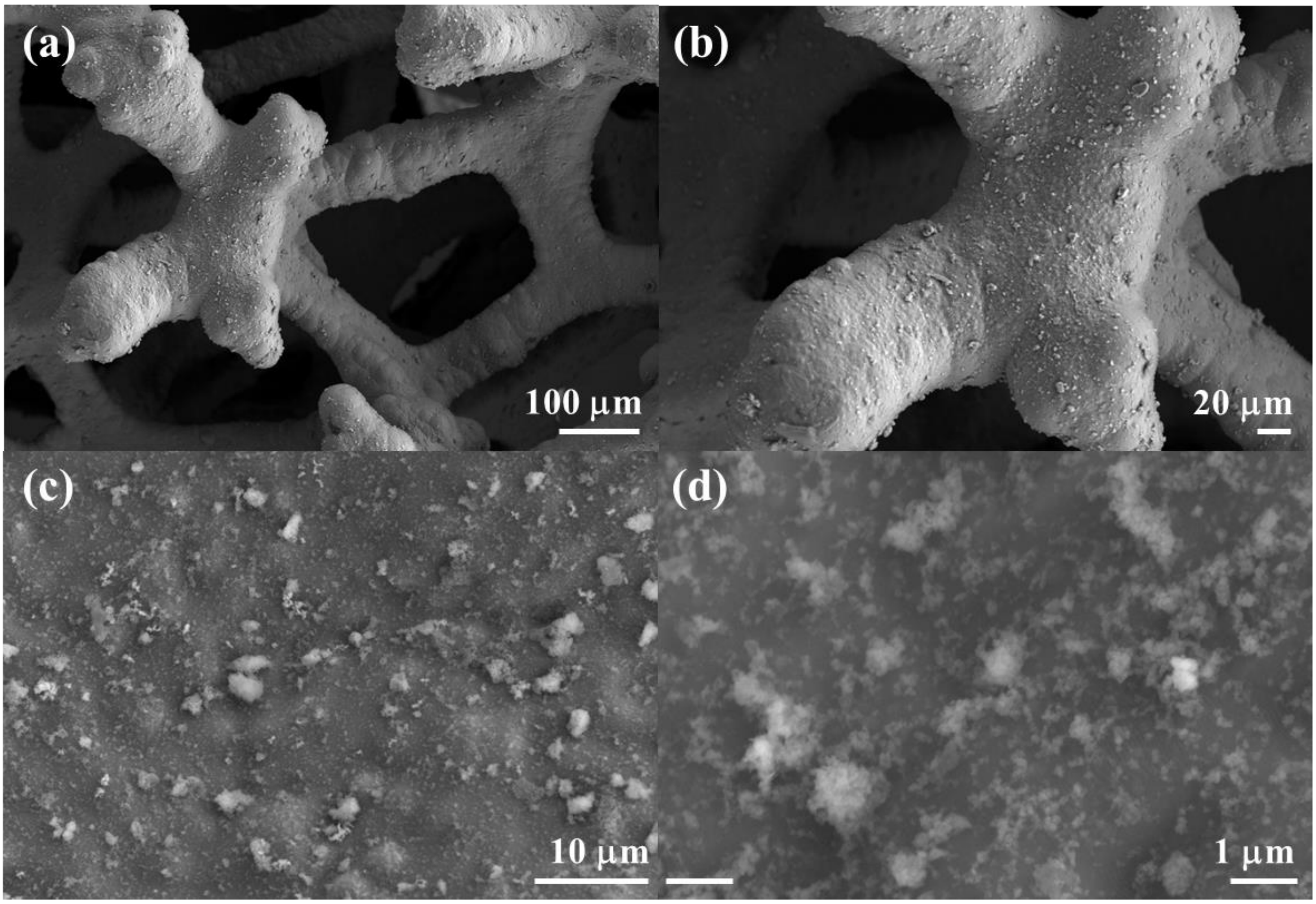

Figure S17. The SEM images (a-d) of NiGe films on NF (NiGe/NF) at different magnifications.

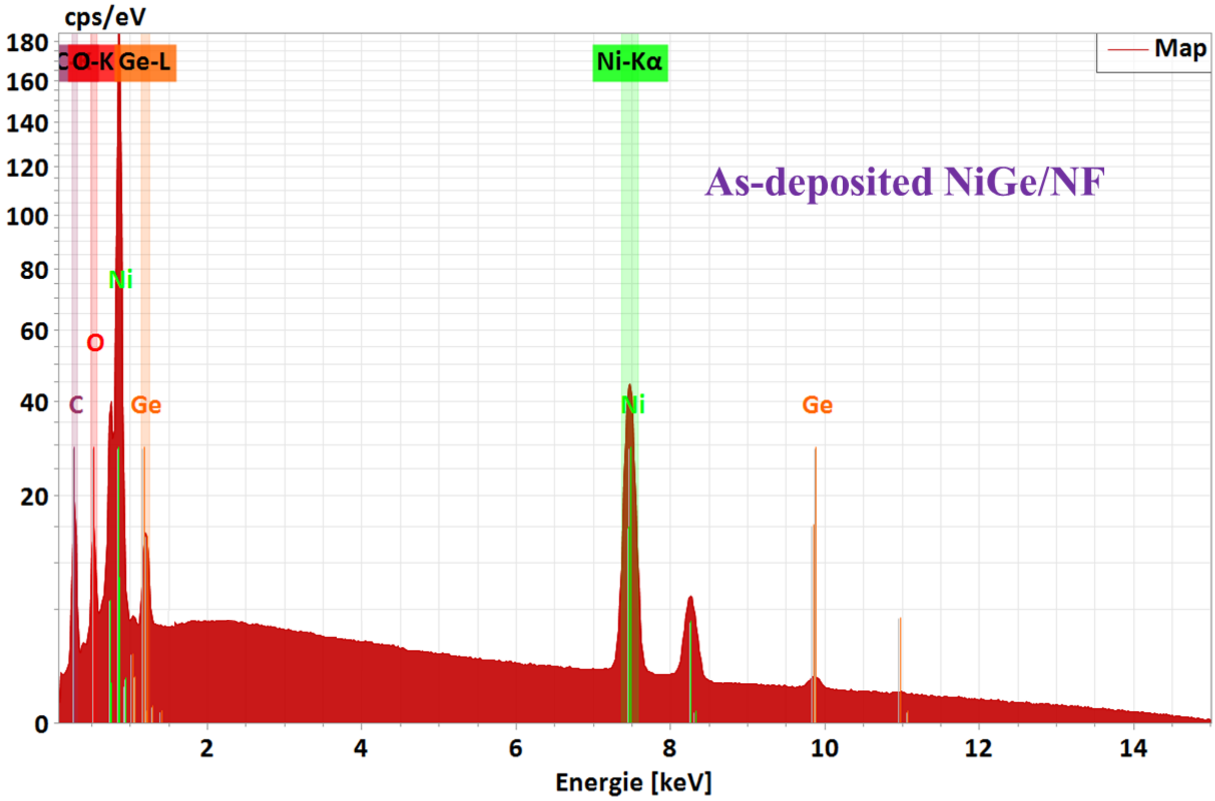

Figure S18. The EDX mapping spectrum of NiGe/NF.

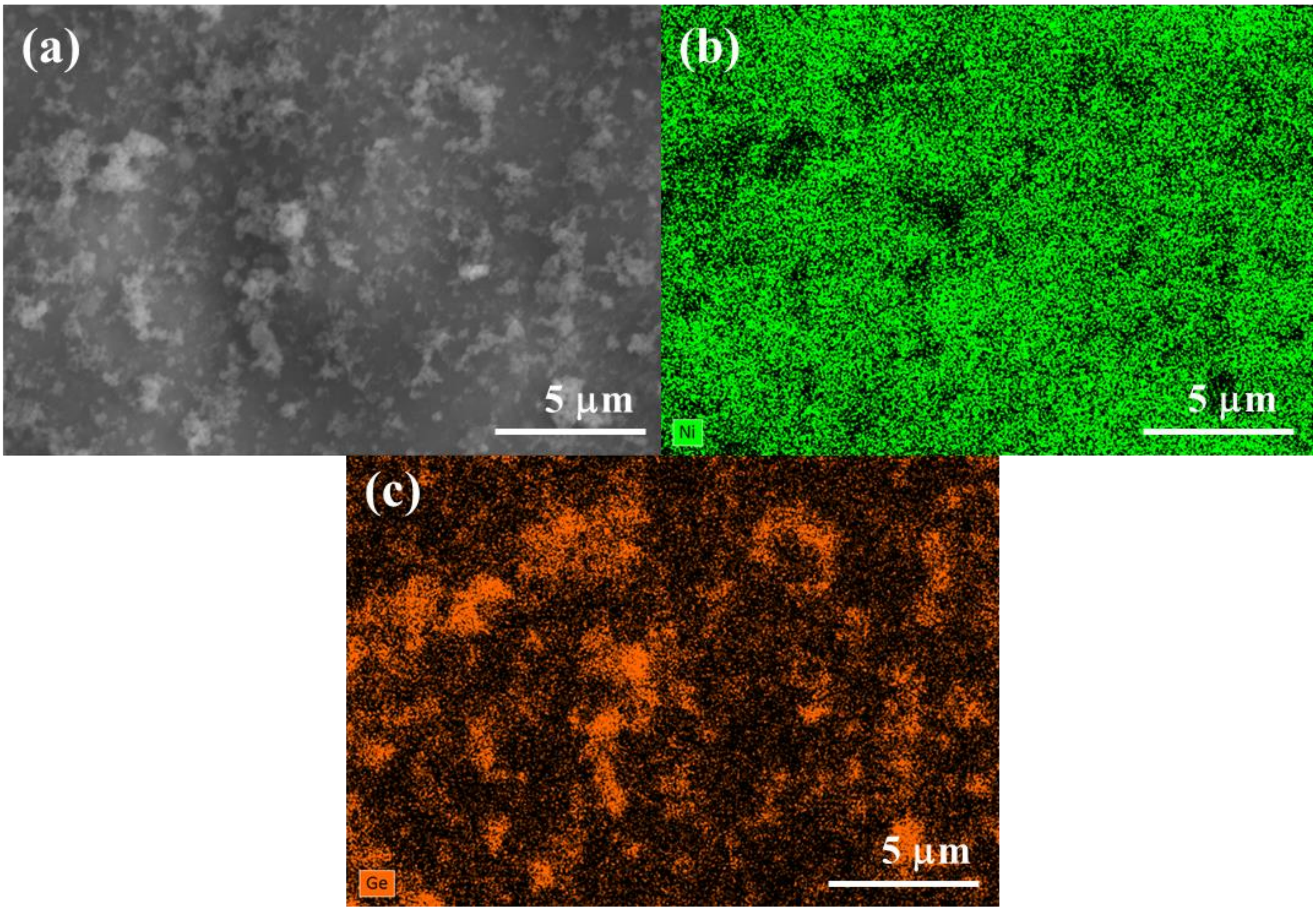

Figure S19. The SEM image (a) and the respective EDX mapping of NiGe/NF film (b-c). The spectra exhibit homogenously distributed nickel (b) and germanium (c). Notably, the results are also consistent with NiGe/FTO evidencing the chemical stability of NiGe upon deposition (oxygen (<1%) content).

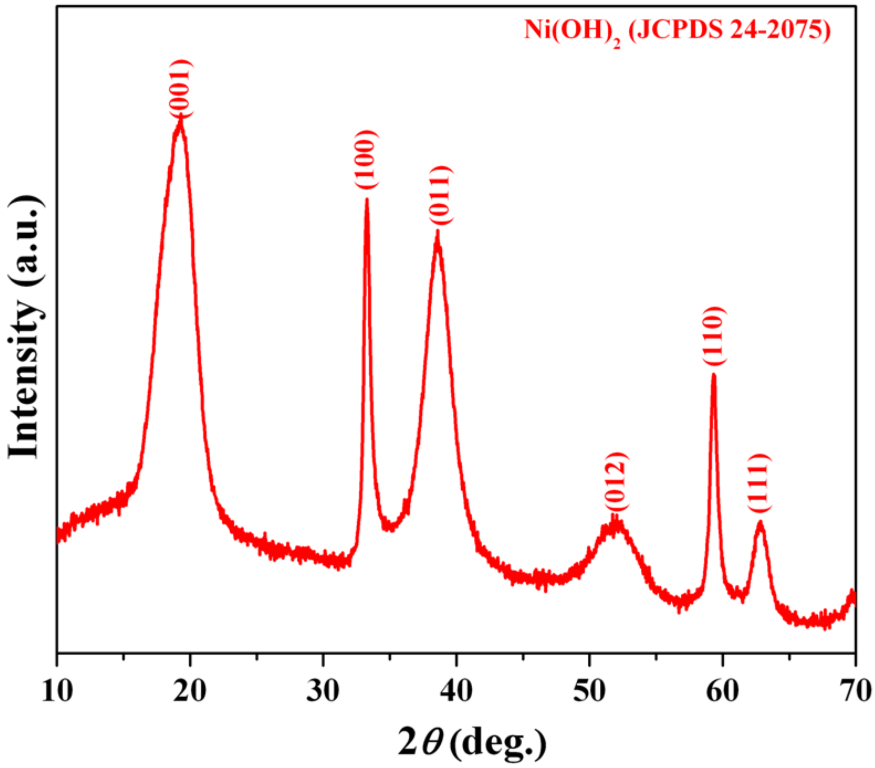

Figure S20. The PXRD pattern and Miller indices of as-synthesized $Ni(OH)_2$ (JCPDS 24-2075).

Table S6. R_{ct} (Ω) R_s (Ω), CPE($F \times s^{(a_2-1)}$) and a_2 of the investigated materials deposited on NF and its comparison to the bare NF substrate (see Figure 3b for the equivalent circuit).

Material	R_{ct} (Ω)	R_s (Ω)	CPE ($F \times s^{(a_2-1)}$)	a_2
NF	48 ±1.2	1.3 ±0.3	$1.5 \times 10^{-1} \pm 1.0 \times 10^{-2}$	$9.4 \times 10^{-1} \pm 7 \times 10^{-2}$
NiGe/NF	3.8 ± 0.3	1.5 ± 0.5	$1.1 \times 10^{-1} \pm 7 \times 10^{-2}$	$8.9 \times 10^{-1} \pm 1.0 \times 10^{-2}$
$Ni(OH)_2$/NF	5.3 ± 0.5	1.4 ± 0.6	$8.9 \times 10^{-1} \pm 1.2 \times 10^{-2}$	$9 \times 10^{-1} \pm 1.0 \times 10^{-1}$
NiOOH/NF	8 ± 2	1.6 ± 0.6	$3.2 \times 10^{-1} \pm 1.1 \times 10^{-2}$	$8.2 \times 10^{-1} \pm 9 \times 10^{-2}$

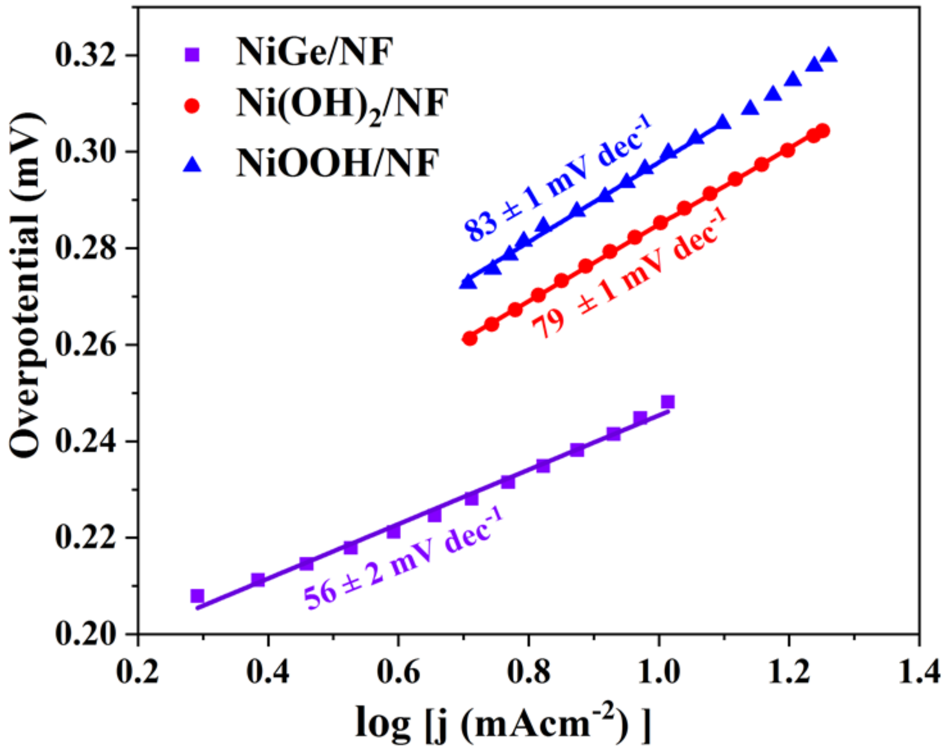

Figure S21. Tafel plots of NiGe/NF, $Ni(OH)_2$/NF, and NiOOH/NF were obtained by OER polarization curves. The Tafel slope of NiGe/NF (56±2 $mVdec^{-1}$) was found to be substantially lower than that of $Ni(OH)_2$/NF (79±1 $mVdec^{-1}$) and NiOOH/NF (83±1 $mVdec^{-1}$) confirming the intrinsically favorable catalytic property of NiGe/NF.[10,21]

The Tafel slope was calculated to infer which of the four-electron/proton-transfer steps are rate-limiting in water oxidation.[22] The reaction is initiated by water adsorption and the formation of adsorbed OH* intermediate, which is converted to another OH species that are chemically the same but energetically different than OH* species (eqn. 1 and eqn.2, where M represents for the active site). A second proton and electron transfer step give rise to the oxide intermediate (Eqn. 3). The recombination of two oxide intermediates completes one reaction turnover (Eqn. 4).

$$M + H_2O \longrightarrow M\text{–}OH^*_{ads} + H^+ + e^- \quad (1)$$

$$M\text{–}OH^*_{ads} \longrightarrow M\text{–}OH_{ads} \quad (2)$$

$$M\text{–}OH_{ads} \longrightarrow M\text{–}O_{ads} + H^+ + e^- \quad (3)$$

$$M\text{–}O_{ads} + M\text{–}O_{ads} \longrightarrow 2M + O_2 \quad (4)$$

The Tafel slope of ~60 $mVdec^{-1}$ indicates that eqn. (1) and (2) are the rate-determining steps. When step 1 is the sole rate-determining step, the Tafel slope is measured to be ~120 $mVdec^{-1}$. Eqn. 3 and 4 results in Tafel slopes of ~40 and ~15 $mVdec^{-1}$.[23] Thus, a Tafel slope of 56 $mVdec^{-1}$ for NiGe/NF implies that both the first electron/proton transfer reaction and the second electron/proton transfer is rate-determining.[24]

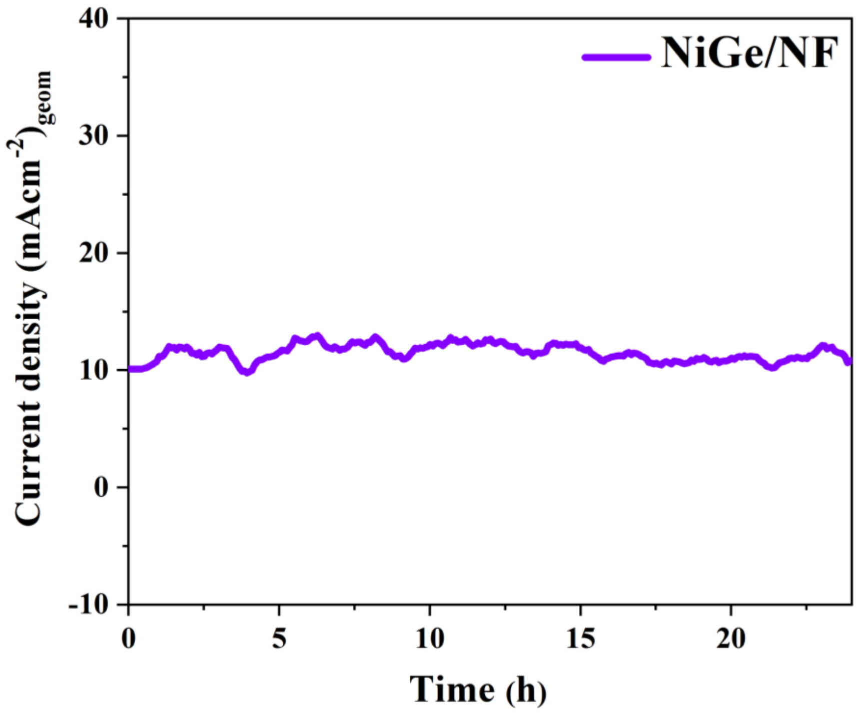

Figure S22. The CA response of NiGe/NF measured in OER at 1.48 V *vs.* RHE depicting excellent stability of the electrocatalyst for 24 h.

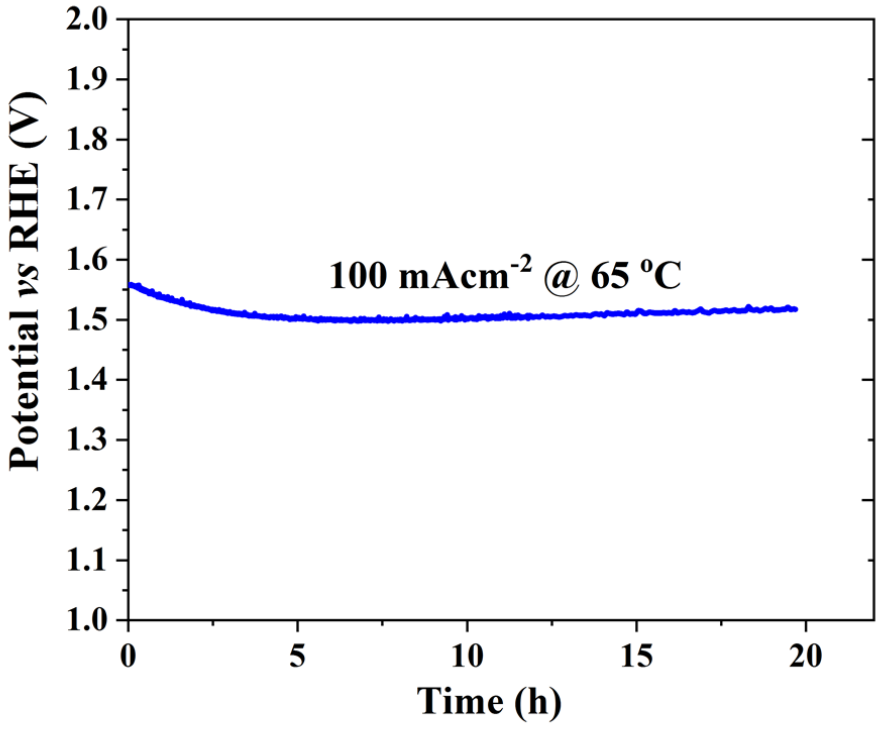

Figure S23. The chronopotentiometric response of NiGe/NF measured at a current density of 100 $mAcm^{-2}$ at 65 °C yielding a stable potential of 1.5 vs. RHE for 20 h.

Calculation of Faradaic efficiency

The Faradaic efficiency (FE) of NiGe was measured using NiGe/NF as an anode and Pt as the cathode in a closed single compartment electrochemical cell immersed in 1M aqueous KOH electrolyte. The cell and the electrolyte were first degassed with Argon for 1 h under stirring to replace any quantity of dissolved O_2 from the air (if present). First of all, we performed a blank experiment and the resulted atmosphere at the headspace was measured by GC. Afterward, the constant current density of 10 mAcm^{-2} was applied for 300 s. At the end of electrolysis, the gaseous samples were taken out of the headspace by a gas-tight syringe and analyzed by a GC calibrated for O_2. Every injection step was repeated at least three times, and the average value is presented. Finally, for the determination of FE of O_2, the amount of trace O_2 was subtracted from the volume of produced oxygen obtained from the GC.

The calculation of the FE for OER and HER was conducted based on the following equations:

$$FE(H_2,\%) = \frac{V_{H_2} \times 2 \times F}{V_m \times j \times t} \times 100\%$$

$$FE(O_2,\%) = \frac{V_{O_2} \times 4 \times F}{V_m \times j \times t} \times 100\%$$

V_{H2} and V_{O2} are the evolved volume of hydrogen and oxygen, F is the Faraday constant (96485.33289 C mol^{-1}), V_m is the molar volume of the gas, j is the current density and t is the time period of electrolysis (in s).

The experiments were conducted by applying a current density of 10 mA cm^{-2} (measured value of 9.13 mA cm^{-2}) and subsequently, the evolved H_2 and O_2 at the headspace were measured. The volume of H_2 and O_2 were found to be 0.333 mL and 0.164 mL by GC/MS. Therefore, by replacing these values in the above equations:

$$FE(H_2,\%) = \frac{0.332\ \mathrm{mL} \times 2 \times 96485.33289\ \mathrm{C\ mol^{-1}}}{24453.09\ \mathrm{mL} \times 0.00913\ \mathrm{A} \times 300\ \mathrm{s}} \times 100\% = 96\%$$

$$FE(O_2,\%) = \frac{0.164\ \mathrm{mL} \times 4 \times 96485.33289\ \mathrm{C\ mol^{-1}}}{24453.09\ \mathrm{mL} \times 0.00913\ \mathrm{A} \times 300\ \mathrm{s}} \times 100\% = 95\%$$

Calibration was performed relative to Pt, as it is known to produce 100% of FE for H_2. Therefore, we have also calculated the FE of O_2 relative to H_2. The value of FE is:

$$FE(O_2,\%) = \frac{V_{O_2}}{\frac{V_{H_2}}{2}} \times 100\%$$

$$FE(O_2,\%) = \frac{0.164\ \mathrm{mL}}{\frac{0.332\ \mathrm{mL}}{2}} \times 100\% = 99\%$$

Table S7 Calculation of Faradaic efficiency for NiGe/NF at a current density (j) of 10 mAcm^{-2}

	t (s)	V_{H_2} (mL)	V_{O_2} (mL)	V_{H_2}:V_{O_2}	FE (H_2, %)	FE (O_2, %)	FE O_2 (relative to H_2)*
NiGe/NF‖Pt	300	0.332 ± 0.006	0.164 ± 0.003	2.02 ± 0.03	96 ± 1%	95 ± 2%	99 ± 1 %

*the FE of O_2 relative to H_2 was calculated assuming 100% of FE for H_2 with Pt.

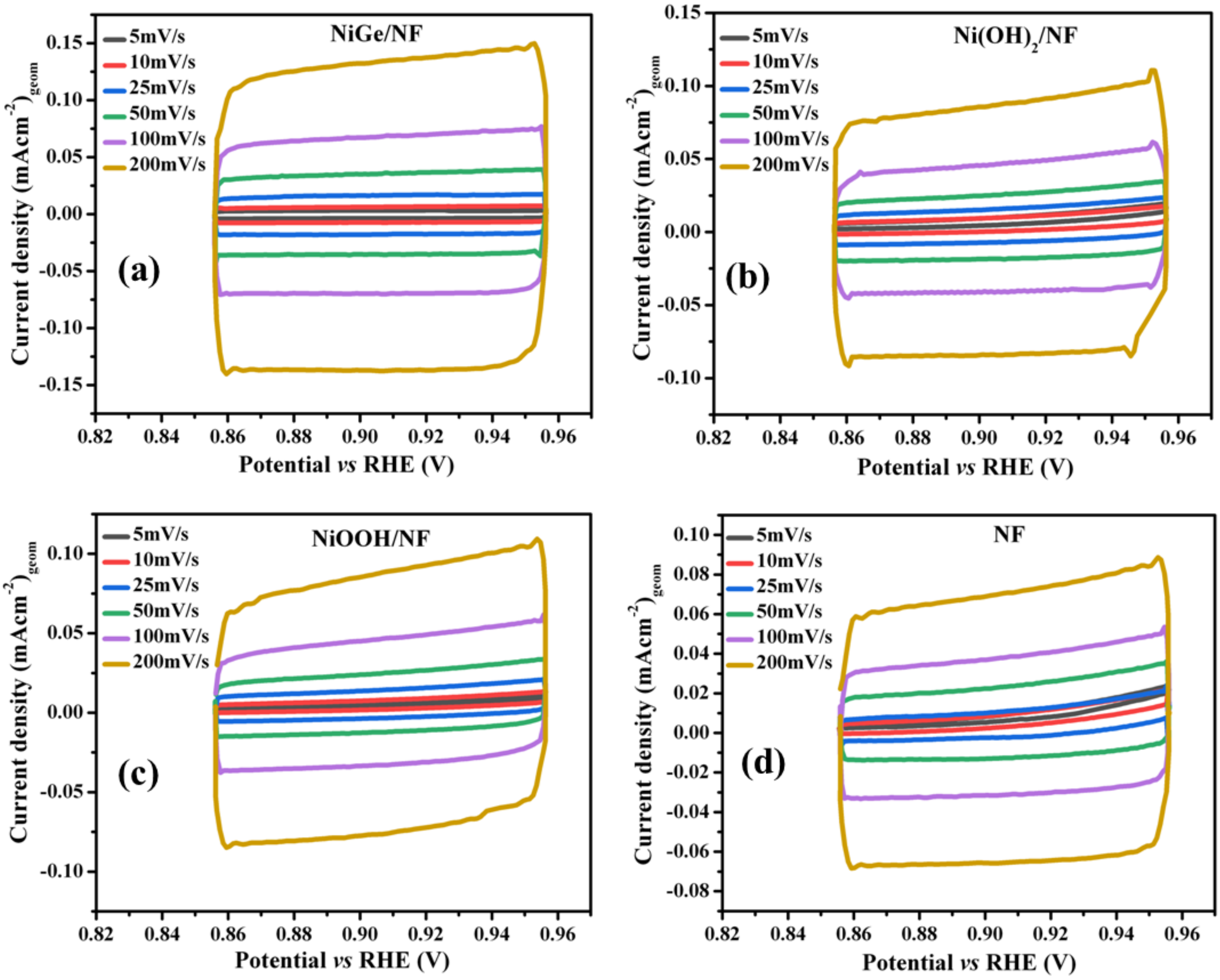

Figure S24. The ECSA analysis of (a) NiGe/NF, (b) $Ni(OH)_2$/NF, (c) NiOOH/NF, and (d) NF. The CV scans were conducted in a non-Faradaic potential range in 1 M aqueous KOH solution at a sweep rate of 5 mVs^{-1}, 10 mVs^{-1}, 25 mVs^{-1}, 50 mVs^{-1}, 100 mVs^{-1}, and 200 mVs^{-1} Half of the differences in current density variation ($\Delta J = (J_{cathodic}-J_{anodic})/2$) at a potential of 0.91 V vs. RHE plotted against scan rate fitted to a linear regression allows the determination of double-layer capacitance (C_{dl}).

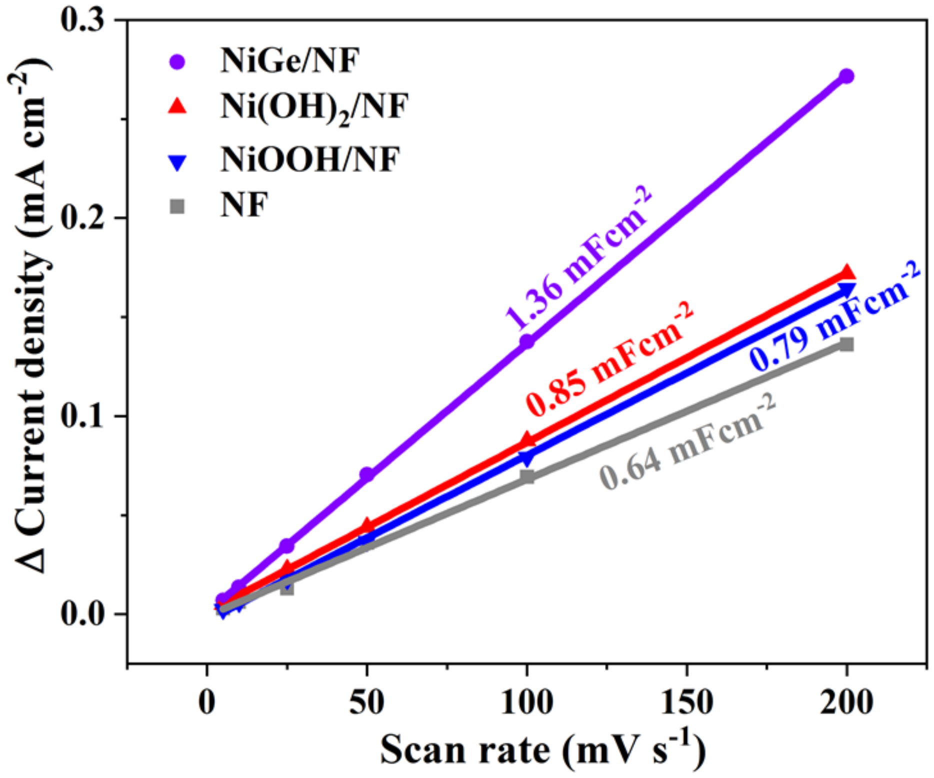

Figure S25. Current density difference at 0.91 V vs. RHE (see Figure. S23) plotted against scan rate fitted to linear regression to determine the double-layer capacitance (C_{dl}). A C_{dl} value of 1.36±0.04 mFcm^{-2} was attained for NiGe/NF whereas much lower values of 0.85±0.02, 0.79±0.01, and 0.64±0.01 mFcm^{-2} were obtained for $Ni(OH)_2$/NF, NiOOH/NF and NF, respectively.

Table S8 The BET values, C_{dl} values on NF and C_{dl} values on FTO of NiGe and the reference $Ni(OH)_2$ and NiOOH (pre)catalysts.

(pre)Catalyst	BET (m^2g^{-1})	C_{dl} on NF (mFcm^{-2})	C_{dl} on FTO (mFcm^{-2})
NiGe	17.30±0.9	1.36±0.04	0.050±0.01
$Ni(OH)_2$	14.02±1	0.85±0.02	0.023±0.005
NiOOH	270.01±2	0.79±0.01	0.021±0.01

Table S9. The comparison of OER (η) overpotentials of NiGe with other benchmark catalysts (with error bars) synthesized and tested using our three-electrode set-up in aqueous 1 M KOH in identical conditions.

Catalyst	j (mAcm^{-2})	η (mV) on FTO	η (mV) on NF
**NiGe**	**10**	**322±2**	**228±3**
$Ni(OH)_2$	10	380±6	292±5
NiOOH	10	444±6	308±5
$Co(OH)_2$	10	383±5	283±3
CoOOH	10	406±4	321±5
Co_3O_4	10	380±4	278±5
FeOOH	10	610±7	324±6
$FeNi_2O_4$	10	334±3	230±3
IrO_2	10	450±3	315±5
RuO_2	10	357±5	299±6
NF	10	-	490±9
FTO	10	-	-

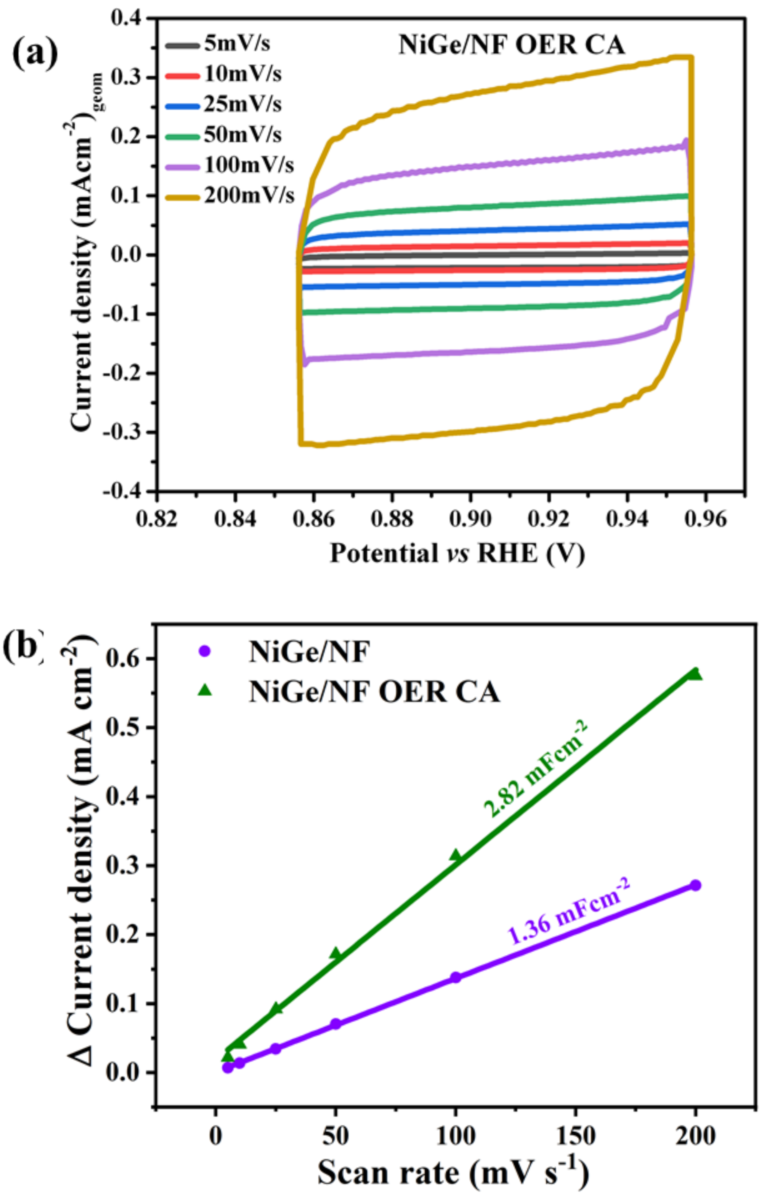

Figure S26. The ECSA analysis of NiGe/NF and NiGe/NF electrodes after 24 h of OER CA. The CV scans of NiGe/NF OER CA (for as-deposited see Figure S24-S25) were conducted in a non-Faradaic potential range in 1 M aqueous KOH solution at a sweep rate of 5 mVs^{-1}, 10 mVs^{-1}, 25 mVs^{-1}, 50 mVs^{-1}, 100 mVs^{-1}, and 200 mVs^{-1} Half of the differences in current density variation ($\Delta J = (J_{cathodic}-J_{anodic})/2$) at a potential of 0.91 V vs. RHE plotted against scan rate fitted to a linear regression allows the determination of double-layer capacitance (C_{dl}). Twice larger C_{dl} value, 2.82±0.04 $mFcm^{-2}$, was observed for NiGe/NF OER CA than that NiGe/NF (1.36±0.04 $mFcm^{-2}$) and could be attributed to the rapid loss of Ge into the electrolyte with significant structural transformation.

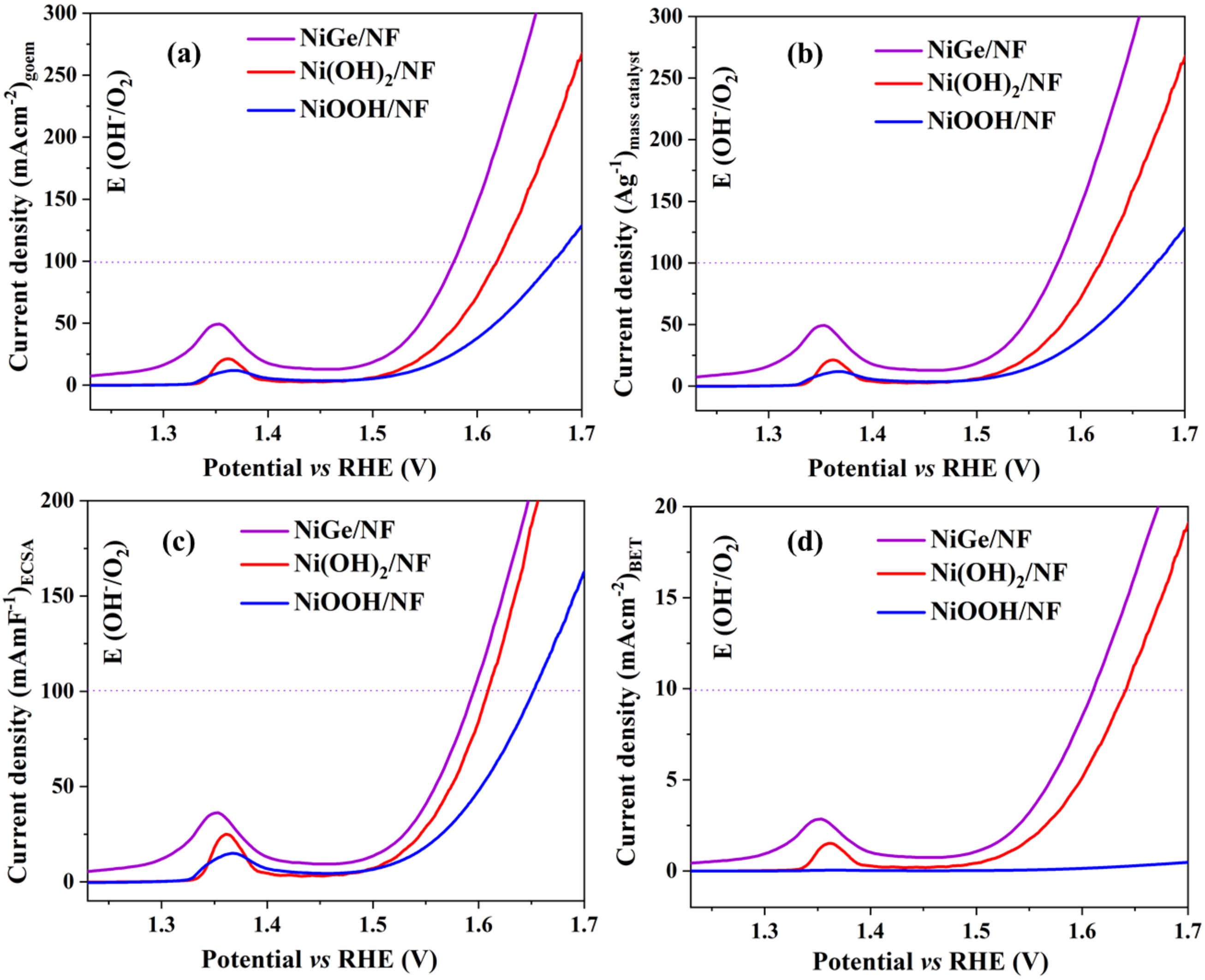

Figure S27. The LSVs normalized by (a) geometric area, (b) mass, (c) ECSA, and (d) BET of NiGe/NF, $Ni(OH)_2$/NF, and NiOOH/NF, respectively. The ECSA (C_{dl}) and BET normalized currents showed a higher amount of available active surface for NiGe/NF

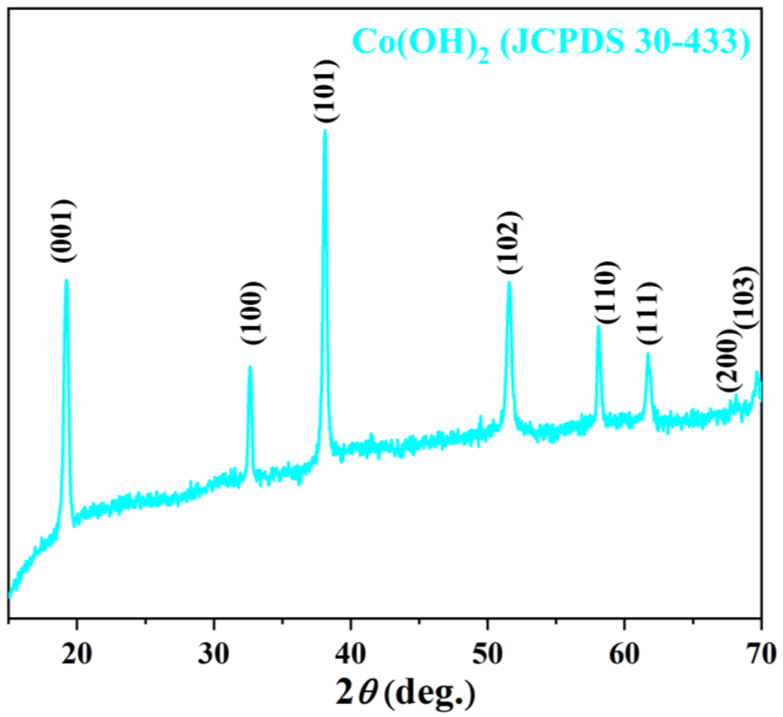

Figure S28. The PXRD pattern and the Miller indices of as-prepared $Co(OH)_2$ (JCPDS 30-433).

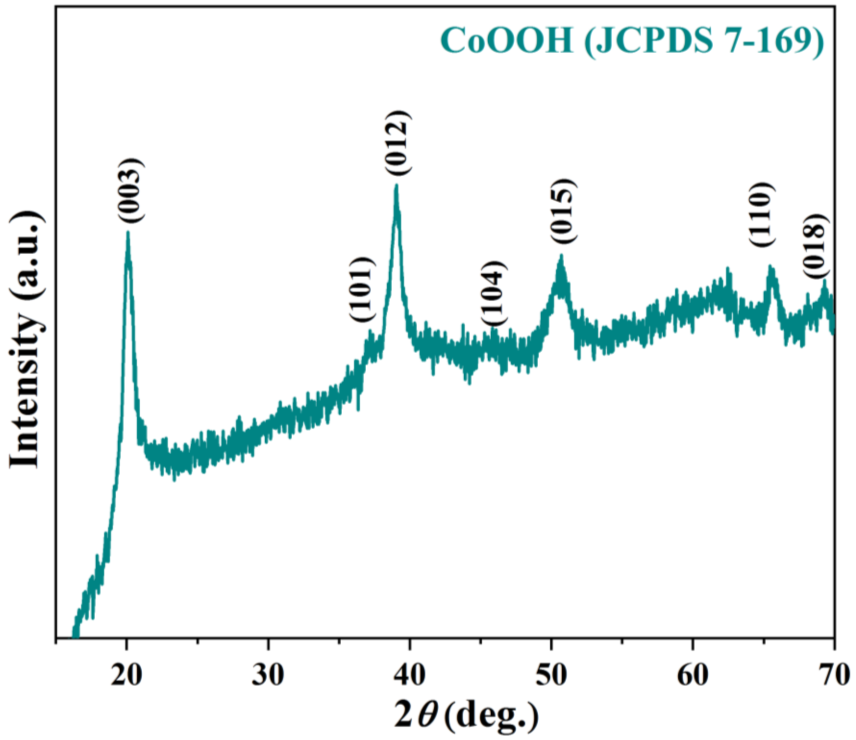

Figure S29. The PXRD pattern and the Miller indices of as-prepared CoOOH (JCPDS 7-169).

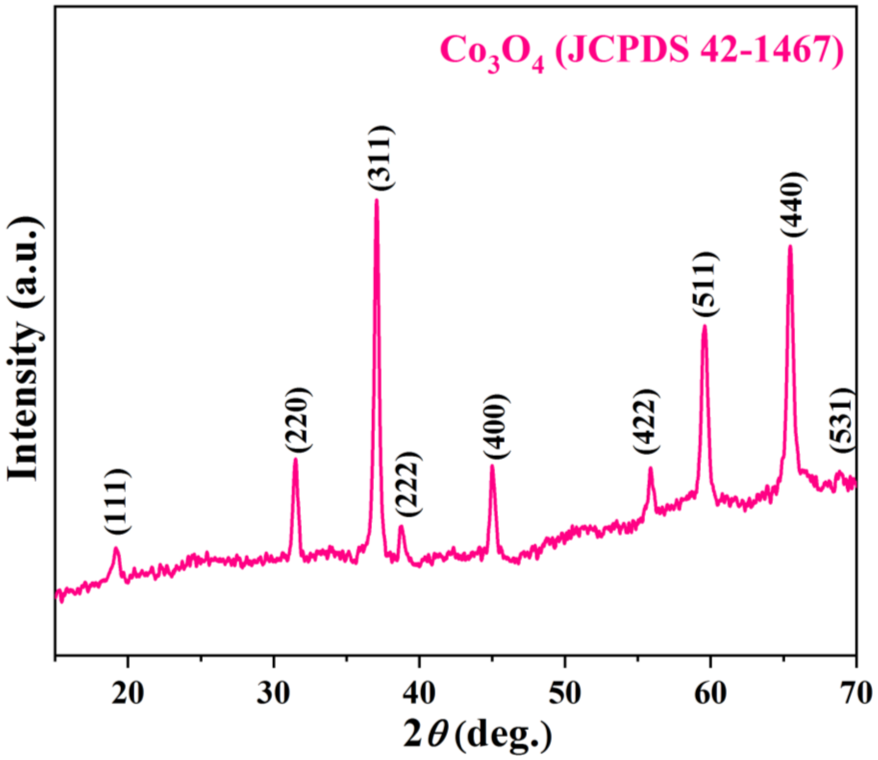

Figure S30. The PXRD pattern and the Miller indices of as-prepared Co_3O_4 (JCPDS 42-1467).

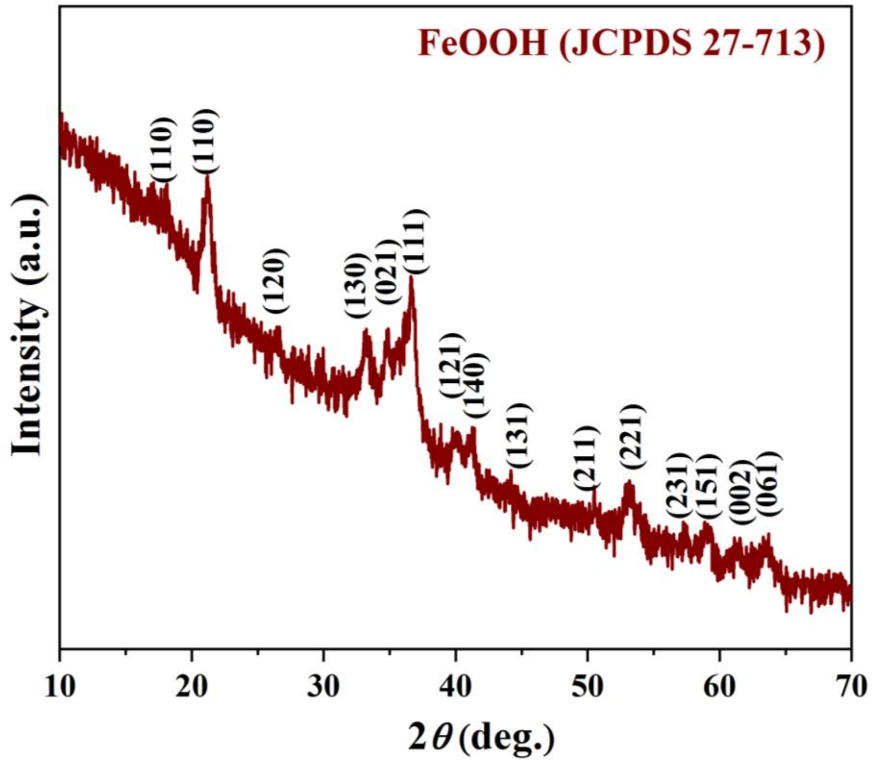

Figure S31. The PXRD pattern and the Miller indices of as-prepared FeOOH (JCPDS 27-713).

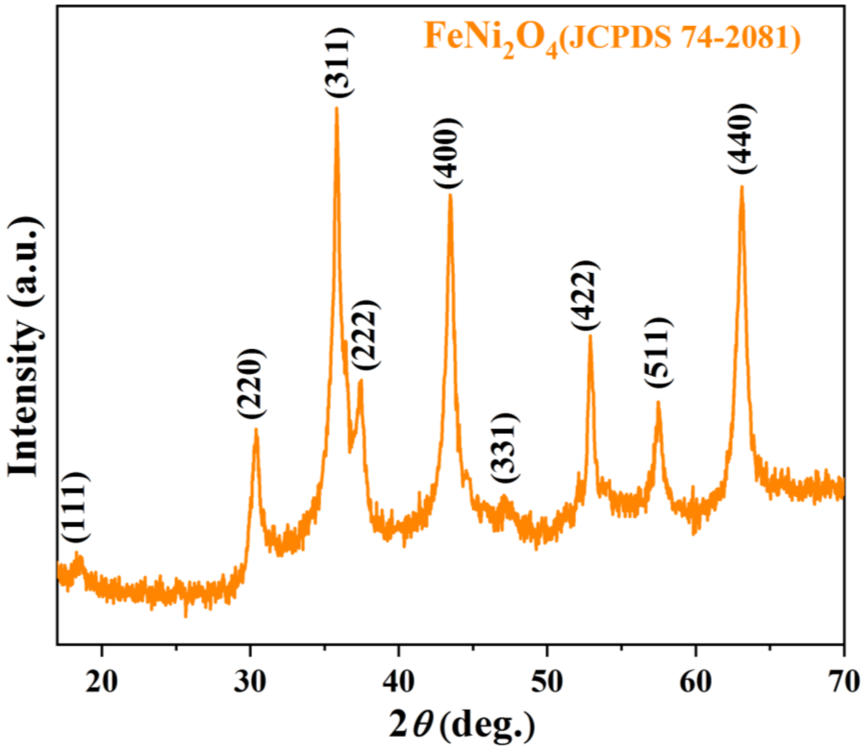

Figure S32. The PXRD pattern and the Miller indices of as-prepared FeNi2O4 (JCPDS 74-2081).

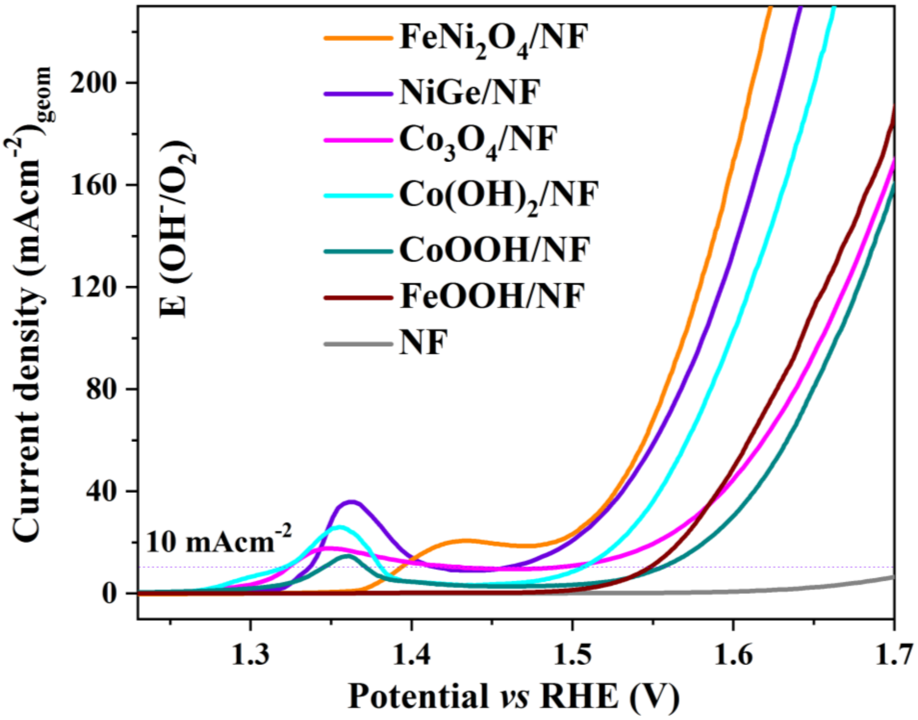

Figure S33. The comparison of LSV curves of NiGe/NF with benchmark non-noble-metal based catalysts on NF at a scan rate of 1 mVs^{-1}, which shows the superior performance of NiGe/NF with respect to the investigated Co- and Fe- catalysts and comparable activity to that best active FeNi-catalyst (see Table S6 and S7).

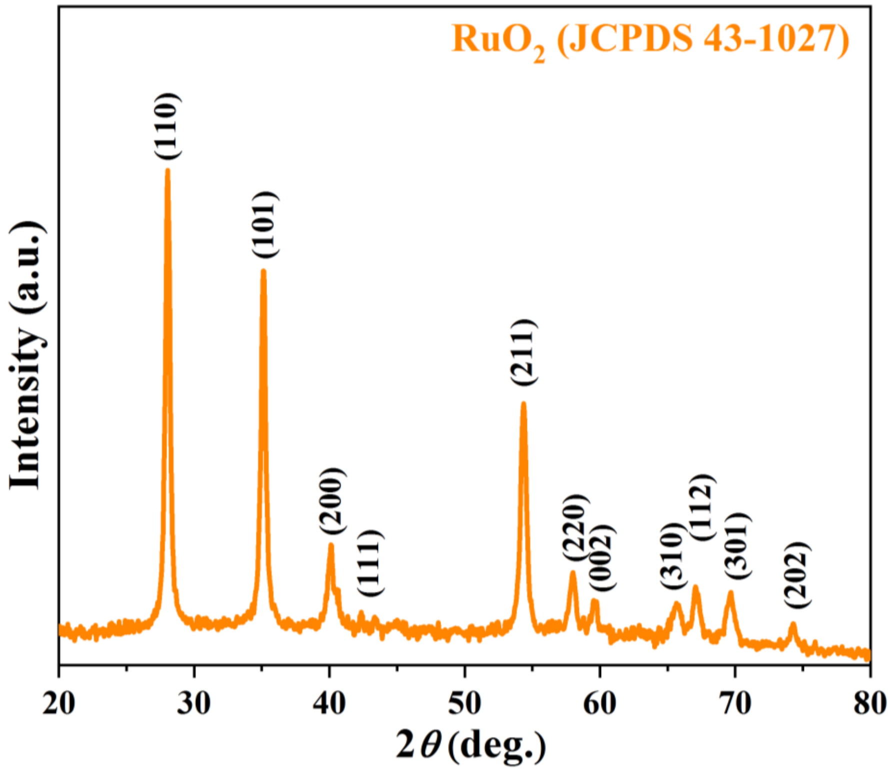

Figure S34. The PXRD pattern and the Miller indices of as-prepared RuO_2 (JCPDS 43-1027).

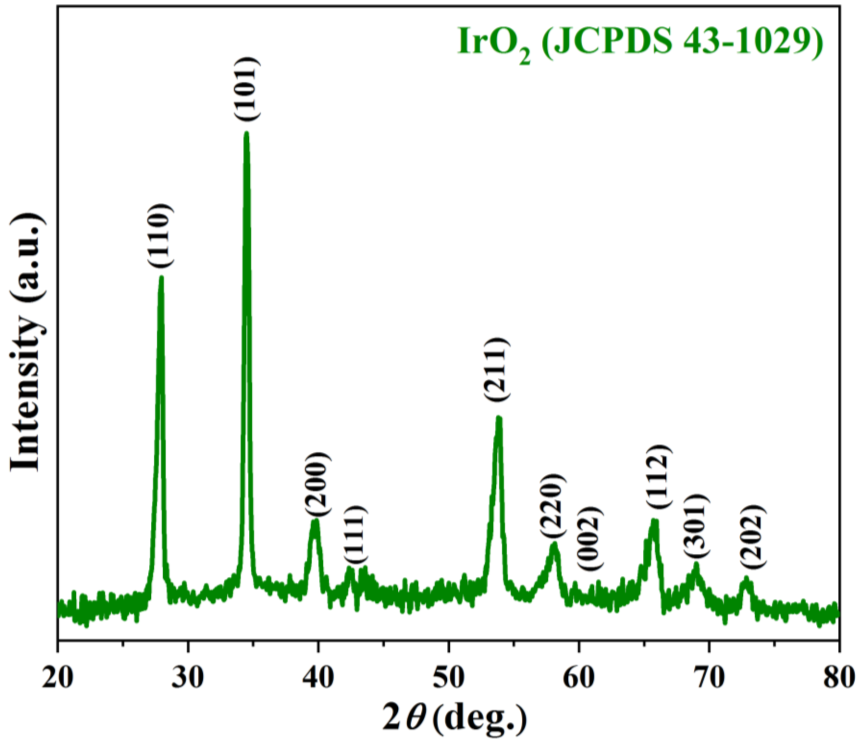

Figure S35. The PXRD pattern and the Miller indices of as-prepared IrO_2 (JCPDS 43-1029).

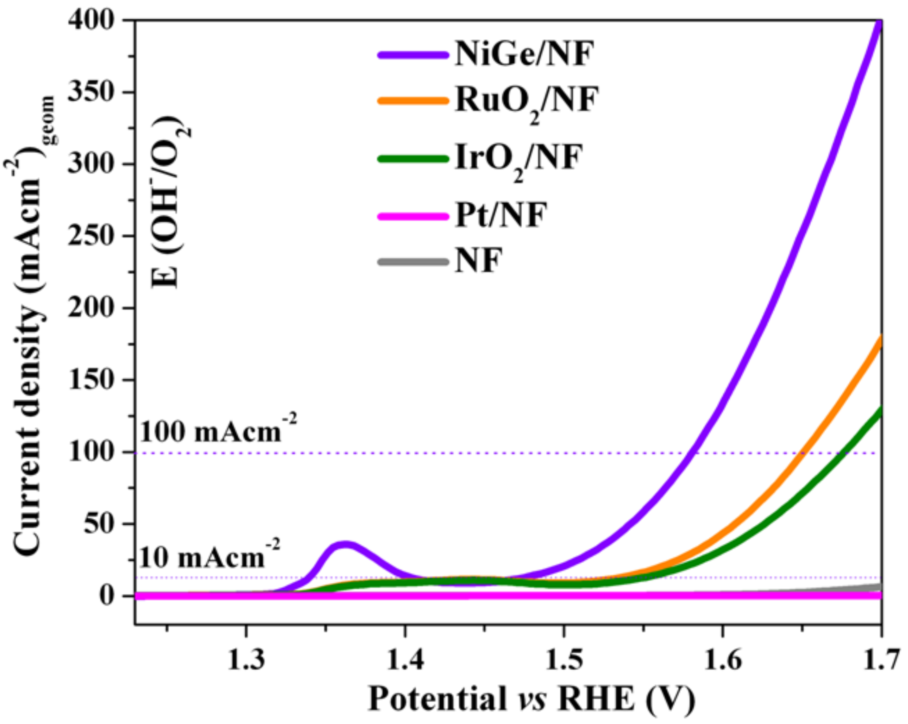

Figure S36. The comparison of LSV curves of NiGe/NF with benchmark noble-metal-based RuO_2/NF and IrO_2/NF catalysts as well as with Pt/C/NF at a scan rate of 1 mVs^{-1}, which signified the better performance of NiGe/NF with respect to the investigated catalysts (see Table S6).

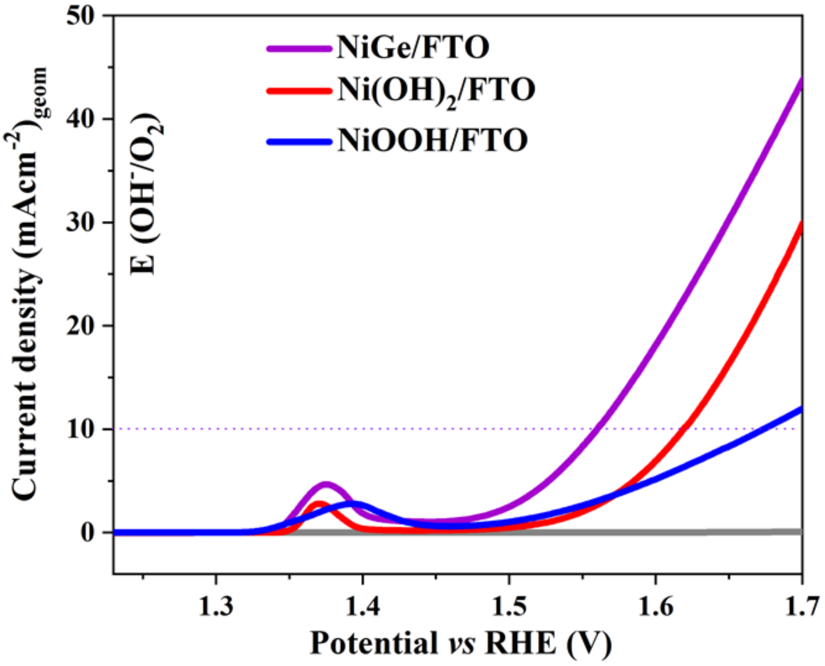

Figure S37. The LSV polarization curves of NiGe/FTO, $Ni(OH)_2$/FTO, NiOOH/FTO, and FTO at a scan rate of 5 mVs^{-1} in 1 M aqueous electrolyte. A similar trend in OER activity in comparison to NF was observed.

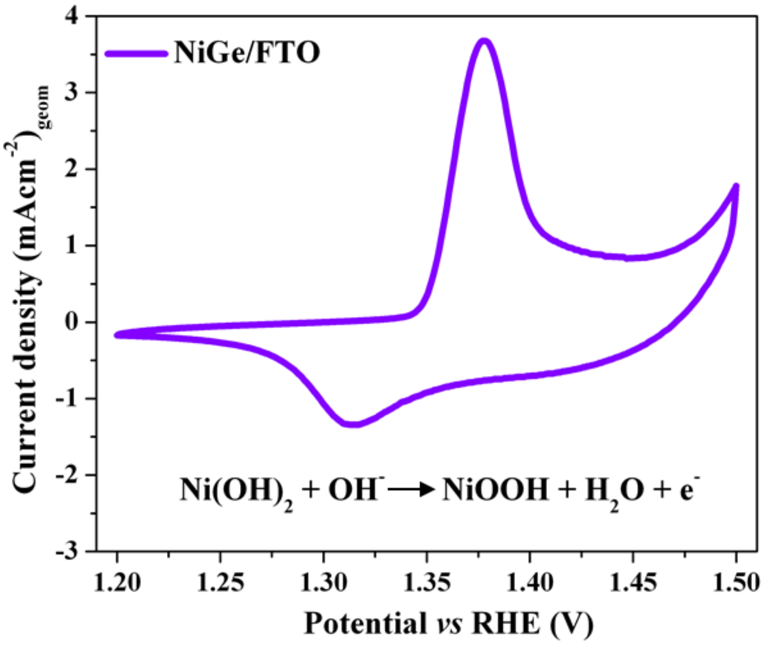

Figure S38. The CV's of NiGe/FTO measured between 1.2 to 1.5 V (vs. RHE) in 1 M aqueous KOH solution with a sweep rate of 5 mVs^{-1} featuring a redox pair corresponding to the reaction $Ni(OH)_2$ + OH- → NiOOH + H_2O + e-. From the voltammogram, it was evident that Ni^{2+} reversibly converted into Ni^{III} (NiOOH) and served as a catalytically active site for OER.

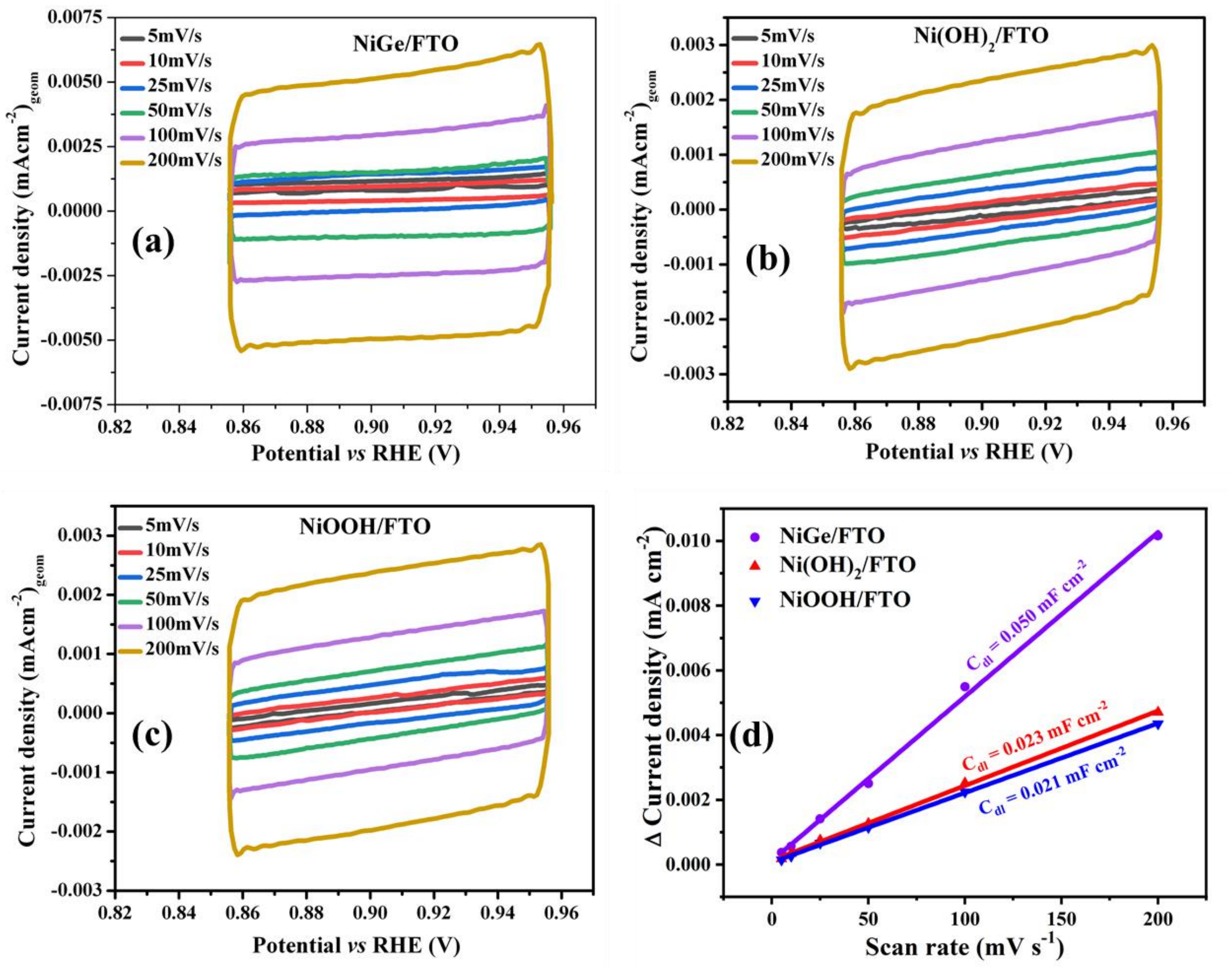

Figure S39. The ECSA analysis of (a) NiGe/FTO, (b) $Ni(OH)_2$/FTO, (c) NiOOH/FTO. The CV scans were conducted in a non-Faradaic potential range in 1 M aqueous KOH solution at a sweep rate of 5 mVs^{-1}, 10 mVs^{-1}, 25 mVs^{-1}, 50 mVs^{-1}, 100 mVs^{-1}, and 200 mVs^{-1} Half of the differences in current density variation ($\Delta J = (J_{cathodic}-J_{anodic})/2$) at a potential of 0.91 V vs. RHE plotted against scan rate fitted to linear regression (d) allows the determination of double-layer capacitance (C_{dl}). A C_{dl} value of 0.050±0.01 $mFcm^{-2}$ was attained for NiGe/FTO while substantially lower values of 0.023±0.005 and 0.021±0.01 $mFcm^{-2}$ were obtained for $Ni(OH)_2$/FTO, NiOOH/FTO, respectively.

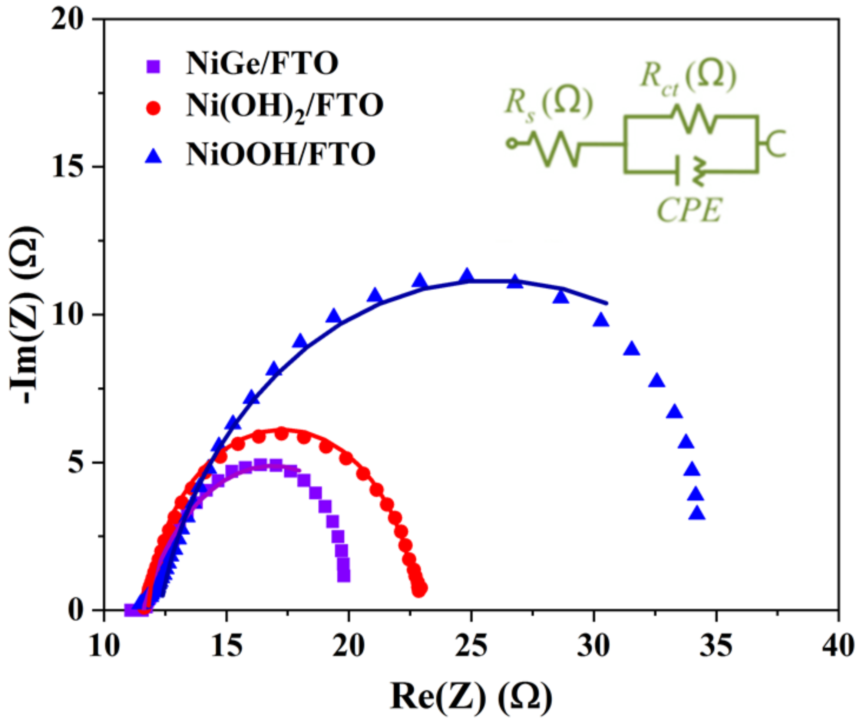

Figure S40. Nyquist plot of NiGe/FTO, $Ni(OH)_2$/FTO, and NiOOH/FTO attained from EIS fitting to an equivalent circuit (inset) at an anodic polarization potential of 1.55 V *vs.* RHE.

Table S10. R_{ct} (Ω) R_s (Ω), CPE($F \times s^{(a_2-1)}$) and a_2 of the investigated materials deposited on FTO

Material	R_{ct} (Ω)	R_s (Ω)	*CPE* ($F \times s^{(a_2-1)}$)	a_2
FTO	3653 ± 1.1	14.1 ± 0.2	$9.1 \times 10^{-6} \pm 1.2 \times 10^{-10}$	$9.6 \times 10^{-1} \pm 5 \times 10^{-2}$
NiGe/FTO	16 ± 2	16.6 ± 2	$2.6 \times 10^{-2} \pm 3 \times 10^{-3}$	$8.0 \times 10^{-1} \pm 1.0 \times 10^{-2}$
$Ni(OH)_2$/FTO	18 ± 0.5	12 ± 2	$4.6 \times 10^{-1} \pm 2 \times 10^{-2}$	$9 \times 10^{-1} \pm 1 \times 10^{-1}$
NiOOH/FTO	27 ± 1.1	12 ± 2	$4.9 \times 10^{-2} \pm 3 \times 10^{-2}$	$9 \times 10^{-1} \pm 1 \times 10^{-1}$

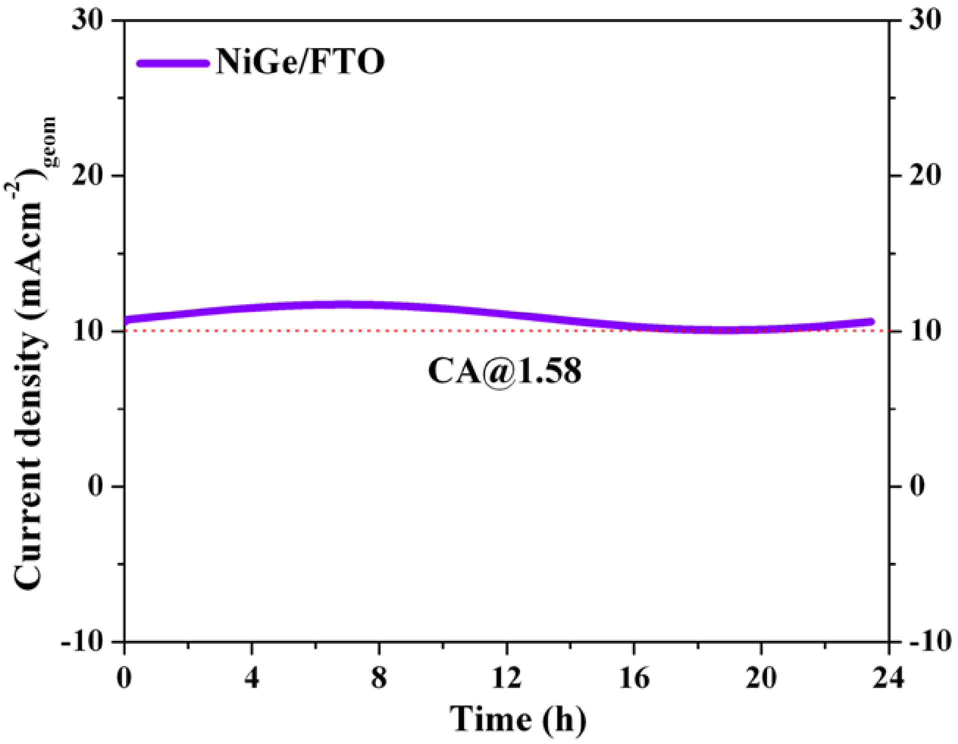

Figure S41. The CA response of NiGe/FTO measured in OER at 1.58V *vs.* RHE depicting excellent stability of the electrocatalyst. The broken red line is a guide to the eye at 10 $mAcm^{-2}$.

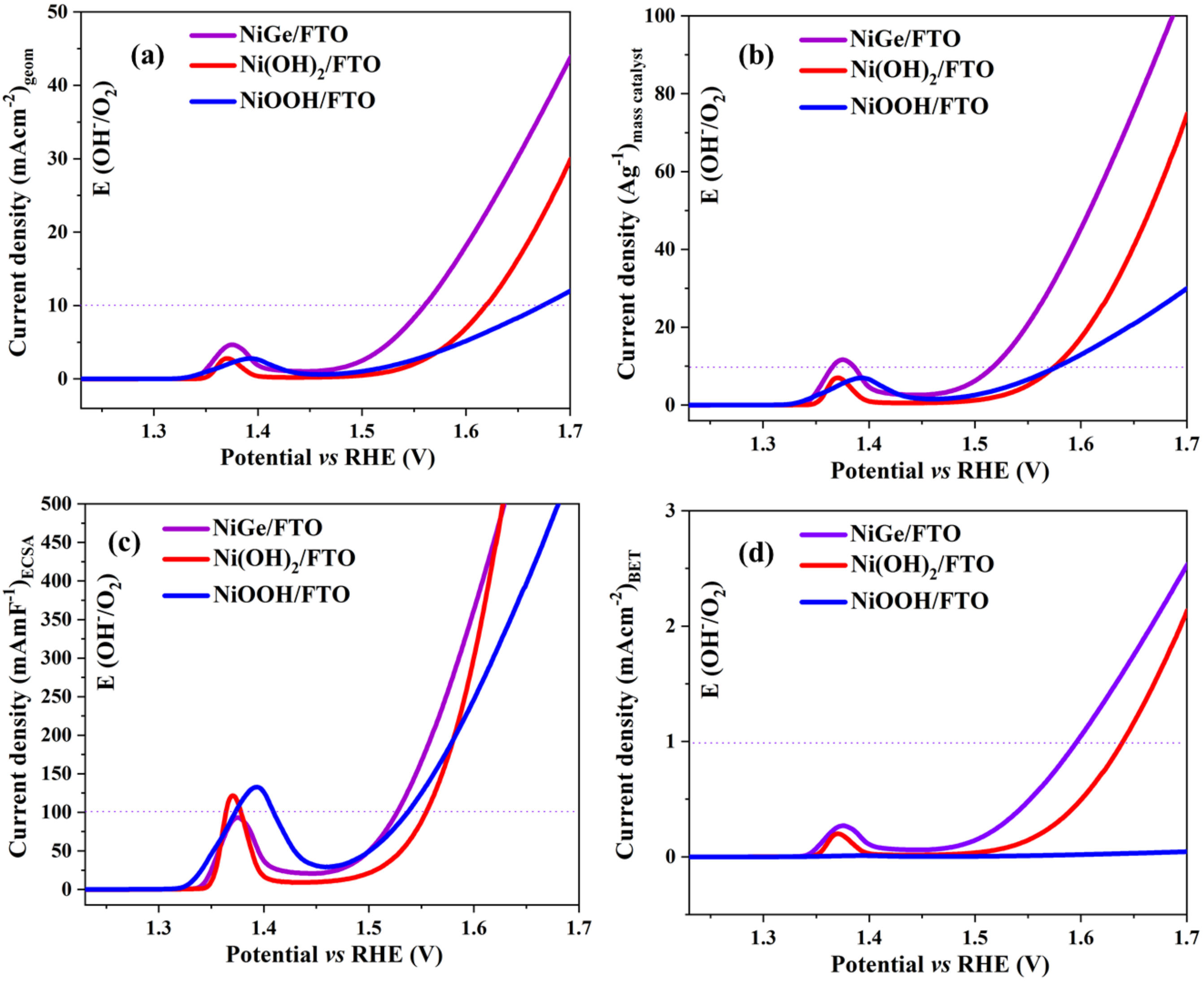

Figure S42. The LSVs normalized by (a) geometric area, (b) mass, (c) ECSA, and (d) BET of NiGe/FTO, $Ni(OH)_2$/FTO, and NiOOH/FTO, respectively.

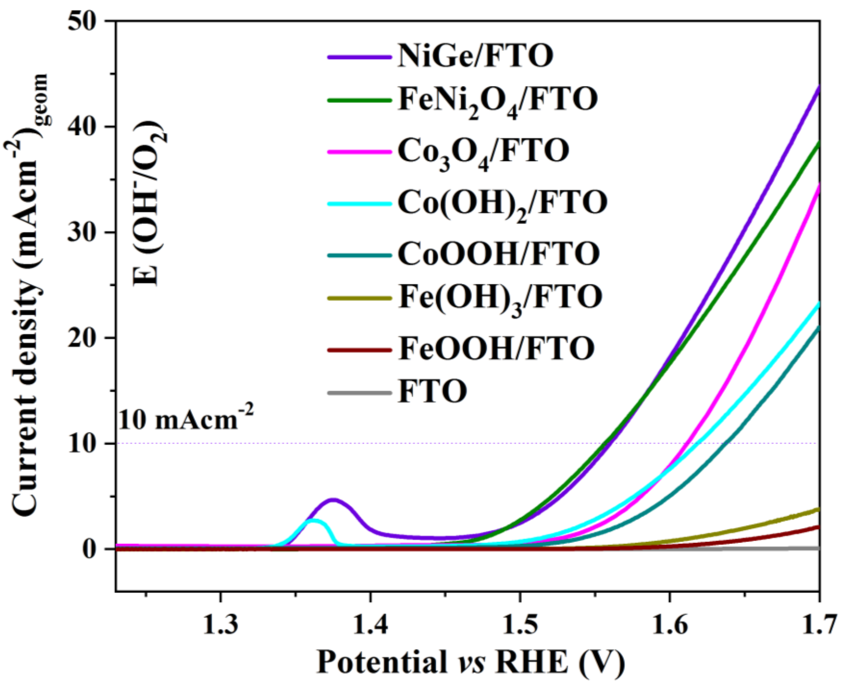

Figure S43. The comparison of LSV curves of NiGe/NF with benchmark non-noble-metal based catalysts on FTO at a scan rate of 1 mVs^{-1}, which shows the superior performance of NiGe/NF with respect to the investigated Co- and Fe- catalysts and comparable activity to that best active FeNi-catalyst (see Table S6 and S8).

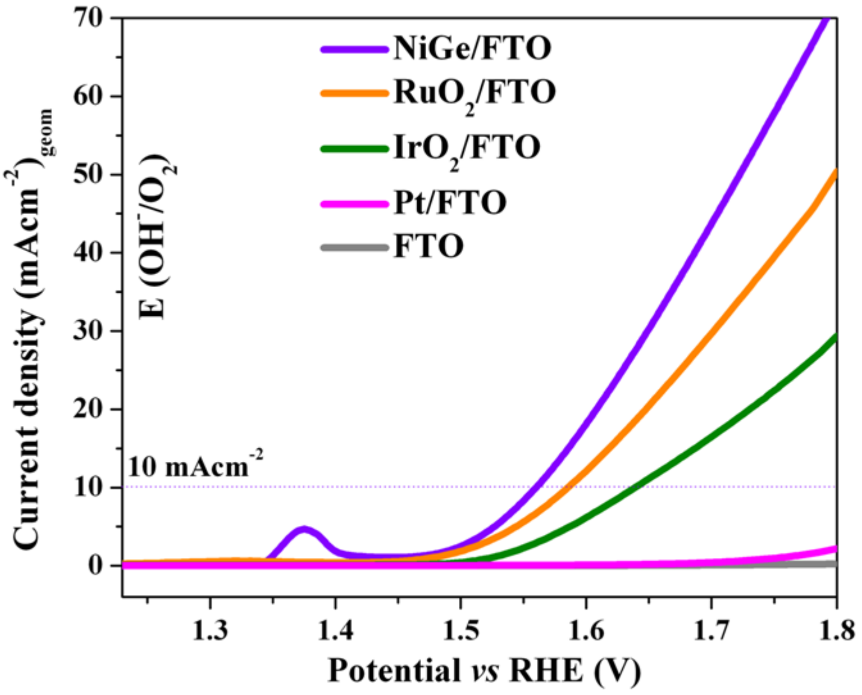

Figure S44. The comparison of LSV curves of NiGe/FTO with benchmark noble-metal-based RuO_2/FTO and IrO_2/FTO catalysts as well as with Pt/FTO at a scan rate of 1 mVs^{-1}, which signified the better performance of NiGe/FTO over the investigated catalysts (see Table S6 and S8).

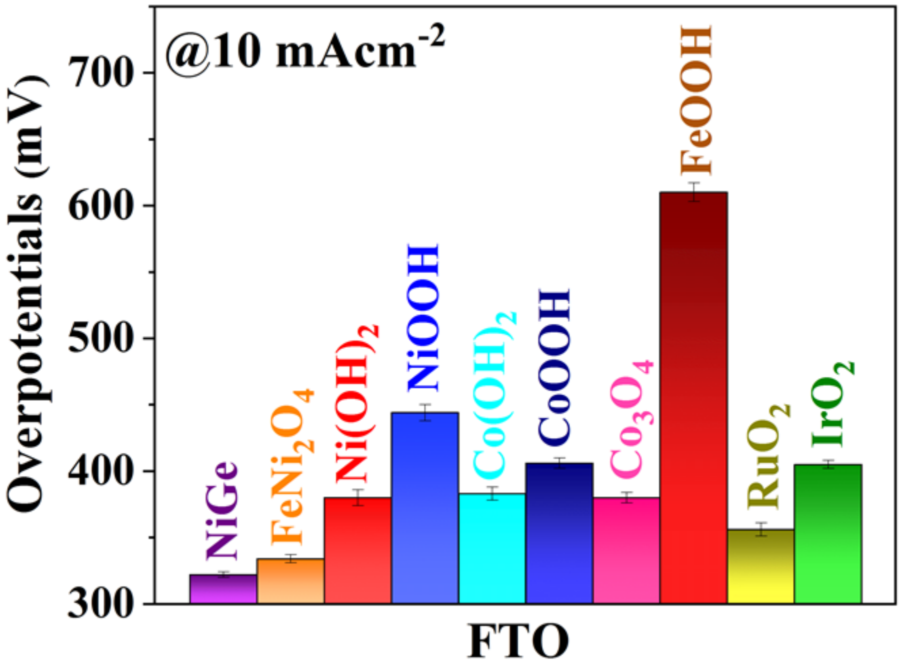

Figure S45. The bar diagram (with error bars) comparing the overpotential (mV) at 10 mAcm^{-2} of NiGe with the state-of-the-art Co-, Fe-. Ni- and NiFe-based as well as noble metal-based OER catalysts.

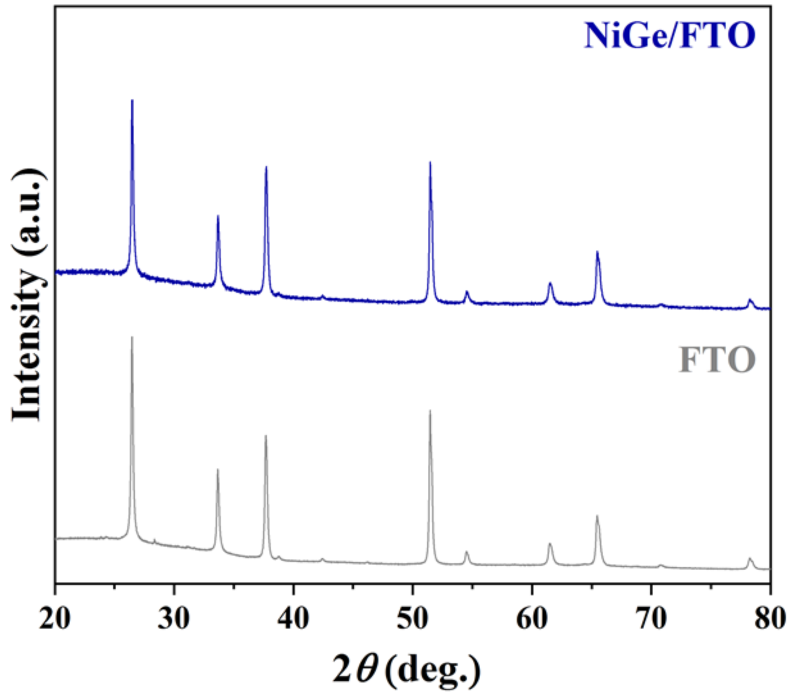

Figure S46. The PXRD pattern of NiGe/FTO films after OER CA (24 h) and bare FTO substrate. The reflections NiGe/FTO matched perfectly with FTO suggesting that the size of the particles is either small or they are amorphous in nature.

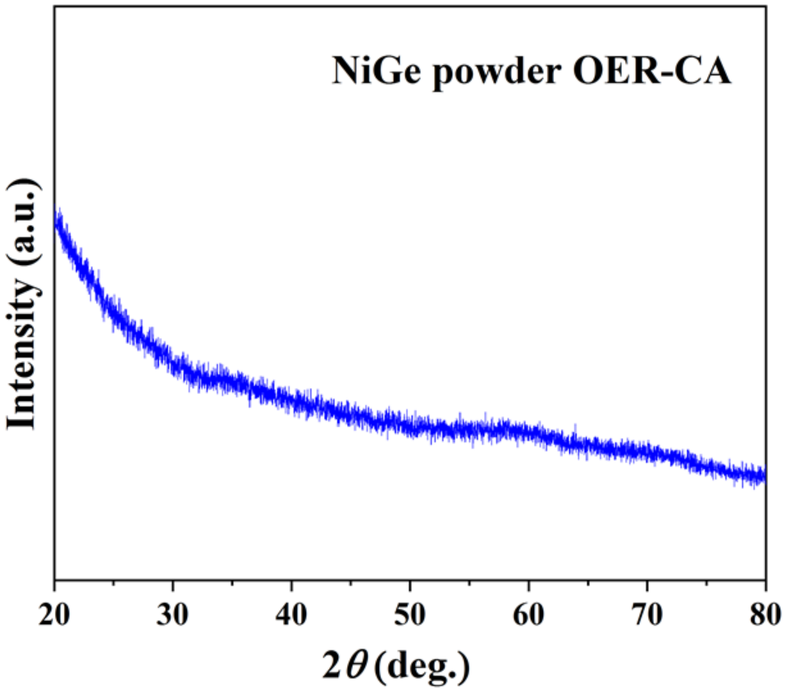

Figure S47. The particle present on the NiGe/FTO films after OER CA (24 h) were separated by sonication and subjected again for PXRD analysis. The PXRD pattern displayed a broad reflection pattern which was similar to the as-prepared NiGe. To get more insights into the active structure, we carefully conducted SEM, TEM, XPS, FT-IR, and Raman spectroscopy.

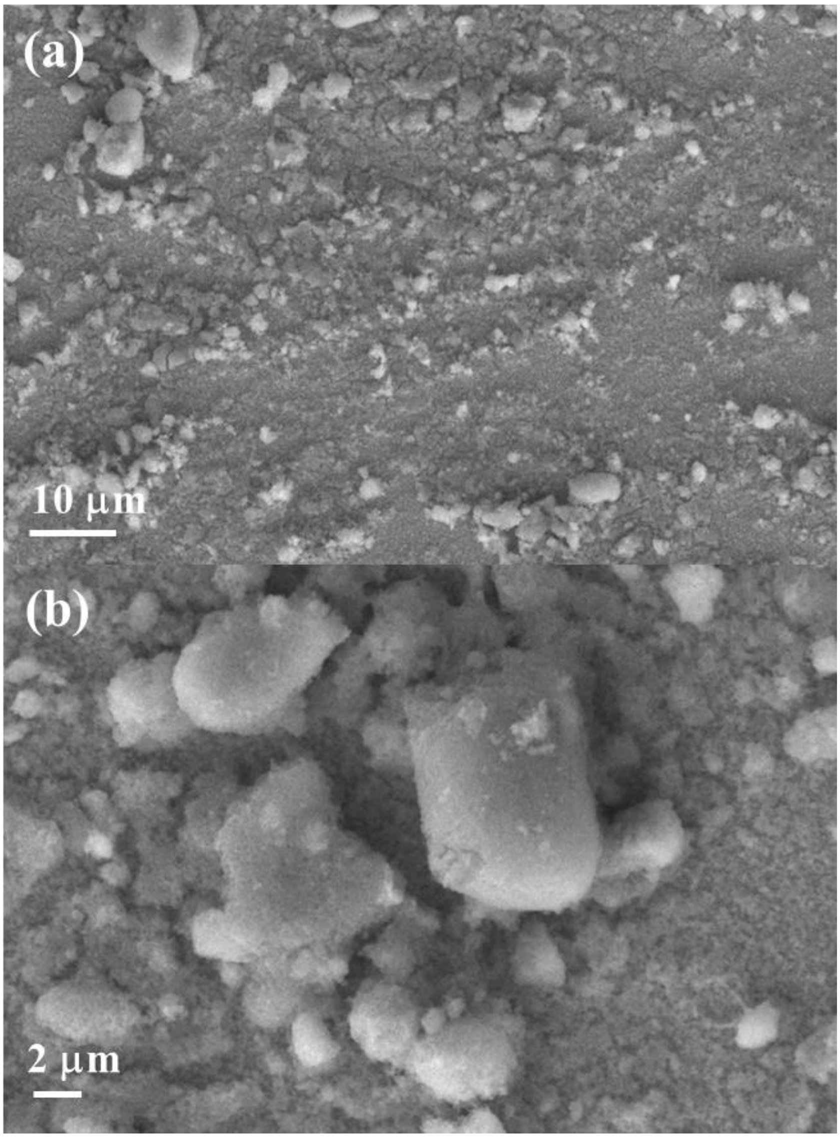

Figure S48. The SEM images (a-b) of NiGe/FTO films after OER CA (24 h) showing different magnifications. The nanoparticles displayed porous-type morphology with an agglomeration of particles. This directly indicated that the complete structural transformation of the initial NiGe phase.

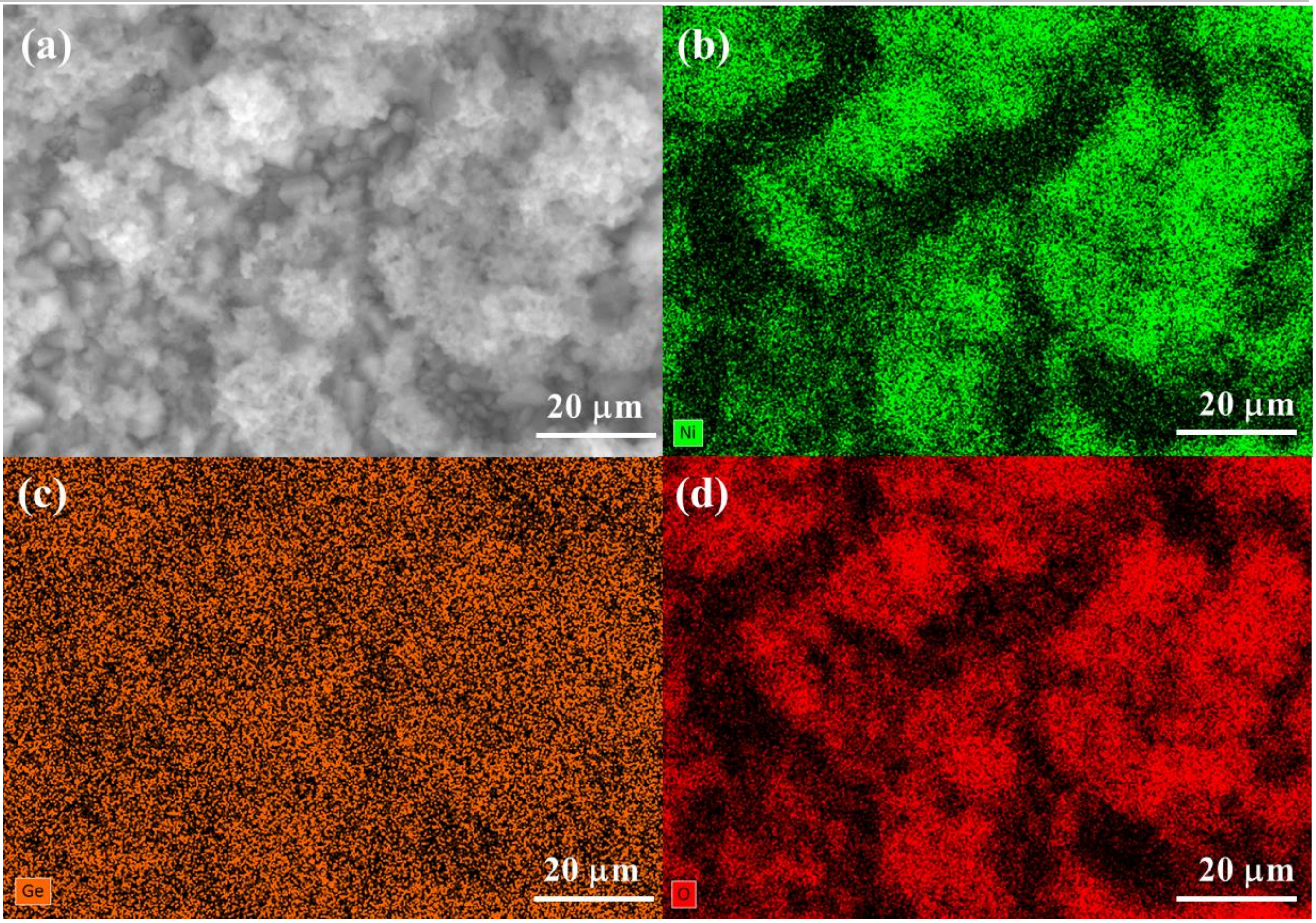

Figure S49. The SEM image (a) and the respective EDX mapping of NiGe after OER CA (b-d). The spectra exhibit a noteworthy change in the morphology as well as in the structure. Although the nickel (b) and oxygen (d) were homogeneously distributed within the particles, the germanium (c) atoms mostly disappeared from the structure specifying complete conversion of NiGe under strongly alkaline OER conditions. The composition by EDX is listed in Table S5.

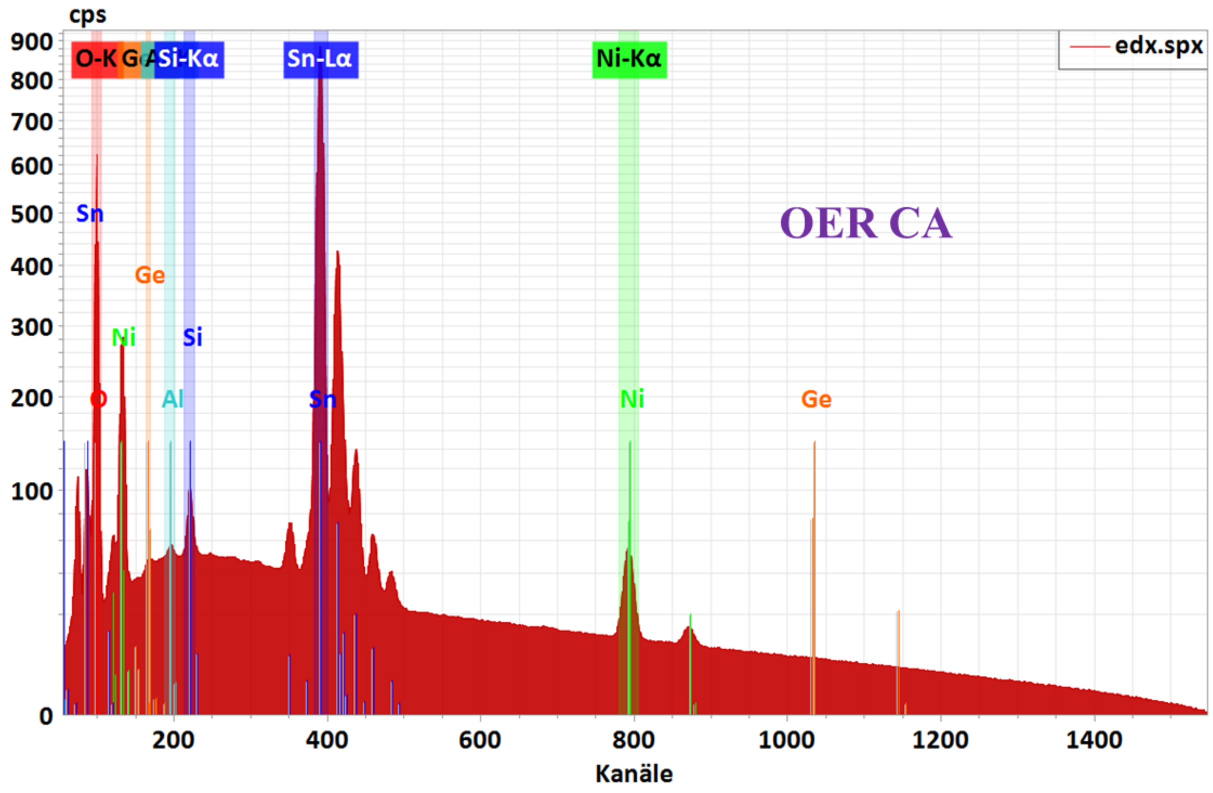

Figure S50. The EDX mapping spectrum of NiGe/FTO film after OER CA (24 h). Sn and Si signals arrises from the FTO glass substrate electrode. Al signals arise from the sample holder

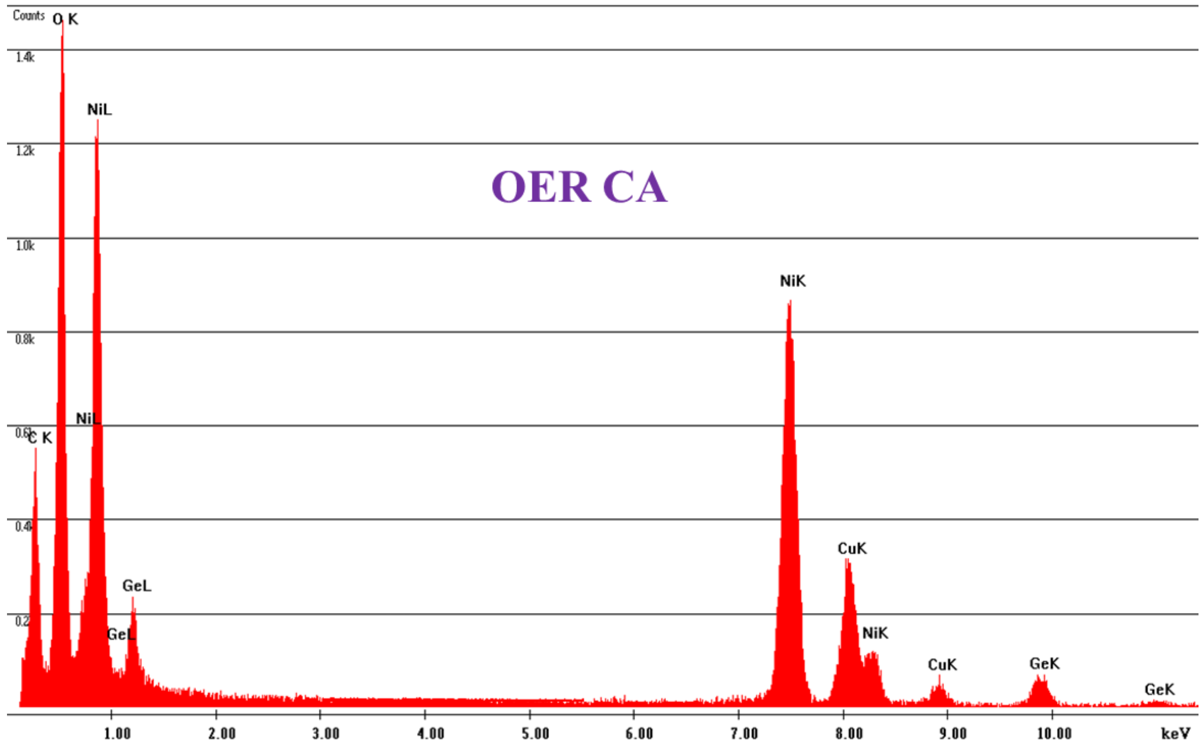

Figure S51. The EDX analysis of NiGe/FTO (on isolated particles). The peaks for copper and carbon can be unambiguously correlated to the TEM grid (carbon film on 300 mesh Cu-grid).).

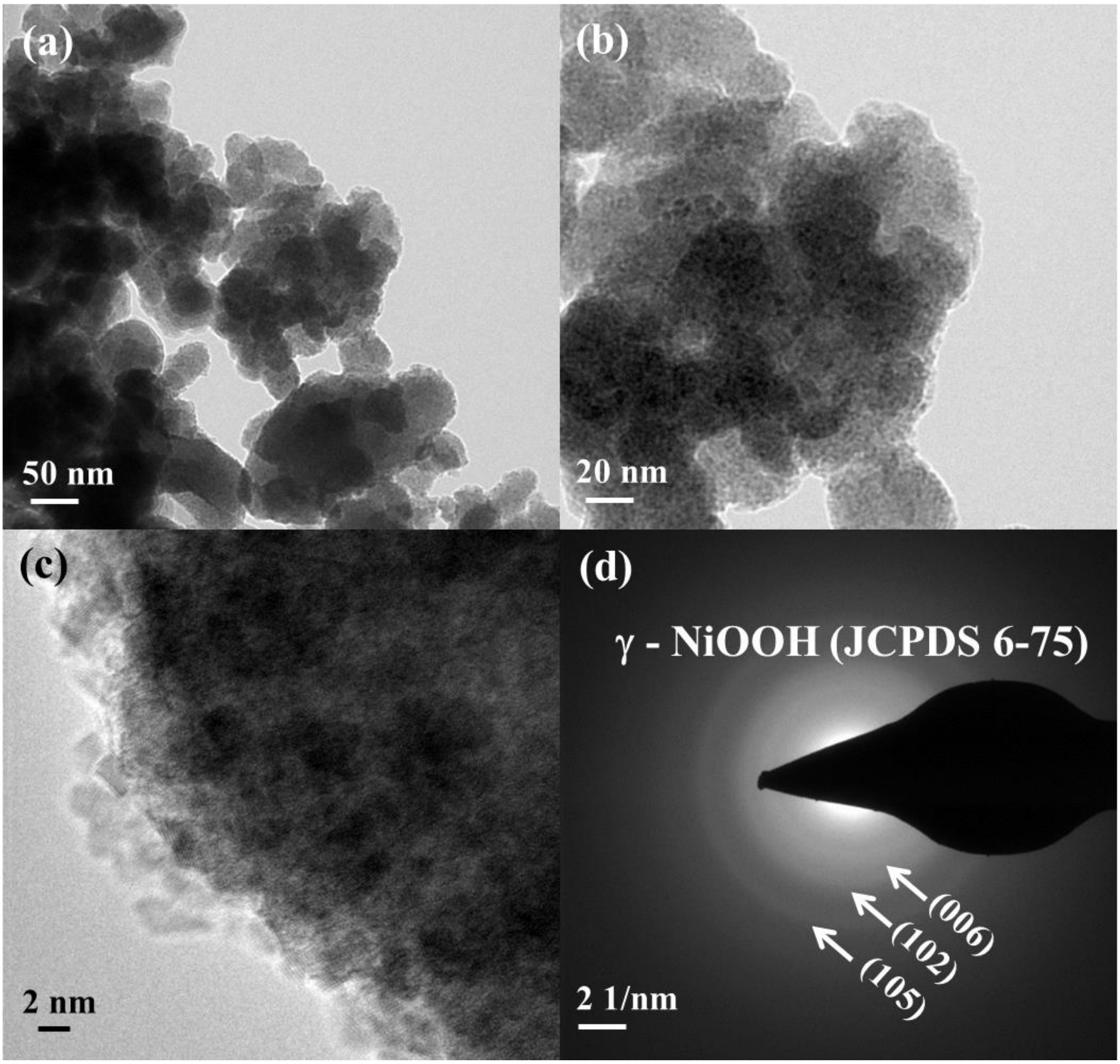

Figure S52. (a) TEM (a) HR-TEM images (b, c) and SAED (d) pattern of NiGe/FTO after OER CA (24 h). The nanoparticles were first separated from the FTO by ultrasonication and placed on the TEM grid. The TEM images (a) showed a complete transformation of initial NiGe into an agglomerated hollow nanostructure. The SAED pattern (d) produced weak diffraction rings of (006), (102), and (105) corresponding to the γ-NiOOH structure (JCPDS 6-75).

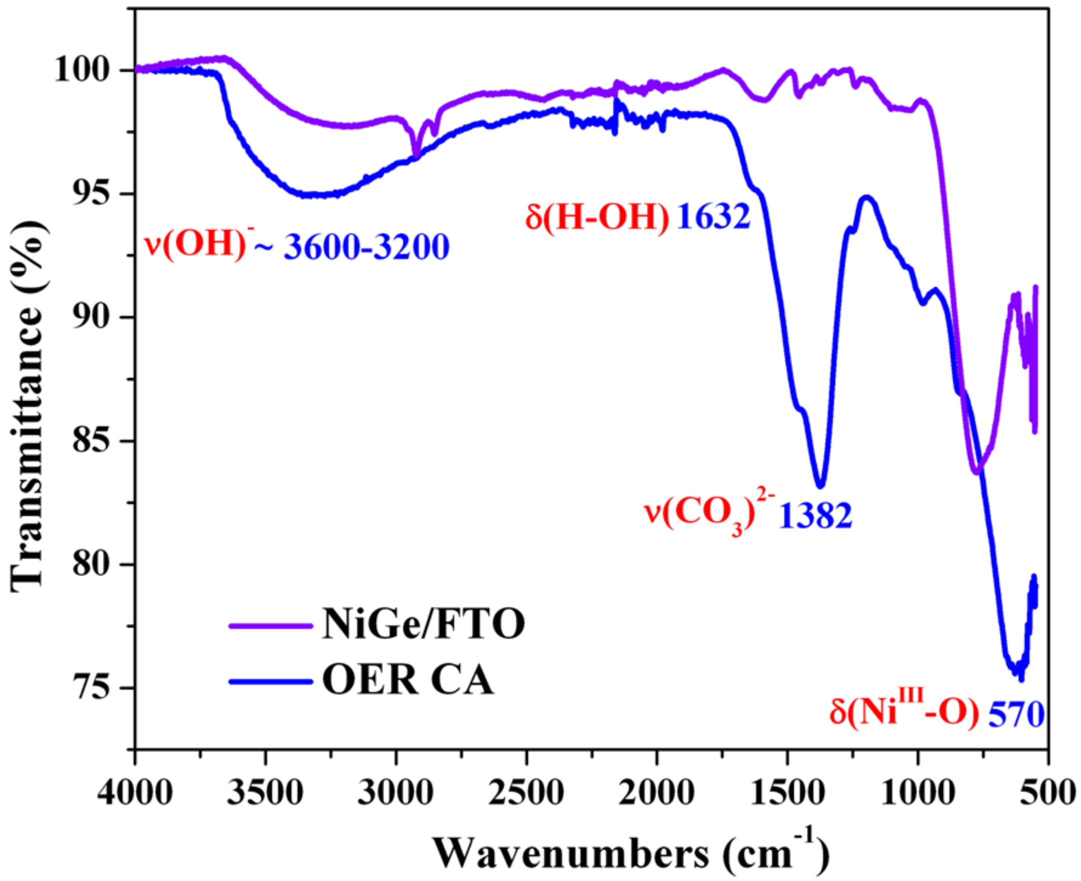

Figure S53. The FT-IR spectra of as-deposited NiGe/FTO (purple curve) and films after OER CA (24 h) (blue curve). After the OER, a complete transformation of the initial NiGe phase was observed with the appearance of new IR bands. The broad band around 3200-3600 cm^{-1} could be assigned to stretching vibrations of interlayer water molecules whereas the band at 1632 cm^{-1} could be ascribed to the bending vibration of H_2O and structural OH groups.[23] The band at 1382 cm^{-1} can be attributed to carbon-oxygen stretching vibrations in $(CO_3)^{2-}$ anions,[25] which apparently appeared because of the KOH electrolyte that consumes of CO_2 from ambient air in the form of dissolved carbonate.[26] Such intercalation carbonate anions between interlayer of γ-NiOOH has shown to be beneficial to enhance the OER activity.[26] Interestingly, the band at 570 cm^{-1} can directly be corroborated to Ni^{III}-O stretching vibrations of γ-NiOOH.[27] The FT-IR results clearly suggested the complete transformation of NiGe into γ-NiOOH, which is the active structure for alkaline OER catalysis.[28]

.

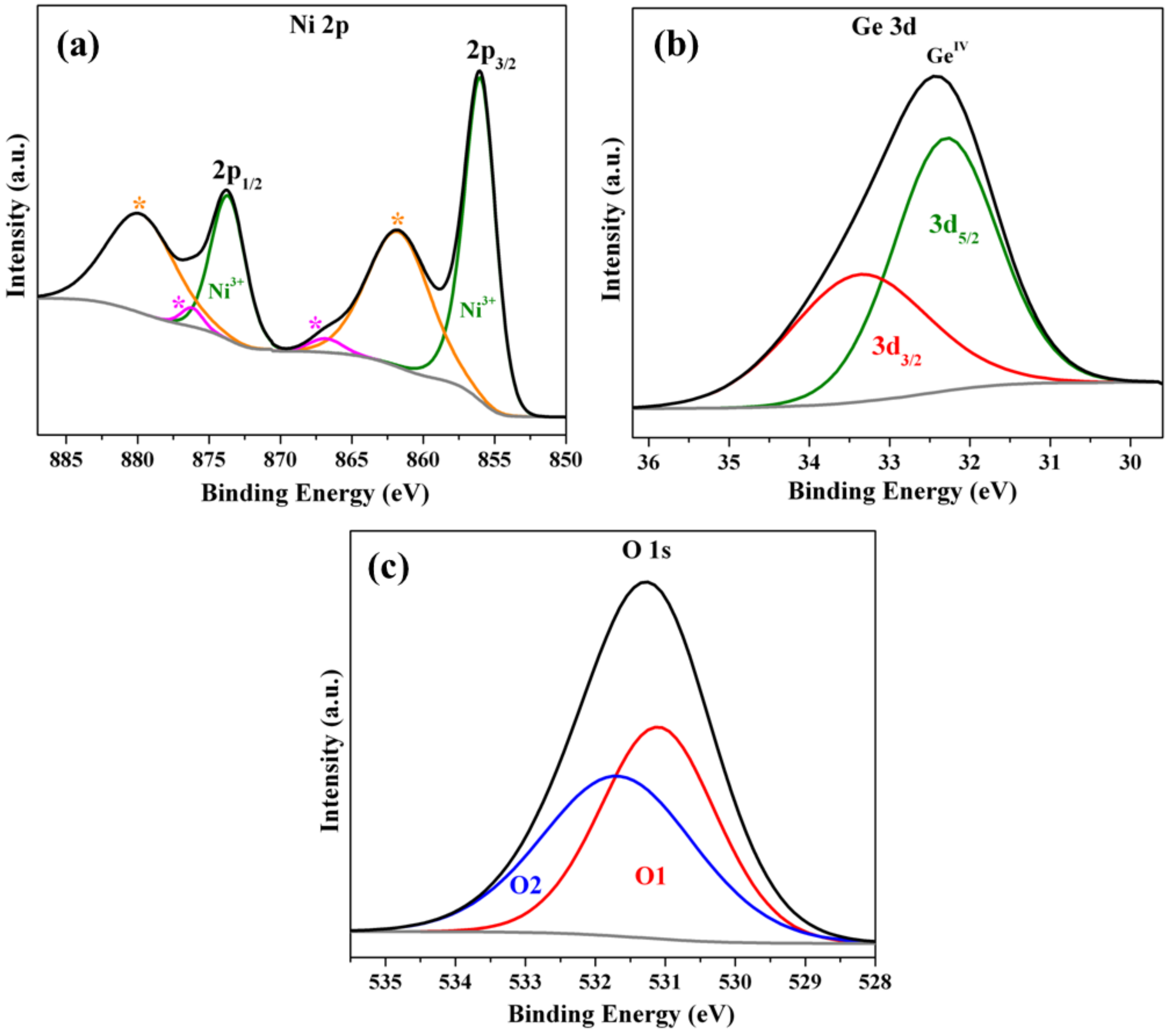

Figure S54. The high-resolution deconvoluted (a) Ni 2p (b) Ge 3d and (c) O 1s XPS spectra of NiGe/FTO film after OER CA (24 h). Both Ni $2p_{3/2}$ and Ni $2p_{1/2}$ spectrum displayed sharp peaks at the binding energy of 856 and 873.7 eV that can be ascribed to Ni^{III} in the structure, along with two satellite peaks, at 861.8 and 879.9 eV, due to multi-electron excitation.[27b,28] This showed that NiGe was converted to γ-NiOOH under alkaline electrochemical OER conditions, the binding values obtained here are typical to γ-NiOOH phase reported in the literature.[27,29] The Ge 3d exhibited peaks at 32.2 and 33.3 eV corresponding to Ge $2p_{5/2}$ and $2p_{3/2,}$ which is due to the adsorbed $Ge^{IV}O_2$ at the film surface.[20b,30] The O 1s spectrum was deconvoluted into two (O1 and O2) peaks, ~531 and 531.7 eV which is ascribed to γ-NiOOH.[31] From the XPS, it was confirmed that NiGe phase undergoes severe restructuring to form γ-NiOOH which is the active structure for electrocatalytic OER.[32] The surface atomic Ni:Ge composition of as-prepared material was ~1:0.09.

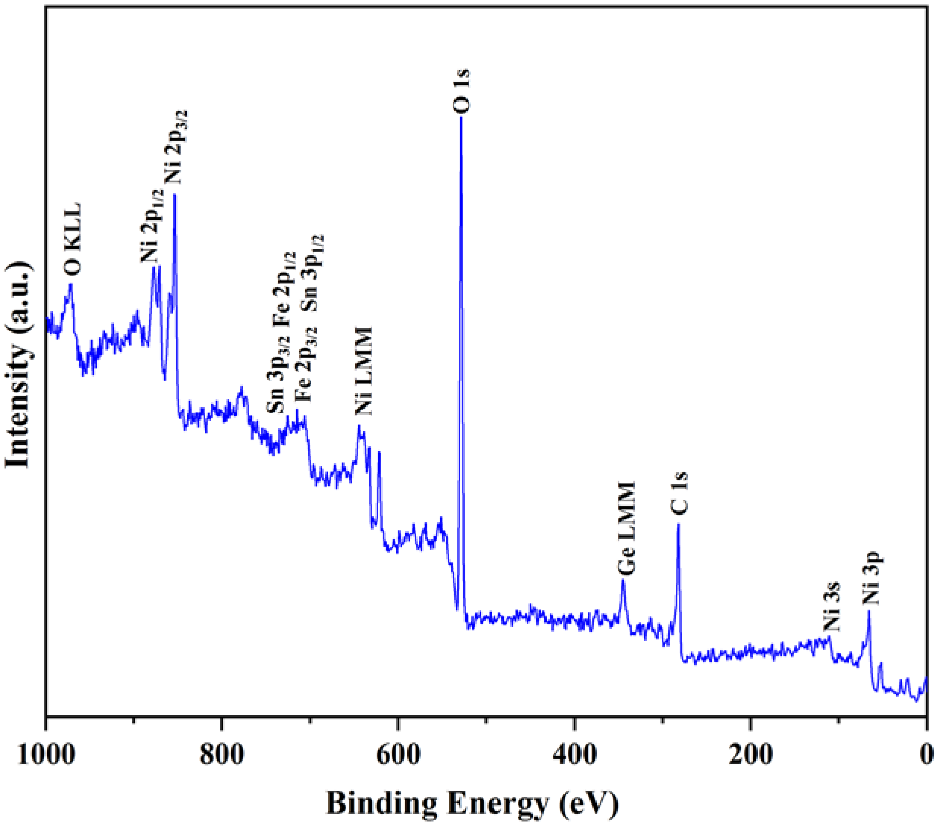

Figure S55. The XPS survey spectrum of NiGe/FTO film after OER CA (24 h). As the Fe 2p and Sn 3P peaks were heavily overlapped, the determination of Fe (from the electrolyte) was conducted from ICP-AES analysis that showed a negligible amount of Fe (<0.4±0.1%)

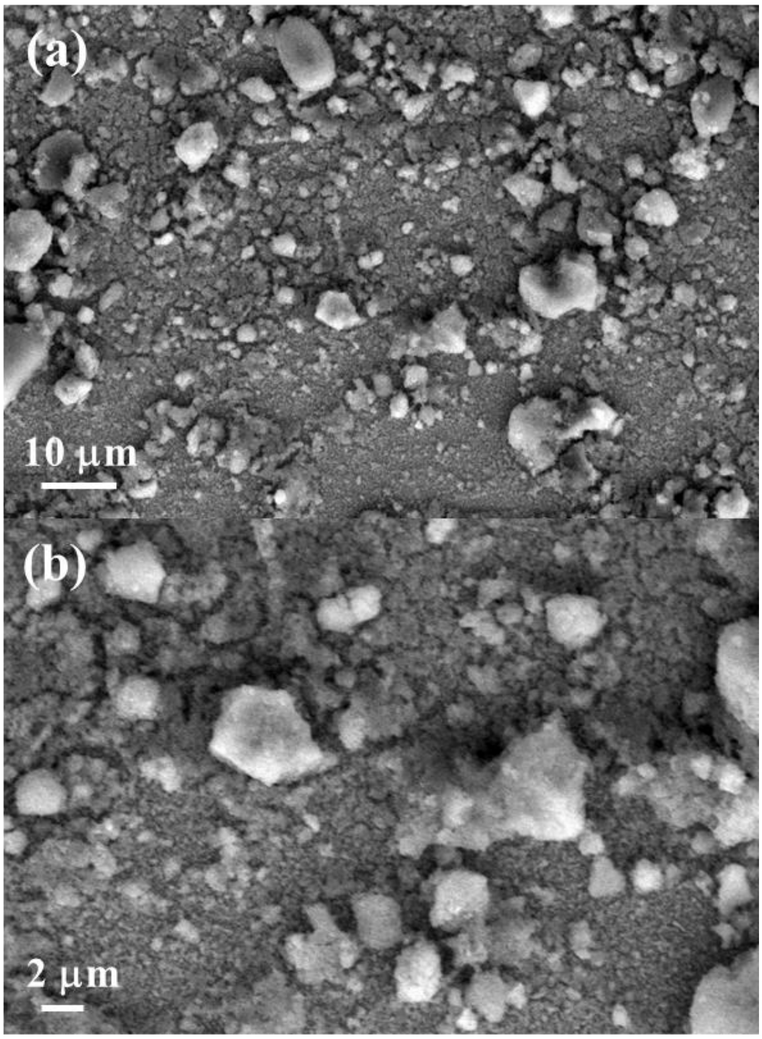

Figure S56. The SEM images (a-b) of NiGe/FTO films after OER CV (3 cycles) showing different magnifications. The nanoparticles displayed similar morphology as that of OER CA indicating the rapid and complete structural transformation of the initial NiGe phase.

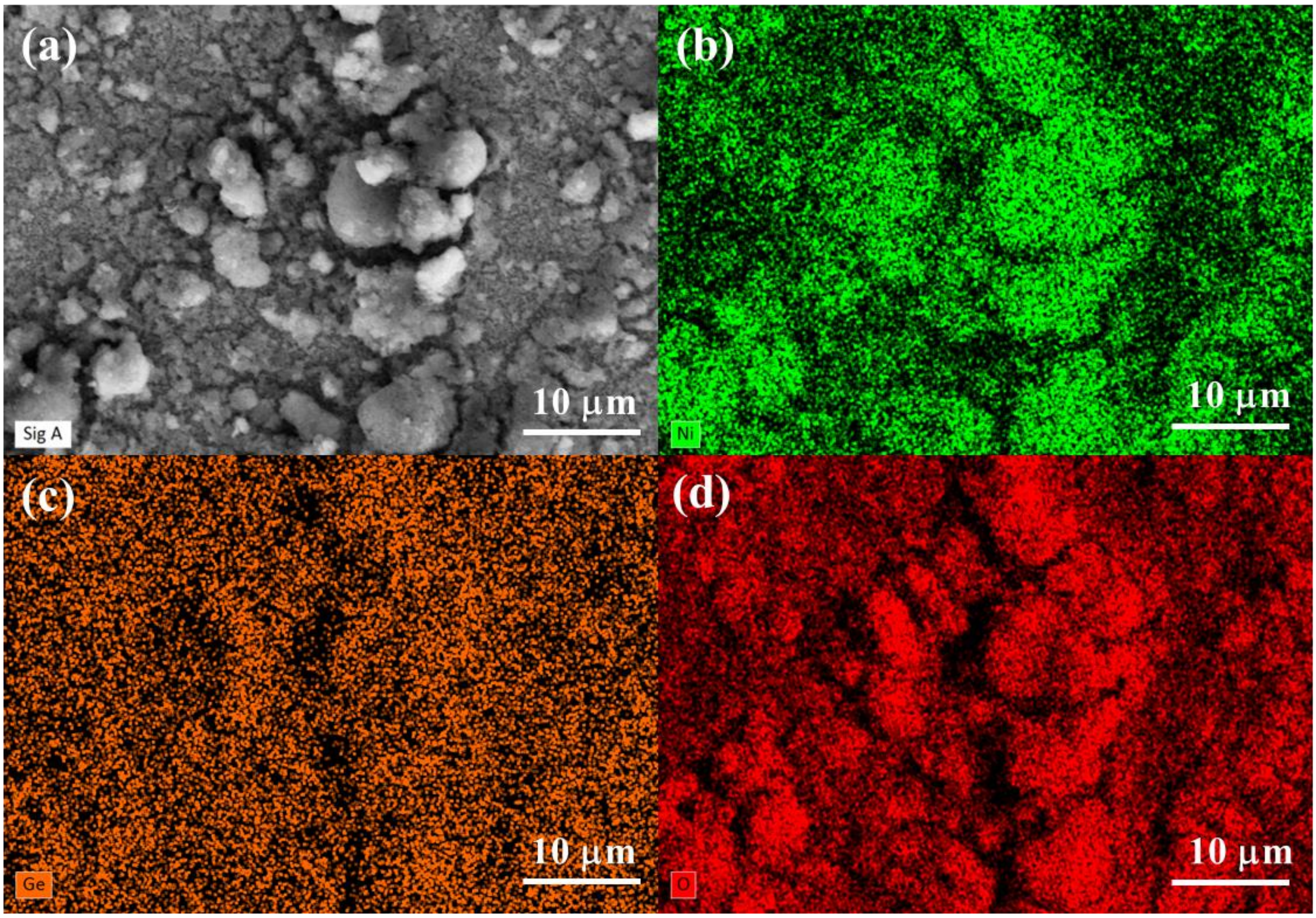

Figure S57. The SEM image (a) and the respective EDX mapping of NiGe after 3 cycles of OER CV (b-d). The spectra exhibited a similar change to that of films investigated after 24 h. Nickel (b) and oxygen (d) were homogeneously distributed within the particles and the germanium (c) atoms mostly depleted from the structure confirming complete conversion of NiGe under strongly alkaline OER conditions, as confirmed by the EDX spectrum (Figure S58). The composition obtained by EDX is listed in Table S5.

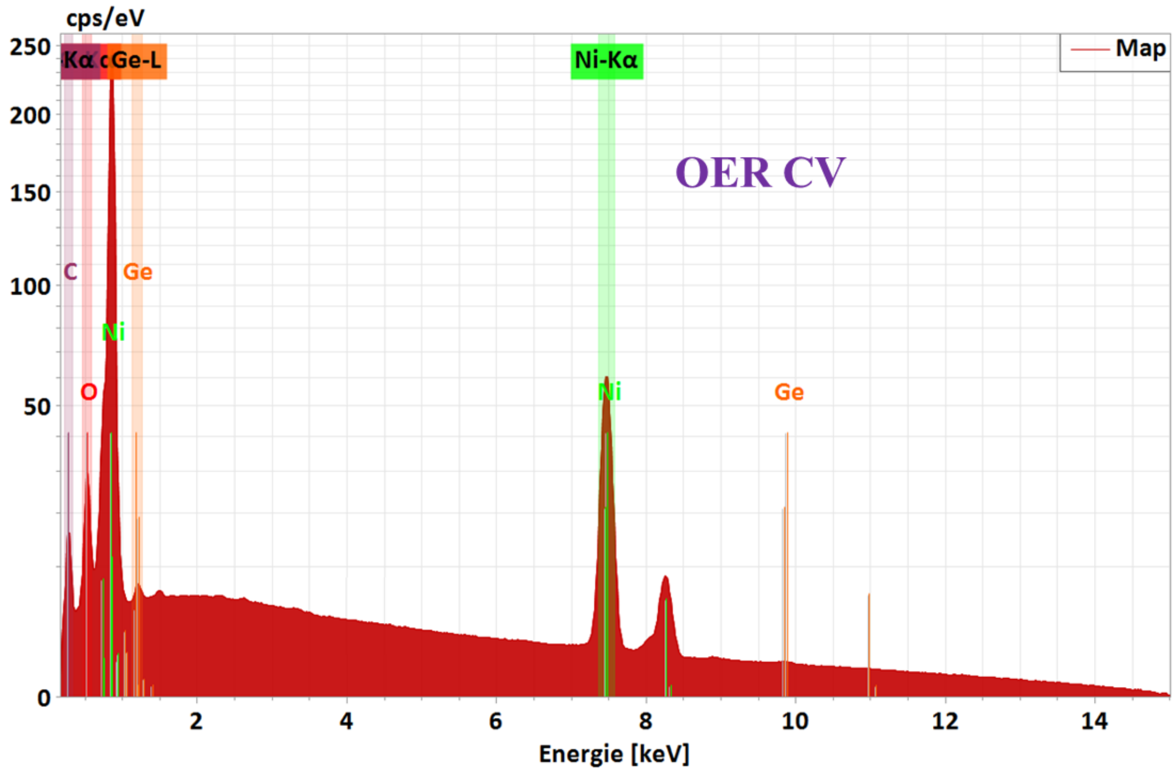

Figure S58. The EDX mapping spectrum of NiGe/FTO film after OER CV (3 cycles) indicating the rapid massive loss of Ge under OER.

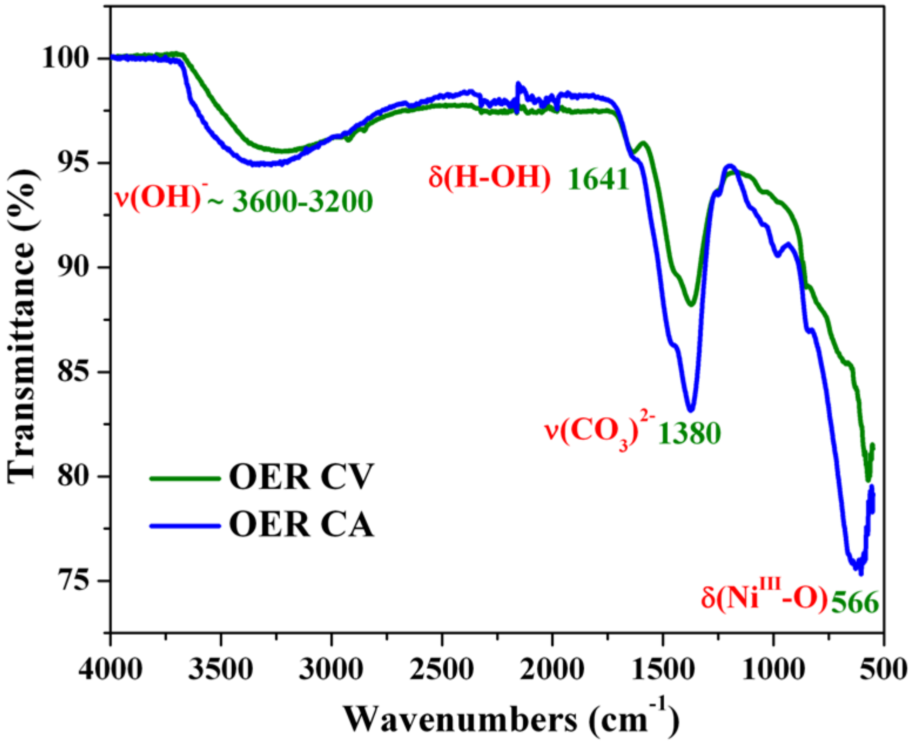

Figure S59. The FT-IR spectra of NiGe/FTO films after OER CV (green curve) and OER CA (blue curve). The IR spectra of both OER CV and OER CA were similar, suggesting that the fast transformation of NiGe to of NiGe into γ-NiOOH, which is the active structure for alkaline OER catalysis (for detailed band description refer Figure S53).

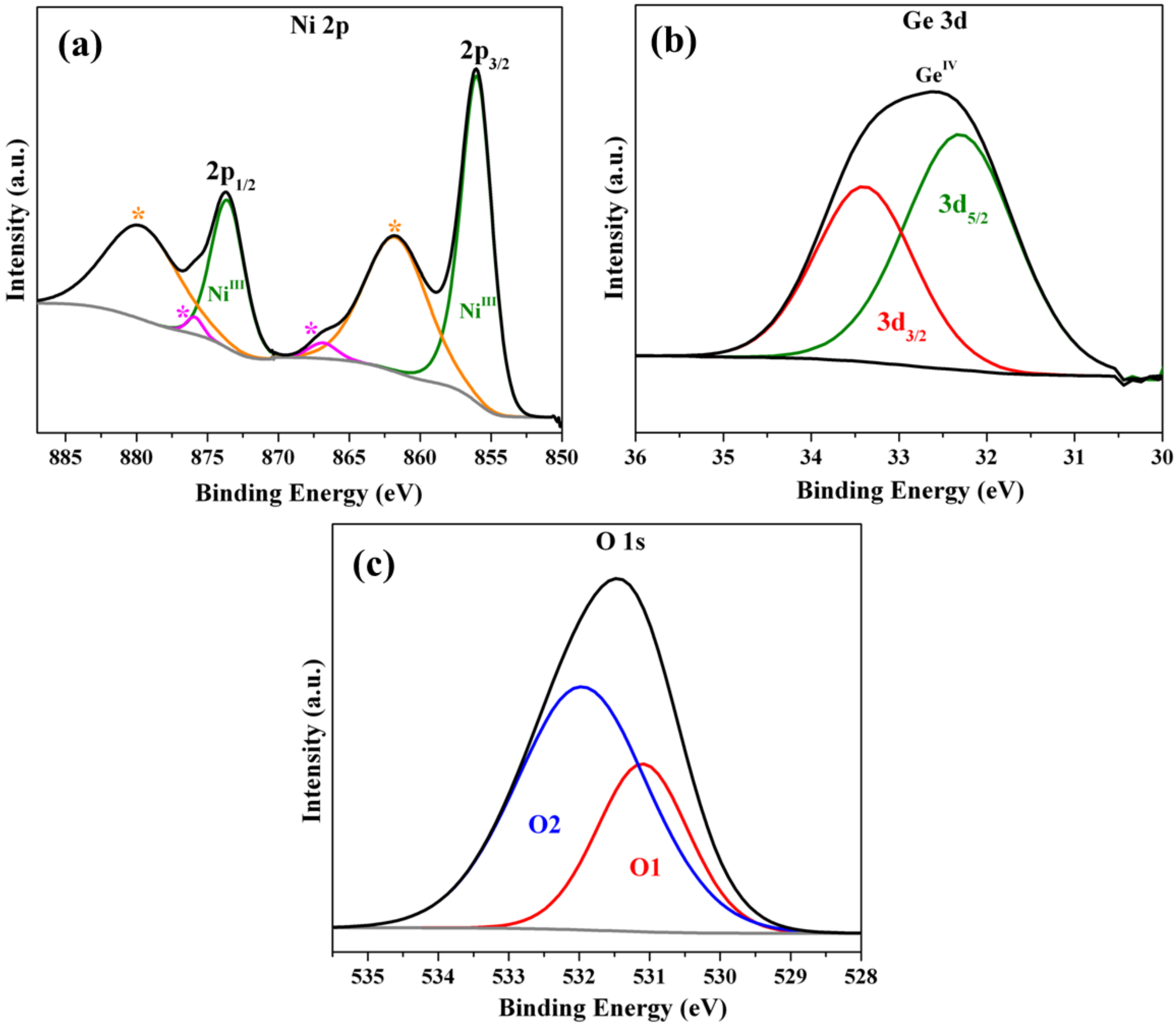

Figure S60. The high-resolution deconvoluted (a) Ni 2p (b) Ge 3d and (c) O 1s XPS spectra of NiGe/FTO film after OER CV (3 cycles). Both Ni $2p_{3/2}$ and Ni $2p_{1/2}$ spectrum displayed sharp peaks at the binding energy corresponding to Ni^{III} in the structure, along with two satellite peaks (see Figure S54). Similarly, the Ge $2p_{5/2}$ and $2p_{3/2}$ peaks showed the formation of adsorbed $Ge^{IV}O_2$ at the film surface. The O 1s spectrum was deconvoluted into two peaks (O1 and O2) that could be ascribed to γ-NiOOH.[31] The attained XPS spectra are very similar to that of OER CA (24 h) that demonstrates the rapid and severe restructuring of NiGe films to γ-NiOOH under alkaline OER conditions.[27,29]

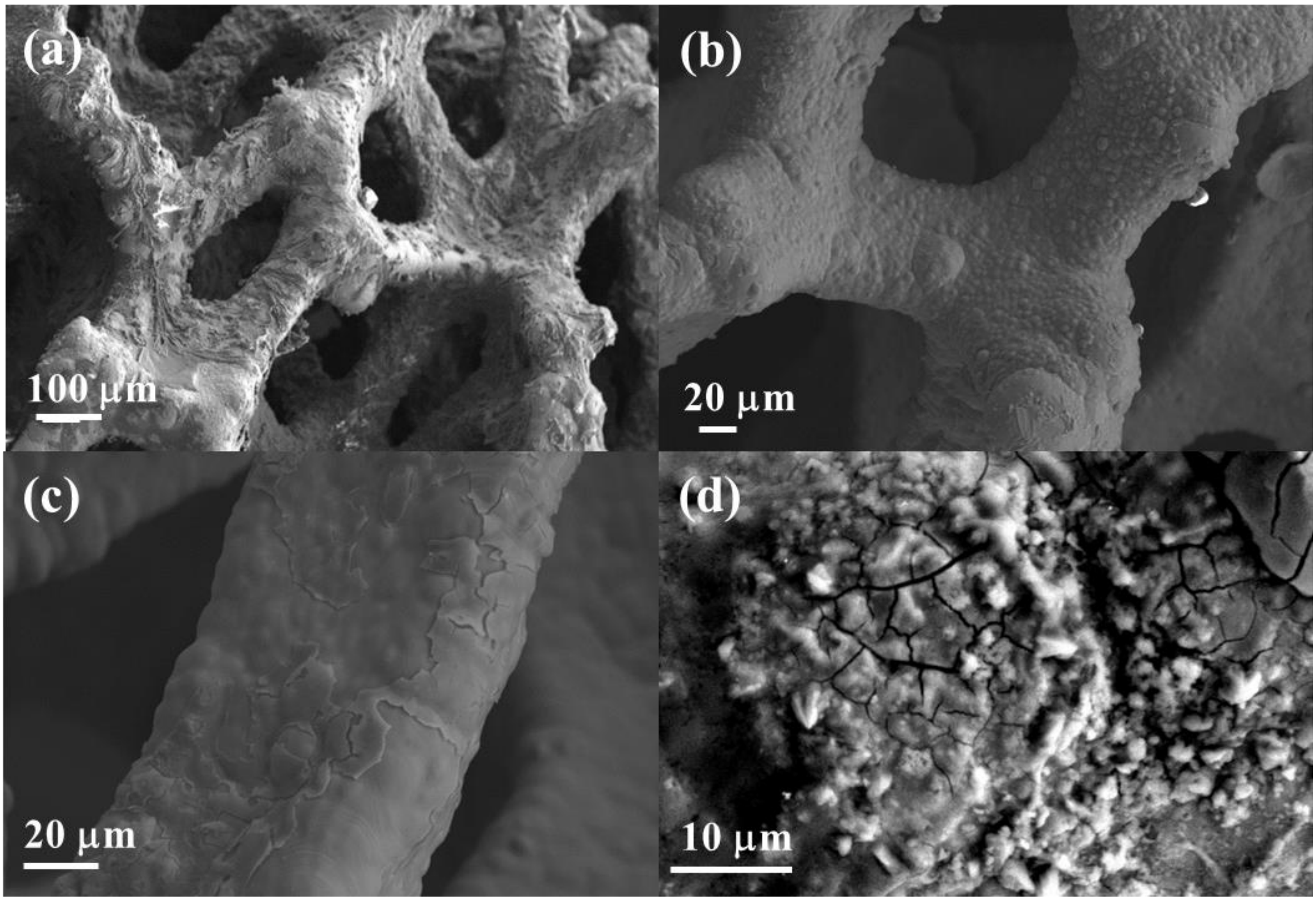

Figure S61. SEM images (a-d) of NiGe/NF films after OER CA of three-weeks (21 days) at various magnifications

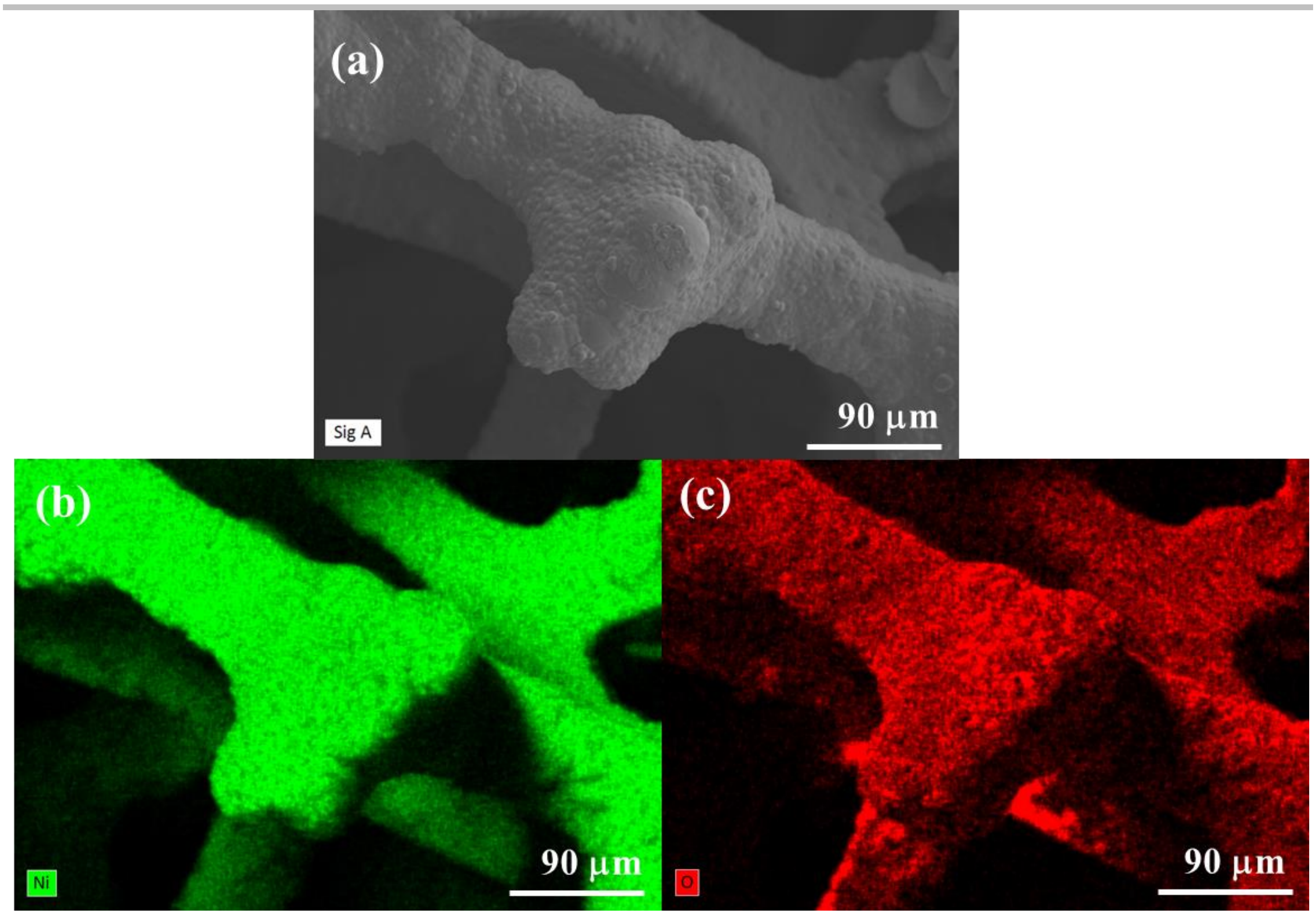

Figure S62. The SEM image (a) and the respective EDX mapping of NiGe/NF after three-weeks (b-c) of OER CA (21 days). Homogenous distribution of nickel (b) and oxygen (c) was evident and at the same time, the Ge was mostly disappeared from the structure (see Figure S63). This indicates that γ-NiOOH was formed after the corrosion-induced transformation of the NiGe nanoparticles under strongly alkaline conditions to enhance the catalytic OER activity.

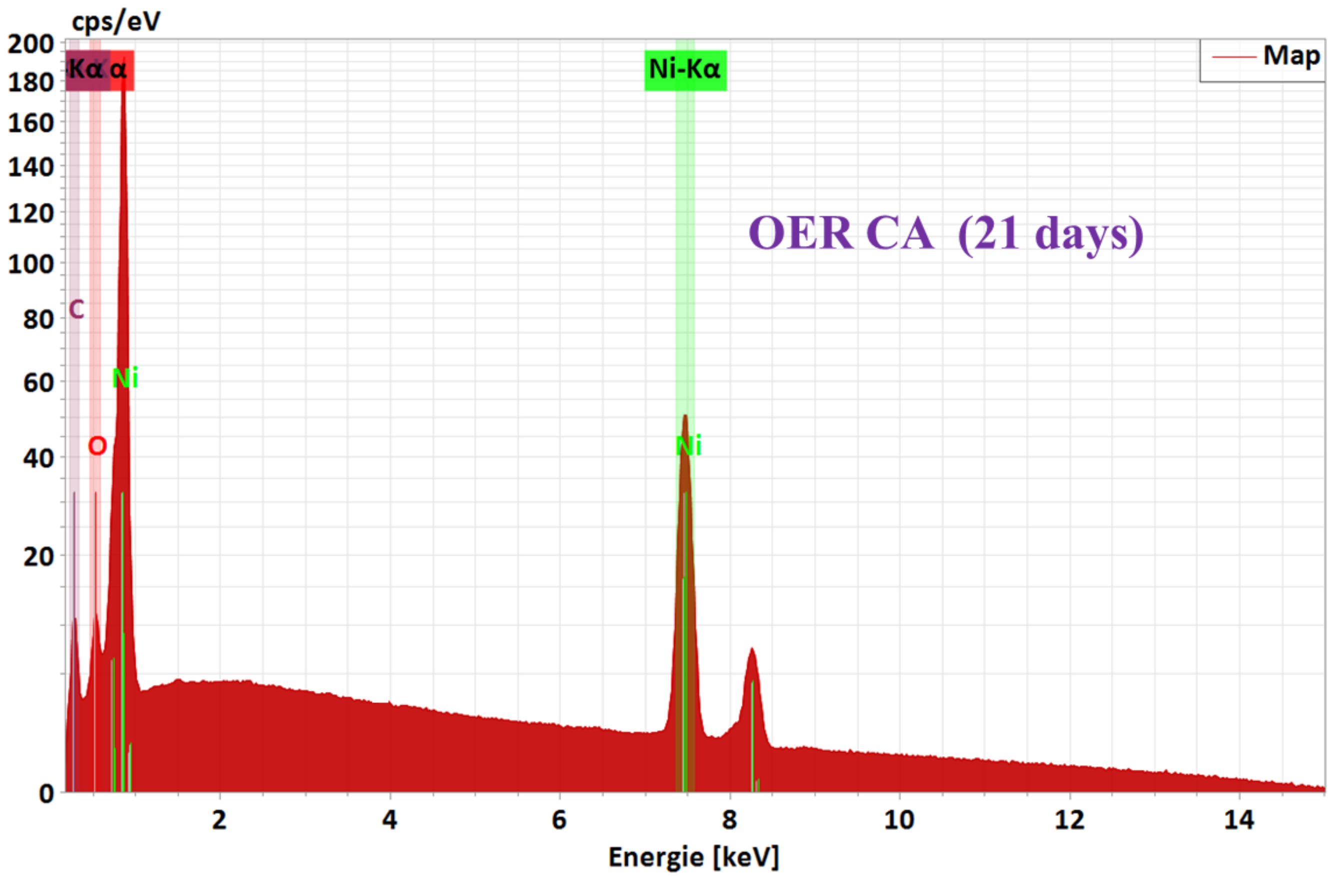

Figure S63. The EDX mapping spectrum of NiGe/NF after three-weeks (b-c) of OER CA (21 days) in 1 M aqueous solution suggesting the absence of Ge from the final active catalyst.

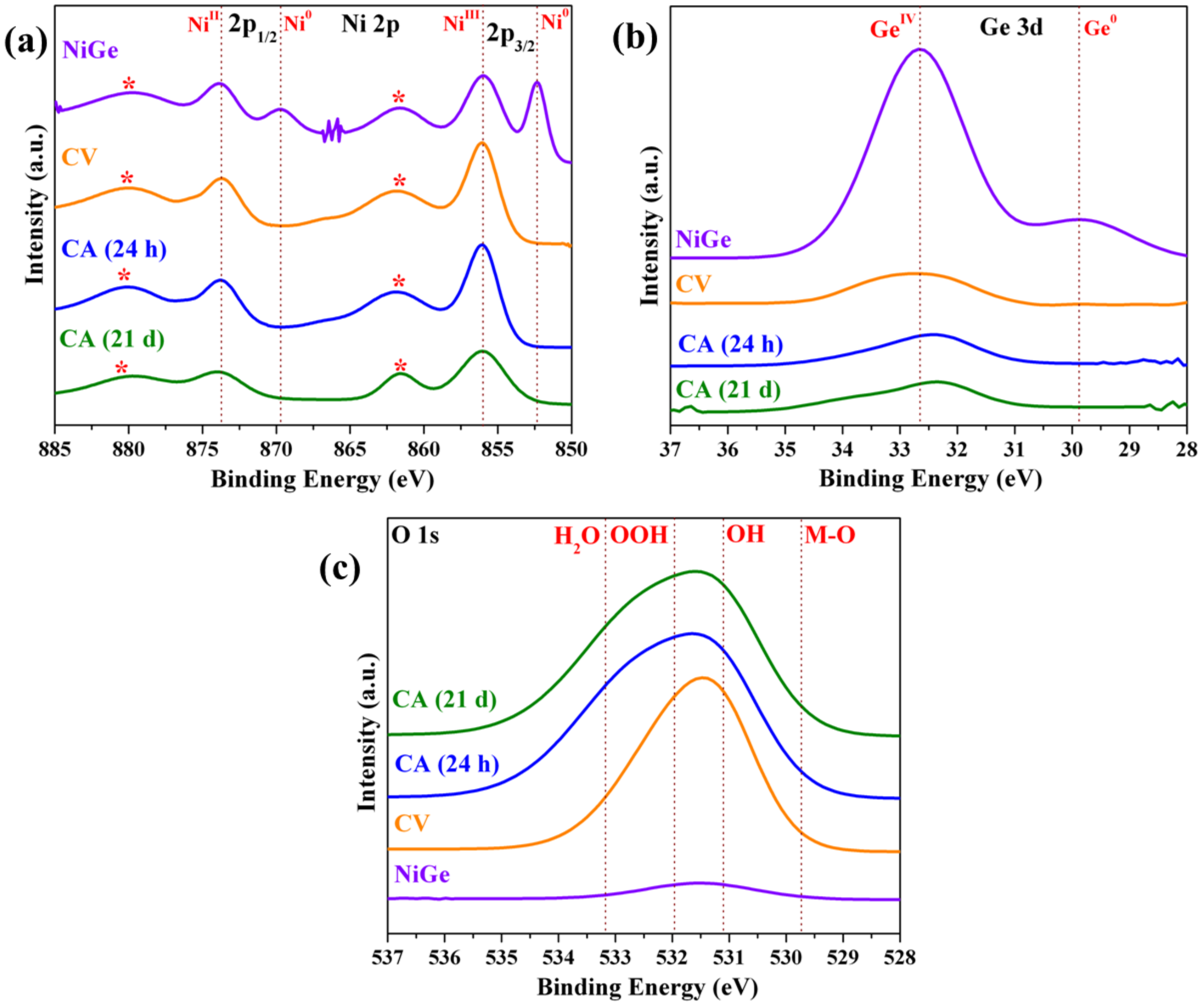

Figure S64. The comparison of the Ni 2p (a), Ge 3d (b), and O 1s (c) XPS spectra of as-deposited, films after OER CV (3 cycles), films after OER CA (24 h), and films after OER CA (21 days). It was clear from the Ni 2p spectra that the Ni^0 of NiGe was rapidly transformed into Ni^{III} in CV conditions and did not change further after 24 h and 21 days of OER CA.[18c] Similarly, Ge 3d spectra showed the fast depletion of Ge^0 in the CV, and only a peak for adsorbed $Ge^{IV}O_2$ was obtained in CA conditions.[18c,20b,30] Negligible oxygen was found in the as-deposited NiGe, however, under strongly alkaline OER conditions, peaks responsible for γ-NiOOH were observed.[31] The spectra obtained here is identical to that of γ-NiOOH that strongly suggested the electro conversion of NiGe initial phase to active γ-NiOOH structure by losing all of its Ge.[27,29]

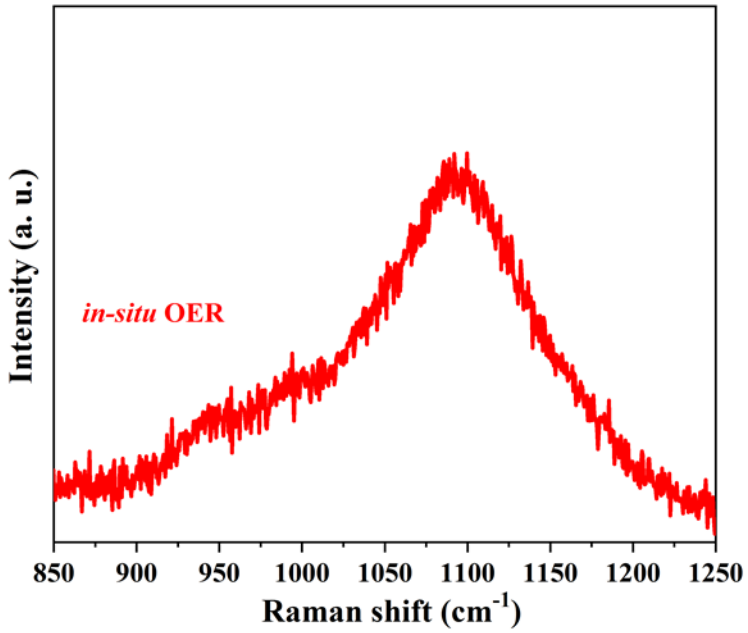

Figure S65. Quasi *in-situ* Raman spectra NiGe OER after an applied potential of 1.58 vs. RHE for 24 h in the wavenumber region of 800-1300 cm^{-1} suggesting Ni sites evolve O_2 through an active oxygen species (Ni-O-O^-).[33]

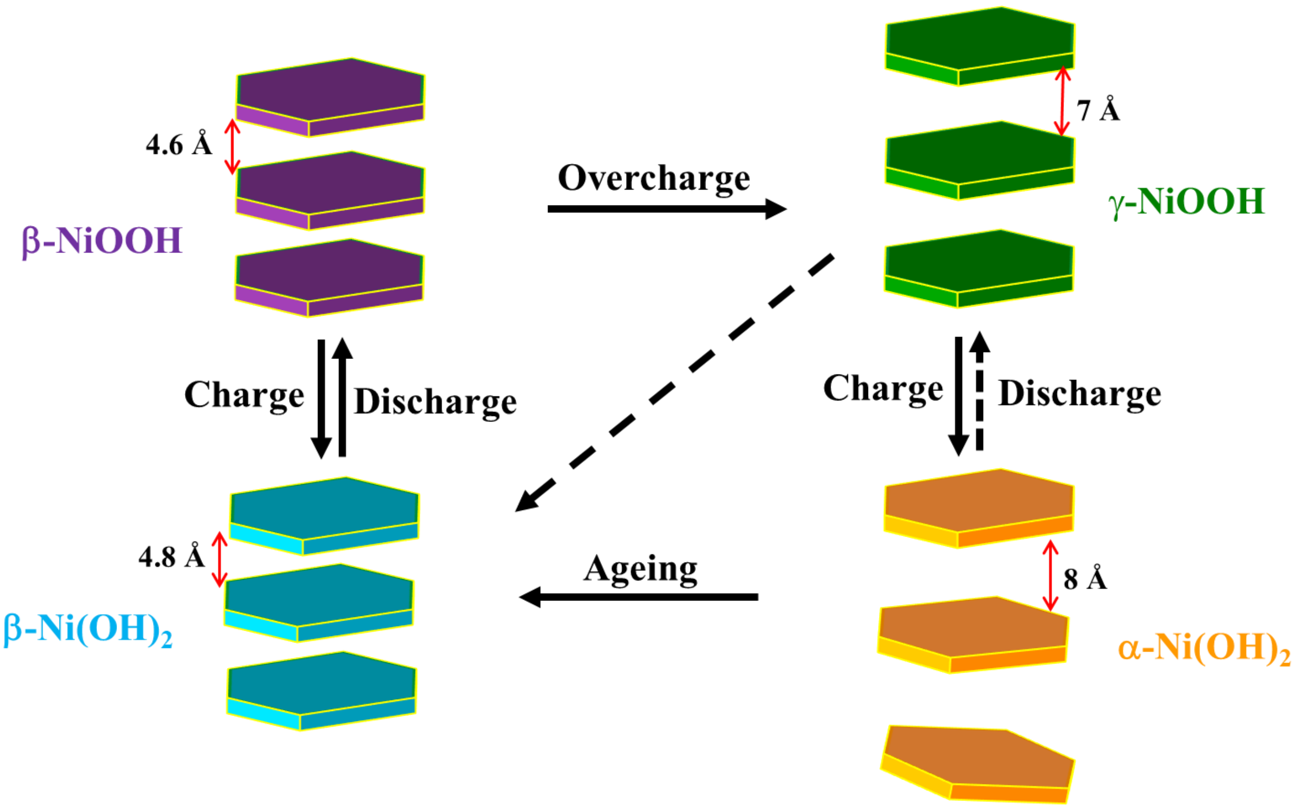

Figure S66. Bode's diagram representing different variation of nickel (oxy)hydroxides and possible phase transformation within them.[34]

Table S11. The comparison of OER (*η*) overpotentials, Stabii of NiGe with other established selected Ni-based catalysts in aqueous 1 M KOH.

Catalyst	*j* (mA·cm^{-2})	Substrate	Stability (h)	*η* (mV)	Loading (mgcm^{-2})	Ref
**NiGe**	**10**	**NF**	**505**	**228±3**	**1**	**This work**
**NiGe**	**10**	**FTO**	**24**	**322±2**	**0.4**	**This work**
NiFeOOH	10	NiFe	2	240	-	[35]
NiO_x-Fe	10	NF	18	215	0.014	[36]
NiFe LDH	10	NF	13	300	1.0	[37]
$Ni_3Fe_{0.5}V_{0.5}$	10	CFP	60	200	-	[38]
NiFe LDH	10	GC	1	210	1.0	[39]
Ni_2P	10	NF	12	240	3.0	[40]
$Ni_{12}P_5$	10	NF	12	260	3.0	[40]
NiFe LDH	10	HOPG	5	260	0.040	[41]
Ni_3S_2	10	NF	200	260	1.6	[42]
NiFe-LDH	10	NF	3	240	0.19	[43]
Ni_2P	10	FTO	16	330	1.0	[40]
$Ni_{12}P_5$	10	FTO	16	295	1.0	[40]
$FeNiO_xH_y$	10	NF	50	206	1.4	[44]
CuO@$NiFeOH_x$	10	Cu	16	230	2.2	[45]
NiFe-MOF	10	CFP	100	275	1.0	[46]
NiFe-LDH	10	CW	-	260	0.9	[47]
NiFe-LDH	10	NW	1.5	300	0.285	[48]
NiFe alloy	10	GC	2	298	0.22	[49]
NiFe-MOF	10	GC	5.5	230	-	[50]
NiFeTiOOH	10	GC	24	400	0.015	[51]
NiFeCo-LDH	10	CFP	10	288	0.532	[52]
NiFe alloy	10	GC	20	246	0.300	[53]
CNS-NiFe	10	Cu_2O/Cu	24	248	-	[54]
NiFe-LDH	10	CB	6	236	0.25	[55]
NiFe/$CoFe_2O_4$/Co_3S_4	10	CFP	48	233	1.0	[56]
$Ni(OH)_2(CO_3)$-Fe^{2+}	10	NF	36	277	-	[57]
$NiFeNiFe_2O_4$	10	NF	15	316	0.57	[58]
$NiFe_xSn$@$NiFe(OH)_x$	10	CFC	11	260	0.048	[59]
Co-NiFe-LDH	10	GC	20	278	-	[60]
$NiFe(Co_3)^{2-}$-LDH	10	NF	20	228	4.5	[61]
NiFe	10	NF	30	270	2.0	[62]
$NiFe_2O_4$/NFM	10	Fe wire	10	234	-	[63]
NiFe15	10	NiFe sponge	24	280	0.7	[64]
NiO/C@NiFe-LDH	10		10	299	-	[65]
NiFe-LDH@Ni_3S_2	10	NF	6	271	-	[66]
NiFe-HC	10	GC	12	330		[67]
Ni_1Fe_{10}-LDH@Ni_3S_2	10	NF	12	230		[68]
$NiFeS_2$	10	GC	24	230	0.18	[69]
NiFeS	10	GC	6	286	0.25	[70]
$Ni_{0.7}Fe_{0.3}S_{2+y}$	10	NF	24	210	1-3	[71]
$Ni_{0.7}Fe_{0.3}S_2$	10	NF	14	198	3	[72]
$Ni_xFe_{1-x}Se_2$	10	GC	24	195	-	[73]

GC = glassy carbon, CFP = carbon fiber paper, Au = gold, CC =carbon cloth, FTO = fluorine doped tin oxide, HOPG = highly-ordered pyrolytic graphite, NF = nickel foam, CW= carbon wire; CB = carbon black

References

[1] a) J. S. Nowick, P. Ballester, F. Ebmeyer, J. Rebek, *J. Am. Chem. Soc.* **1990**, *112*, 8902-8906; b) A. Buhling, P. C. J. Kamer, P. vanLeeuwen, J. W. Elgersma, K. Goubitz, J. Fraanje, *Organometallics* **1997**, *16*, 3027-3037.

[2] S. Nagendran, S. S. Sen, H. W. Roesky, D. Koley, H. Grubmuller, A. Pal, R. Herbst-Irmer, *Organometallics* **2008**, *27*, 5459-5463.

[3] G. M. Sheldrick, *SHELX-97 Program for Crystal Structure Determination* **1997**, Universität Göttingen (Germany)

[4] Y. P. Vinichenko, E. N. Sidorova, *J. Phys.: Conference Series* **2016**, *741* 012194.

[5] J. Yang, H. W. Liu, W. N. Martens, R. L. Frost, *J. Phys. Chem. C* **2010**, *114*, 111-119.

[6] C. Panda, P. W. Menezes, M. Zheng, S. Orthmann, M. Driess, *ACS Energy Lett.* **2019**, *4*, 747-754.

[7] a) L. Besra, M. Liu, *Prog. Mater. Sci.* **2007**, *52*, 1-61; b) F. Bozza, R. Polini, E. Traversa, *Fuel Cells* **2008**, *8*, 344-350; c) P. W. Menezes, C. Panda, S. Garai, C. Walter, A. Guiet, M. Driess, *Angew. Chem. -Int. Ed.* **2018**, *57*, 15237-15242; d) J. Pfrommer, A. Azarpira, A. Steigert, K. Olech, P. W. Menezes, R. F. Duarte, X. X. Liao, R. G. Wilks, M. Bar, T. Schedel-Niedrig, M. Driess, *ChemCatChem* **2017**, *9*, 672-676.

[8] a) Y. Yang, J. X. Huang, J. Zeng, J. Xiong, J. B. Zhao, *ACS Appl. Mater. Interfaces* **2017**, *9*, 32801-32811; b) Y. Yang, J. Q. Li, D. Q. Chen, T. Fu, D. Sun, J. B. Zhao, *ChemElectroChem* **2016**, *3*, 757-763.

[9] G. Inzelt, A. Lewenstam, F. Scholzm, *Handbook of Reference Electrodes*, Springer-Verlag Berlin Heidelberg **2013**.

[10] S. Anantharaj, S. R. Ede, K. Karthick, S. S. Sankar, K. Sangeetha, P. E. Karthik, S. Kundu, *Energy Environ. Sci.* **2018**, *11*, 744-771.

[11] a) C. C. L. McCrory, S. Jung, I. M. Ferrer, S. M. Chatman, J. C. Peters, T. F. Jaramillo, *J. Am. Chem. Soc.* **2015**, *137*, 4347-4357; b) C. C. L. McCrory, S. H. Jung, J. C. Peters, T. F. Jaramillo, *J. Am. Chem. Soc.* **2013**, *135*, 16977-16987.

[12] a) Y. Zhang, X. L. Fan, J. H. Jian, D. S. Yu, Z. S. Zhang, L. M. Dai, *Energy Environ. Sci.* **2017**, *10*, 2312-2317; b) M. X. Li, H. Y. Wang, W. D. Zhu, W. M. Li, C. Wang, X. F. Lu, *Advanced Science* **2020**, *7*.

[13] P. W. Menezes, A. Indra, I. Zaharieva, C. Walter, S. Loos, S. Hoffmann, R. Schlo¨gl, H. Dau, M. Driess, *Energy Environ. Sci.* **2018**, *12*, 988-999

[14] B. De Schutter, W. Devulder, A. Schrauwen, K. van Stiphout, T. Perkisas, S. Bals, A. Vantomme, C. Detavernier, *Microelectron. Eng.* **2014**, *120*, 168-173.

[15] H. Pfisterer, K. Schubert, *Z. Metallkd.* **1950**, *41*, 358-367.

[16] a) Z. Gercsi, K. G. Sandeman, *Phys. Rev. B* **2010**, *81*; b) M. K. Niranjan, L. Kleinman, A. A. Demkov, *Phys Rev. B* **2007**, *75*, 10.

[17] P. W. Menezes, C. Walter, J. N. Hausmann, R. Beltran-Suito, C. Schlesiger, S. Praetz, V. Y. Verchenko, A. V. Shevelkov, M. Driess, *Angew. Chem. Int. Ed.* **2019**, *58*, 16569-16574.

[18] a) A. E. Bocirnea, L. C. Tanase, R. M. Costescu, N. G. Apostol, C. M. Teodorescu, *Appl. Sur. Sci.* **2017**, *424*, 269-274; b) T. Grzela, G. Capellini, W. Koczorowski, M. A. Schubert, R. Czajka, N. J. Curson, I. Heidmann, T. Schmidt, J. Falta, T. Schroeder, *Nanotechnology* **2015**, *26*, 385701; c) J. Y. Chen, S. L. Jheng, H. Y. Tuan, *Nanoscale* **2018**, *10*, 11072-11078.

[19] a) A. Davidson, J. F. Tempere, M. Che, H. Roulet, G. Dufour, *J. Phys. Chem.* **1996**, *100*, 4919-4929; b) H. W. Nesbitt, D. Legrand, G. M. Bancroft, *Phys. Chem. Min.* **2000**, *27*, 357-366; c) P. W. Menezes, C. Panda, S. Loos, F. Bunschei-Bruns, C. Walter, M. Schwarze, X. H. Deng, H. Dau, M. Driess, *Energy Environ. Sci.* **2018**, *11*, 1287-1298.

[20] a) N. M. Bom, G. V. Soares, S. Hartmann, A. Bordin, C. Radtke, *Appl. Phys. Lett.* **2014**, *105*; b) N. Tabet, M. Faiz, N. M. Hamdan, Z. Hussain, *Sur. Sci.* **2003**, *523*, 68-72.
[21] C. Wei, R. R. Rao, J. Peng, B. Huang, I. E. L. Stephens, M. Risch, Z. J. Xu, Y. Shao-Horn, *Adv. Mater.* **2019**, *31*, 1806296.
[22] H. Dau, C. Limberg, T. Reier, M. Risch, S. Roggan, P. Strasser, *ChemCatChem* **2010**, *2*, 724-761.
[23] a) J. Park, Y. J. Sa, H. Baik, T. Kwon, S. H. Joo, K. Lee, *ACS Nano* **2017**, *11*, 5500-5509; b) E. Antolini, *ACS Catal.* **2014**, *4*, 1426-1440; c) M. S. Burke, M. G. Kast, L. Trotochaud, A. M. Smith, S. W. Boettcher, *J. Am. Chem. Soc.* **2015**, *137*, 3638-3648; d) R. L. Doyle, I. J. Godwin, M. P. Brandon, M. E. G. Lyons, *Phys. Chem. Chem. Phys.* **2013**, *15*, 13737-13783.
[24] a) Y. P. Liu, X. Liang, L. Gu, Y. Zhang, G. D. Li, X. X. Zou, J. S. Chen, *Nat. Commun.* **2018**, *9*, 2609; b) S. Yao, V. Forstner, P. W. Menezes, C. Panda, S. Mebs, E. M. Zolnhofer, M. E. Miehlich, T. Szilvasi, N. A. Kumar, M. Haumann, K. Meyer, H. Grutzmacher, M. Driess, *Chem.l Sci.* **2018**, *9*, 8590-8597; c) T. Shinagawa, A. T. Garcia-Esparza, K. Takanabe, *Sci. Rep.* **2015**, *5*, 13801.
[25] A. A. Lobinsky, V. P. Tolstoy, I. A. Kodinzev, *Nanosystems-Phys. Chem. Maths.* **2018**, *9*, 669-675.
[26] B. M. Hunter, W. Hieringer, J. R. Winkler, H. B. Gray, A. M. Muller, *Energy Environ. Sci.* **2016**, *9*, 1734-1743.
[27] a) C. W. Hu, Y. Yamada, K. Yoshimura, *Solar Energy Materials and Solar Cells* **2018**, *177*, 120-127; b) C. W. Hu, Y. Yamada, K. Yoshimura, *J. Mater. Chem. C* **2016**, *4*, 5390-5397.
[28] Z. H. Yan, H. M. Sun, X. Chen, H. H. Liu, Y. R. Zhao, H. X. Li, W. Xie, F. Y. Cheng, J. Chen, *Nature Communications* **2018**, *9*, 2373.
[29] a) M. C. Biesinger, B. P. Payne, L. W. M. Lau, A. Gerson, R. S. C. Smart, *Sur. Interf. Anal.* **2009**, *41*, 324-332; b) N. Weidler, J. Schuch, F. Knaus, P. Stenner, S. Hoch, A. Maljusch, R. Schafer, B. Kaiser, W. Jaegermann, *J. Phys. Chem. C* **2017**, *121*, 6455-6463.
[30] K. Prabhakaran, T. Ogino, *Sur. Sci.* **1995**, *325*, 263-271.
[31] E. L. Ratcliff, J. Meyer, K. X. Steirer, A. Garcia, J. J. Berry, D. S. Ginley, D. C. Olson, A. Kahn, N. R. Armstrong, *Chem. Mater.* **2011**, *23*, 4988-5000.
[32] D. J. Zhou, S. Y. Wang, Y. Jia, X. Y. Xiong, H. B. Yang, S. Liu, J. L. Tang, J. M. Zhang, D. Liu, L. R. Zheng, Y. Kuang, X. M. Sun, B. Liu, *Angew. Chem. Int. Ed.* **2019**, *58*, 736-740.
[33] a) A. C. Garcia, T. Touzalin, C. Nieuwland, N. Perini, M. T. M. Koper, *Angew. Chem. Int. Ed.* **2019**, *58*, 12999-13003; b) O. Diaz-Morales, D. Ferrus-Suspedra, M. T. M. Koper, *Chem. Sci.* **2016**, *7*, 2639-2645; c) K. M. Cole, D. W. Kirk, S. J. Thorpe, *J. Electrochem. Soc.* **2018**, *165*, J3122-J3129; d) S. Lee, K. Banjac, M. Lingenfelder, X. L. Hu, *Angew. Chem. Int. Ed.* **2019**, *58*, 10295-10299; e) S. Lee, L. C. Bai, X. L. Hu, *Angew. Chem. Int. Ed.* **2020**, *59*, 8072-8077.
[34] a) S. Jahangiri, N. J. Mosey, *Phy. Chem. Chem. Phys.* **2018**, *20*, 11444-11453; b) A. Van der Ven, D. Morgan, Y. S. Meng, G. Ceder, *J. Electrochem. Soc.* **2006**, *153*, A210-A215.
[35] S. Loos, I. Zaharieva, P. Chernev, A. Lissner, H. Dau, *ChemSusChem* **2019**, *12*, 1966-1976.
[36] F. Song, M. M. Busch, B. Lassalle-Kaiser, C.-S. Hsu, E. Petkucheva, M. Bensimon, H. M. Chen, C. Corminboeuf, X. Hu, *ACS Cent. Sci.* **2019**, *5*, 558-568.
[37] F. Song, X. Hu, *Nat. Commun.* **2014**, *5* 4477.
[38] J. Jiang, F. Sun, S. Zhou, W. Hu, H. Zhang, J. Dong, Z. Jiang, J. Zhao, J. Li, W. Yan, M. Wang, *Nat. Commun.* **2018**, *9*, 1038.

[39] W. Ma, R. Ma, C. Wang, J. Liang, X. Liu, K. Zhou, T. Sasaki, *ACS Nano* **2015**, *9*, 1977-1984.
[40] P. W. Menezes, A. Indra, C. Das, C. Walter, C. Gobel, V. Gutkin, D. Schmeisser, M. Driess, *ACS Catal.* **2017**, *7*, 103-109.
[41] B. M. Hunter, J. D. Blakemore, M. Deimund, H. B. Gray, J. R. Winkler, A. M. Muller, *J. Am. Chem. Soc.* **2014**, *136*, 13118-13121.
[42] L. L. Feng, G. T. Yu, Y. Y. Wu, G. D. Li, H. Li, Y. H. Sun, T. Asefa, W. Chen, X. X. Zou, *J. Am. Chem. Soc.* **2015**, *137*, 14023-14026.
[43] K. Zhang, W. H. Wang, L. Kuai, B. Y. Geng, *Electrochim. Acta* **2017**, *225*, 303-309.
[44] X. J. Wu, Y. M. Zhao, T. Y. Xing, P. L. Zhang, F. S. Li, H. Lee, F. Li, L. C. Sun, *ChemSusChem* **2018**, *11*, 1761-1767.
[45] Y. H. Liu, Z. Y. Jin, X. Q. Tian, X. Q. Li, Q. Zhao, D. Xiao, *Electrochim. Acta* **2019**, *318*, 695-702.
[46] M. Liu, L. Kong, X. Wang, J. He, X.-H. Bu, *Small* **2019**, *15*, 1903410.
[47] F. G. Chen, L. Y. Zhang, H. Q. Wu, C. Guan, Y. Yang, J. Qiu, P. B. Lyu, M. Li, *Nanotechnology* **2019**, *30*, 32.
[48] X. Teng, L. X. Guo, L. L. Ji, J. Y. Wang, Y. L. Niu, Z. B. Hu, Z. F. Chen, *ACS Appl. Energ. Mater.* **2019**, *2*, 5465-5471.
[49] D. Lim, E. Oh, C. Lim, S. E. Shim, S. H. Baeck, *Catal. Today* **2020**, *352*, 27-33.
[50] L. Q. Ji, Y. Kong, C. Wang, H. Tan, H. L. Duan, W. Hu, G. N. Li, Y. Lu, N. Li, Y. Wang, J. Tian, Z. M. Qi, Z. H. Sun, F. C. Hu, W. S. Yan, *ACS Catal.* **2020**, *10*, 5691-5697.
[51] W. Moschkowitsch, K. Dhaka, S. Gonen, R. Attias, Y. Tsur, M. C. Toroker, L. Elbaz, *ACS Catal.* **2020**, *10*, 4879-4887.
[52] M. Zhang, Y. Q. Liu, B. Y. Liu, Z. Chen, H. Xu, K. Yan, *ACS Catal.* **2020**, *10*, 5179-5189.
[53] M. Zhao, H. L. Li, W. Y. Yuan, C. M. Li, *ACS Appl. Energ. Mater.* **2020**, *3*, 3966-3977.
[54] Y. Kong, J. Li, Y. Wang, W. Chu, Z. Q. Liu, *Catal. Lett.* **2020**, doi.org/10.1007/s10562-10020-03179-y.
[55] H. T. He, J. Gu, X. M. Liu, D. L. Yang, Y. Zhu, R. Yao, Q. Fan, R. S. Huang, *Catalysts* **2020**, *10*, 431.
[56] Y. Lin, J. L. Wang, D. L. Cao, Y. Q. Gong, *Sustain. Energ. Fuels* **2020**, *4*, 1933-1944.
[57] W. J. Zhu, G. X. Zhu, J. Hu, Y. Zhu, H. Chen, C. L. Yao, Z. X. Pi, S. W. Zhu, E. Y. Li, *Inorg. Chem. Comm.* **2020**, *114*, 107851.
[58] R. A. Raimundo, V. D. Silva, E. S. Medeiros, D. A. Macedo, T. A. Simoes, U. U. Gomes, M. A. Morales, R. M. Gomes, *J. Phys. Chem. Solids* **2020**, *139*, 109325.
[59] M. X. Chen, S. L. Lu, X. Z. Fu, J. L. Luo, *Adv. Sci.* **2020**, *7*, 1903777.
[60] S. Si, H. S. Hu, R. J. Liu, Z. X. Xu, C. B. Wang, Y. Y. Feng, *Int. J. Hydrogen Energy* **2020**, *45*, 9368-9379.
[61] S. Liang, B. Wei, M. K. Yuan, Y. Li, X. Ma, Y. Y. Wu, L. L. Xu, *Chemistryselect* **2020**, *5*, 3062-3068.
[62] L. Xu, L. L. Cao, W. Xu, Z. H. Pei, *Appl. Sur. Sci.* **2020**, *503*.
[63] X. L. Zhang, R. L. Liu, C. Y. Tao, S. S. Wu, F. Huang, H. W. Wang, *J. Alloys Comp.* **2020**, *813*, 152219.
[64] S. Thoufeeq, P. K. Rastogi, S. Thomas, A. Shravani, T. N. Narayanan, M. R. Anantharaman, *Chemistryselect* **2020**, *5*, 1385-1395.
[65] X. Li, M. L. Fan, D. N. Wei, X. L. Wang, Y. L. Wang, *J. Electrochem. Soc.* **2020**, *167*, 037555.
[66] X. Q. Liang, Y. H. Li, H. Fan, S. J. Deng, X. Y. Zhao, M. H. Chen, G. X. Pan, Q. Q. Xiong, X. H. Xia, *Nanotechnology* **2019**, *30*, 484001.
[67] Y. Q. Feng, X. Wang, P. P. Dong, J. Li, L. Feng, J. F. Huang, L. Y. Cao, L. L. Feng, K. Kajiyoshi, C. R. Wang, *Sci Rep* **2019**, *9*, 11.

[68] L. M. Ren, C. Wang, W. Li, R. H. Dong, H. X. Sun, N. Liu, B. Y. Geng, *Electrochim. Acta* **2019**, *318*, 42-50.
[69] M. Zhou, Q. H. Weng, X. Y. Zhang, X. Wang, Y. M. Xue, X. H. Zeng, Y. Bando, D. Golberg, *J. Mater. Chem. A* **2017**, *5*, 4335-4342.
[70] B. Q. Li, S. Y. Zhang, C. Tang, X. Y. Cui, Q. Zhang, *Small* **2017**, *13*.
[71] M. Chatti, A. M. Glushenkov, T. Gengenbach, G. P. Knowles, T. C. Mendes, A. V. Ellis, L. Spiccia, R. K. Hocking, A. N. Simonov, *Sustain. Energy Fuels* **2018**, *2*, 1561-1573.
[72] J. H. Yu, G. Z. Cheng, W. Luo, *J. Mater. Chem. A* **2017**, *5*, 15838-15844.
[73] X. Xu, F. Song, X. L. Hu, *Nat. Commun.* **2016**, *7*, 12324.
